# Alterations in the Immune‐Fibrotic Axis Are Spatially Localized in the Aging Heart

**DOI:** 10.1111/acel.70768

**Published:** 2026-11-05

**Authors:** Aleksandra Aljakna Khan, Amalia Sintou, Yue Zhao, Antonios Somarakis, Harshpreet Chandok, Juliana Alcoforado Diniz, Ramalakshmi Ramasamy, Gretchen T. Clark, Leon Frommer, Sikander Hayat, Brian S. White, Paul Robson, Ron Korstanje, J. Matthew Mahoney, Nadia A. Rosenthal

**Affiliations:** ^1^ The Jackson Laboratory Bar Harbor Maine USA; ^2^ The Jackson Laboratory for Genomic Medicine Farmington Connecticut USA; ^3^ Department of Medicine 2 RWTH Aachen University, Medical Faculty Aachen Germany; ^4^ Cardiovascular Research Institute and Department of Medicine Icahn School of Medicine at Mount Sinai New York New York USA; ^5^ Windreich Department of Artificial Intelligence and Human Health Icahn School of Medicine at Mount Sinai New York New York USA; ^6^ National Heart and Lung Institute Imperial College London UK

**Keywords:** aging, aorta, fibrosis, inflammaging, inflammation, RNA‐seq, spatial transcriptomics, valves

## Abstract

The immune‐fibrotic axis functions as a vicious feedback loop where age‐related chronic, low‐grade systemic inflammation (inflammaging) drives maladaptive tissue repair and fibrosis, while senescent fibrotic cells in turn amplify systemic inflammation. In the aging heart, immune dysregulation and fibroblast activation drive tissue scarring and stiffening and a higher risk of heart failure and vascular disease. To identify cardiac regions and cell types affected with age, we monitored transcriptional changes across whole hearts of 6‐ and 24‐month‐old male and female mice. Spatially resolved signatures of macrophages and fibroblasts, non‐cardiomyocyte populations highly involved in inflammaging, were enriched predominantly in the valves and aorta. With age, two cell types simultaneously adopted maladaptive programs: Macrophages displayed reduced immune homeostasis together with increased chemotactic and stromal programs, while more widely distributed fibroblast‐lineage cells displayed alterations in extracellular matrix organization and intercellular signals, as well as upregulation of senescence‐associated *Cdkn1a* (encoding p21) and other activated features shared with human cardiac fibroblasts. The major biological significance of these findings is that cardiac immune‐fibrotic remodeling is not a uniform, heart‐wide process. Instead, it varies with cell type, with valves and aorta emerging as major sites where age‐associated programs converge to progressively compromise cardiac structure and function.

## Graphical abstract

Aging does not affect the heart uniformly: unique changes in immune cells (macrophages) and scar‐forming cells (fibroblasts) first appear in the valves and aorta and spread into the heart muscle with age. Aged fibroblasts also develop features shared with human heart fibroblasts that may contribute to declining cardiovascular health.

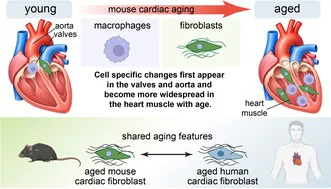

## 1 Introduction

Aging poses one of the major risk factors for cardiovascular disease. In the mammalian heart, a persistent inflammatory state known as inflammaging is promoted by various repeated antigenic stimuli throughout life, driving the release of chronic low‐grade inflammatory signals (Ferrucci and Fabbri 2018). Cardiovascular inflammaging is associated with fibrosis, vascular dysfunction and impaired tissue homeostasis that together promote adverse cardiac remodeling, atherosclerosis, and heart failure (Chirinos et al. 2019; Liberale et al. 2025). Age‐related changes in cardiomyocyte function (Yucel et al. 2026) and the perivascular environment (Rodriguez Morales et al. 2025) include the accumulation of cells with senescence‐associated features defined by a set of hallmarks that includes cell‐cycle arrest (Abdellatif et al. 2023; Gorgoulis et al. 2019; SenNet Consortium 2022).

Cardiovascular inflammaging is driven primarily by a shift in immune and resident stromal cell interactions. In the adult heart, resident macrophages contribute to maintaining homeostasis by clearing pathogens and debris and influence fibrosis by interacting with other cells through antigen presentation, cytokine and chemokine signaling (Nicolas‐Avila et al. 2022). With advanced age, self‐sustaining protective resident macrophages are replaced by aggressive bone marrow‐derived macrophages that stoke chronic tissue inflammation. Adult cardiac fibroblasts retain an organ‐specific embryonic transcriptome (Forte et al. 2022; Furtado et al. 2016) and are normally responsible for routine extracellular matrix (ECM) maintenance by responding to biochemical and mechanical signals within the myocardium (Pesce et al. 2023; Tallquist and Molkentin 2017). In aged hearts, cardiac fibroblasts are induced by pro‐inflammatory macrophages to become chronically overactive, secreting cytokines and matrix‐remodeling proteins and driving localized fibrosis that recruits more immune cells, which accelerates tissue stiffening and functional decline (Frangogiannis 2019).

Collectively, inflammaging affects coordination among cardiac cell populations: activated macrophages contribute to chronic low‐grade sterile inflammation, fibroblast mechano‐sensing and responsiveness to growth factors are altered, and vascular endothelial cells progressively lose metabolic and vascular homeostatic functions (Angelini et al. 2023; Ferrucci and Fabbri 2018; Pesce et al. 2023). In humans, features of cardiovascular inflammaging are also sex‐related: men generally experience an earlier and more linear functional decline, whereas women often exhibit accelerated vascular dysfunction and fibrosis after menopause, contributing to a higher prevalence of heart failure with preserved ejection fraction (Fopiano et al. 2025).

Single‐cell and spatial transcriptomic mouse heart atlases have already documented age‐related transcriptional changes in multiple cell types (Morsy et al. 2026; Schaum et al. 2020; Tabula Muris Consortium 2020; Tabula Muris Consortium et al. 2018; Vidal et al. 2019). Large‐vessel and perivascular niches in the aging left ventricle were specifically identified as prominent sites of inflammatory, fibrotic, and senescence‐associated changes (Rodriguez Morales et al. 2025). To extend these observations, we monitored the aging response of the entire heart using bulk RNA‐seq and characterized macrophages and fibroblast‐lineage cells using single nucleus RNA‐seq and spatial transcriptomics in 6‐ and 24‐month‐old C57BL/6J male and female mouse hearts (Figure S1). Spatial transcriptomic analysis of longitudinal cardiac sections encompasses all four chambers, valves, and proximal great vessels. Our findings shift the focus from distributed sites of aging throughout the myocardium toward sites of cardiac connective tissue and large‐vessel compartments, implicating valves and aorta as niches where the strongest inflammaging‐related changes are localized. Interestingly, resulting senescence‐associated transcriptional signatures from young and aged fibroblasts partially shared features also found in the human heart.

## 2 Methods

### 2.1 Mice

All animal experiments were performed according to the NIH Guide for the Care and Use of Laboratory Animals and were approved by The Jackson Laboratory's Animal Care Use Committee. Inbred male and female 6‐ and 24‐month‐old C57BL/6J (*n* = 3 per sex and time point) were obtained from The Jackson Laboratory, maintained at a 12 h (6 AM‐6 PM) light/dark cycle with ad libitum access to water and standard chow (6% fat/kcal, 5KOG LabDiet, St. Louis, MO). Mice were sacrificed between 9 AM and 11 AM by cervical dislocation.

### 2.2 RNA Extraction and Sequencing for Bulk RNA‐Seq

Hearts were placed immediately in RNAlater. Following thawing and mincing, half of the tissue was homogenized in BeadBug 6 prefilled microtubes (Sigma Millipore; two cycles of 90 s at speed 3000). RNA was isolated using RNeasy Plus Mini Kit (Qiagen) per manufacturer's protocol and quantified using Thermo Scientific NanoDrop Spectrophotometer. All samples passed quality control with a RIN value higher than 7 and a sequencing depth of 30 million reads per sample was generated. Libraries were prepared using KAPA RNA HyperPrep Kit Illumina Platforms v1.16, quantified by qPCR (KAPA Library Quantification Kit), and indexed using TruSeq DNA UD Indexes v2 (Illumina). Sequencing was on NovaSeq X Plus Kit (Illumina), a 10B Flow Cell with 8 lanes (300 cycles, 1250 M reads/lane). Full details: https://www.protocols.io/view/jax‐sen‐library‐construction‐using‐kapa‐rna‐hyperp‐kxygx7pjwl8j/v1?step=1.

### 2.3 Bulk RNA‐Seq Data Processing and Analysis

Samples were processed using an internal RNA‐seq pipeline (https://github.com/TheJacksonLaboratory/cs‐nf‐pipelines/wiki/RNA‐Pipeline‐ReadMe) with default parameters. Raw reads were quality‐filtered and adapter‐trimmed using fastp (v0.23.2) and aligned to GRCm38 (Ensembl, v102) using RSEM (v1.3.1) with bowtie2 (v2.4.5). Alignment estimation of gene expression was performed using the Expectation–Maximization algorithm paired‐end read data. RSEM also generated gene‐level and isoform‐level expression estimates, producing gene counts, isoform counts, and BAM files for both genome‐aligned and isoform‐aligned reads. Post‐alignment quality control (QC) was conducted with FastQC (v0.11.9) and Picard CollectRnaSeqMetrics, and compiled using MultiQC. Normalization (trimmed Mean of M‐values) and differential expression analyses were done using the edgeR (v4.6.0), retaining genes with CPM > 1 in ≥ 2 samples and a generalized linear model (quasi‐likelihood F‐test, using sex, age, and their interaction term, FDR < 0.05) (Table S1). Sex was not a major overall driver specifically in the analysis of bulk RNA‐seqdata: 12 DEGs passed FDR < 0.05 with 7 of them on sex‐specific chromosomes (Table S2). Differentially expressed genes (DEGs) were analyzed using KEGG pathway (Kanehisa et al. 2025) with clusterProfiler (v4.16.0) in R and Gene Ontology (GO) terms (Gene Ontology Consortium et al. 2023) (Table S3).

Co‐expressed network analysis used WGCNA R package (Langfelder and Horvath 2008, 2012) on normalized expression data and soft‐thresholding power approximating scale‐free topology. Gene modules were clustered by hierarchical clustering of the topological overlap matrix. Module‐trait relationships were assessed by correlating module eigengenes with age, sex, and their interaction (FDR < 0.05), followed by KEGG and GO enrichment. Out of 32 WGCNA modules tested, only one was significant for age (green module, FDR < 0.05) and none had FDR < 0.05 for sex (Tables S4 and S5).

### 2.4 Nuclei Isolation for snRNA‐Seq

Hearts were snap‐frozen in liquid nitrogen immediately after dissection, stored at −80°C until analysis, processed using 10× Genomics Chromium Nuclei Isolation Kits with RNase Inhibitor (PN‐1000494) following manufacturer's Protocol CG000505 Rev A. Hearts, sectioned into quarters, individually lysed using the Chromium lysis buffer, dissociated with a pestle, and subsequently pooled, mixed, and divided into four columns for further processing per manufacturer's instructions. Nuclei suspensions were combined, filtered through a 40 μm cell strainer and assessed for viability and concentration using the LUNA‐FX7 automated cell counter (Logos Biosystems). Nuclei suspensions with a concentration ranging from 8 × 10^3^ and 2 × 10^5^ nuclei/μL were accepted for downstream analysis.

### 2.5 Single Nuclei Capture, Library Preparation, Sequencing, and Alignment

Single‐nuclei encapsulation, barcoding, and library preparation used the 10× Genomics Chromium X platform per manufacturer's protocol (CG000731_ChromiumGEM‐X_SingleCell3_ReagentKits_v4_UserGuide). cDNA and libraries' quality was assessed by Agilent TapeStation 4200 and Qubit Fluorometer (ThermoFisher) and quantified using KAPA qPCR; sequencing was done on an Illumina NovaSeq X+ 10B with a 100‐cycle flow cell (28‐10‐10‐90 asymmetric read configuration), targeting ~8000 barcoded cells/sample, with an average 50,000 reads/cell. Illumina BCL files were converted to FASTQ using ‘bcl2fastq’ v2.20.0.422, which were aligned to GRCm38 (GENCODE vM23, 10× Genomics mm10 reference 2020‐A) using the Cell Ranger ‘count’ pipeline (v8.0.0 or v9.0.1).

### 2.6 Mouse snRNA‐Seq Analysis

Nuclei were retained if they expressed > 200 genes, contained < 20% mitochondrial and < 25% ribosomal reads, and excluded if > 25,000 UMIs and expressed > 5000 genes; only genes detected in at least three nuclei were retained. Mitochondrial genes were excluded. After all QC steps, 116,002 high‐quality nuclei were retained. Further downstream analyses were conducted in the R environment using Seurat v4.0.1 (Satija et al. 2015). Doublets were removed using DoubletFinder (v2.0.3) with default parameters (McGinnis et al. 2019). Log‐transformation, normalization, dimensionality reduction via principal component analysis (PCA), and clustering were done in R (Seurat v4.0.1, resolution = 2); the first 50 principal components were used for UMAP embedding (n_neighbors and min_dist were set to default values) (Satija et al. 2015). Cell types were identified using cell‐specific marker genes (Wilcoxon rank‐sum test) as previously published; fibroblast subtypes were annotated based on three gene markers *Gsn* (stromal fibroblasts), *Postn* (specialized differentiated fibroblasts), and *Dkk3* (fibroblasts characterized by a gene signature closely related to valve leaflets) (Forte et al. 2020; Jana et al. 2021). GO BP analyses were performed separately on significantly upregulated and downregulated DEGs using EnrichR (https://maayanlab.cloud/Enrichr/) for macrophages, *Gsn* ^+^ fibroblasts, and endothelial cells (adj. *p* < 0.05; |log2FC| > 0.36, Tables S6–S11). DEG analysis was done between *Cdkn1a* ^+^ and *Cdkn1a* ^−^ fibroblast‐lineage cells; young and aged samples combined, stratified by sex (*n* = 6 per sex; using the Wilcoxon rank‐sum test; adjusted *p*‐value < 0.05 and |log2FC| > 0.36). *Cdkn1a* ^+^ and *Cdkn1a* ^−^ nuclei were defined as those with non‐zero and zero normalized *Cdkn1a* expression, respectively. An interactive browser for the snRNA‐seq dataset is in development (https://jackson‐aging‐heart‐site.vercel.app/). It allows exploration of cell‐type‐specific gene expression and age‐associated changes across the 16 annotated cell types (116,002 nuclei from 6‐ and 24‐month‐old hearts; *n* = 6 per age group; sex‐balanced), along with derived cell‐level summaries including pathway and transcription factor activity, senescence scores, cell composition, and cell–cell communication. Analyses presented in the browser are exploratory and computed across nuclei; they are not part of the confirmatory analyses reported here.

### 2.7 Cell Proportion Comparison

Cell‐type proportions (young (p1) vs. aged (p2)) were compared using a two‐proportion *Z*‐test (independently for males and females) (Su 2024). Under the null hypothesis of no difference between proportions, the computed *Z*‐statistics follow a standard normal distribution, allowing for calculation of *p*‐values to assess statistical significance.

### 2.8 FFPE Tissue Embedding

Hearts were fixed in 10% Neutral Buffered Formalin (24 h), processed overnight (Citadel 2000 Tabletop Processor), embedded in paraffin and stored wrapped in foil at 4°C for RNAse‐free sectioning for spatial transcriptomics.

### 2.9 FFPE Block DV200 Evaluation

FFPE cardiac sections were cut in the long‐axis (longitudinal) plane. Two to four 5 μm FFPE sections were obtained in RNAse‐free conditions. RNA was extracted using Qiagen's RNeasy Micro Kit (Cat. No./ID: 74004) and quantified using a Tapestation High Sensitivity RNA ScreenTape (Agilent). DV200 scores (% RNA sample > 200 bases in length, Agilent TapeStation Analysis software) > 50% was achieved for all sections.

### 2.10 Visium Spatial Transcriptomics Library Preparation, Sequencing, Data Pre‐Processing, and Alignment

Each slide containing FFPE section (5 μm, ColorFrost Plus slides by Fisher) with the region of interest (ROI, 6.5 mm × 6.5 mm) was deparaffinized and hybridized with manufacturer‐provided mouse‐specific probe sets for mRNA labeling and transferred using the CytAssist (10× Genomics) onto a Visium CytAssist Slide; libraries were prepared per 10× Genomics Protocol CG000495, quantified using a Tapestation High Sensitivity DNA ScreenTape (Agilent) and fluorometry (Thermofisher Qubit), and verified via KAPA qPCR. The library final yield and concentrations ranges were 90–275 ng and 2.85–7.55 ng/μL, respectively. Pooled libraries were sequenced on an Illumina NovaSeq X+ 10B 100 cycle flow cell (28‐10‐10‐90 read configuration), targeting 100,000 read pairs/spot. Illumina BCL files for all libraries were converted to FASTQs (bcl2fastq v2.20.0.422, Illumina). Slides were H&E stained (The JAX Histology Core; details online https://doi.org/10.17504/protocols.io.6qpvr3653vmk/v1), scanned (40X, Nanozoomer, HT2.0, Hamamatsu), and analyzed by NDP software. For each tissue section and corresponding library, the whole slide brightfield and CytAssist images were aligned manually using the Loupe Browser (v6.4.1) via landmark registration. Each whole slide image was uploaded to a local OMERO server where a rectangular ROI containing just tissue was drawn via OMERO.web. OMETIFF images of each ROI were programmatically generated using the OMERO Python API. FASTQ files, the image registration JSON file, and associated OMETIFF corresponding to high‐resolution brightfield image were further processed, including alignment to the pre‐built mouse transcriptome reference m10‐2020‐A‐specific filtered probeset (v1.0.0) using Space Ranger count pipeline (v2.1.0). Following alignment with Space Ranger, the number of spots under tissue ranged from 2373 to 3973 across the samples. Sequencing depth yielded with a cohort‐wide average of 5452.75 ± 2157.41 median genes per spot and of 33,079.00 ± 24,316.42 median UMI counts per spot.

### 2.11 Visium Spatial Transcriptomics Data Analysis

Data were pre‐processed using Space Ranger and analyzed in Seurat (v5.1.0) (Stuart et al. 2019). Spots were filtered using the StarDist tool (Stevens et al. 2022) by retaining only nuclei‐containing spots. Expression data were then normalized using the SCTransform workflow implemented in Seurat (Hafemeister and Satija 2019). Robust Cell Type Decomposition (RCTD, v2.2.1) (Cable et al. 2022) was applied to map cell‐type composition across all 12 Visium samples, using raw snRNA‐seq counts and corresponding cell‐type annotations as reference for the deconvolution analysis. In total, 88,704 nuclei from 19 distinct cell types were used as the reference. RCTD computes the mean gene expression profile for each annotated cell type in the reference dataset and subsequently uses these profiles to model each spatial transcriptomics spot as a mixture of cell types (without limiting their number in full doublet mode) and estimates the proportion of each cell type per spot. Significance of spatial enrichment for specific cell types or genes was assessed using permutation testing within each sample, then combined *p*‐values across demographic groups using Fisher's method (FDR‐corrected).

Cardiac regions (left and right ventricles, septum, atria, aorta, valves) in each heart section were manually annotated by consensus of 2 cardiovascular researchers. Published mouse heart‐specific senescence gene modules (Sanborn et al. 2025) were tested in this study in aged aortas. Expression of each gene (per sample) from the module was tested using a randomly generated null distribution, with subsequent *p*‐values aggregation across demographic groups (male/female, young/old; Fisher's method corrected for multiple testing), and visualization on a heatmap. DEG analysis was performed using FindMarkers in Seurat (v5.1.0), which performs a Wilcoxon Rank Sum test (min.pct = 0.1, logfc.threshold = 0, adj. *p*‐value < 0.05 and avglog2FC > 0) to compare macrophage‐containing spots with vs. without *Ccl8*, and *Ccl8*‐expressing macrophages vs. *Ccl8*‐expressing non‐macrophage cells. GO analysis was done with clusterprofiler (v4.14.6) (FDR < 0.05).

### 2.12 Human snRNA‐Seq and Cross‐Species Integration

Human cardiac snRNA‐seq data was obtained from previously approved and publicly available datasets of non‐failing hearts from men and women of various ages (Bleckwehl et al. 2025). Cross‐species label transfer and *CDKN1A* expression analyses were performed in R (v4.4.1, Rocky Linux 8.10) using Seurat (v5.2.0), SeuratObject (v5.0.2), Symphony (v0.1.2), babelgene (v22.9), biomaRt (v2.62.1), dplyr (v1.1.4), purrr (v1.0.2), future (v1.34.0), future apply (v1.11.3), ggplot2 (v3.5.1), and pheatmap (v1.0.12). Mouse snRNA‐seq counts were converted into human gene identifier space by mapping mouse genes to their human orthologs (babelgene, only unique 1:1 orthologs). Matrices were restricted to shared genes and processed with Seurat (log‐normalization, selection of 3000 variable features, scaling, and PCA). A batch‐corrected human reference was constructed with patient identity as the integration covariate (Symphony, 3000 variable genes, 30‐dimensional latent space, UMAP for visualization). Mouse nuclei were projected into this reference and assigned human cell‐type labels (Annotation_2) via k‐nearest neighbor classification (*k* = 5). Concordance was quantified using confusion matrices and heatmaps, and visualized on the shared UMAP. Normalized *CDKN1A* expression was used to define *CDKN1A*‐positive cells (non‐zero expression) and calculate their proportion for each species and unified cell type (summarized in tabular form and barplots).

### 2.13 Differential Gene Expression Between *CDKN1A* ‐Positive and Negative Fibroblast‐Lineage Cells

Mouse *Gsn* ^+^, *Dkk3* ^+^, and *Postn* ^+^ fibroblasts (24,972 nuclei) and human fibroblast‐lineage nuclei (93,916 nuclei; all subclusters annotated as fibroblasts in the human atlas, including the two myofibroblast subclusters) were pooled per species. Nuclei with at least one *Cdkn1a/CDKN1A* UMI were defined as positive (mouse 2030 positive vs. 22,942 negative; human 4695 vs. 89,221). Counts were normalized to 10,000 per nucleus and log‐transformed (Seurat v5.2.0). Differential expression between *CDKN1A*‐positive and *CDKN1A*‐negative nuclei was performed with MAST (v1.32.0) via Seurat FindMarkers, including age, sex, sample (mouse) or patient cluster (human) and the cellular detection rate (scaled number of genes detected per nucleus) as covariates. Genes with Bonferroni‐adjusted *p* < 0.05 and |log2FC| > 0.25 were considered differentially expressed. GO BP over‐representation was computed separately for up‐ and downregulated genes with clusterProfiler (v4.14.4) against MSigDB C5 GO:BP gene sets (msigdbr v7.5.1), using all tested genes as background and Benjamini‐Hochberg correction (adjusted *p* < 0.05).

## 3 Results

### 3.1 Age‐Induced Cardiac Transcriptional Signatures Are Associated to Inflammaging

To obtain a comprehensive overview of cardiac age‐related changes in gene expression, we compared bulk RNA‐seq from 6‐ and 24‐month‐old C57BL/6J mouse hearts (both sexes) and identified 287 differentially expressed genes (DEGs, 183 up‐ and 104 down‐regulated) (Table S1, Figure S2). Inflammation and ECM remodeling were identified as the major signatures of aging, including changes in ECM‐receptor interaction, complement and antigen presentation (Figure 1A, Table S3). Weighted gene co‐expression network analysis (WGCNA) identified a single significant age‐associated module enriched in inflammatory and immune processes, including myeloid leukocyte activation and TNF‐associated cytokine production, together with cellular stress and ion‐regulatory responses (Figure 1B–D, Table S5).

**FIGURE 1:**
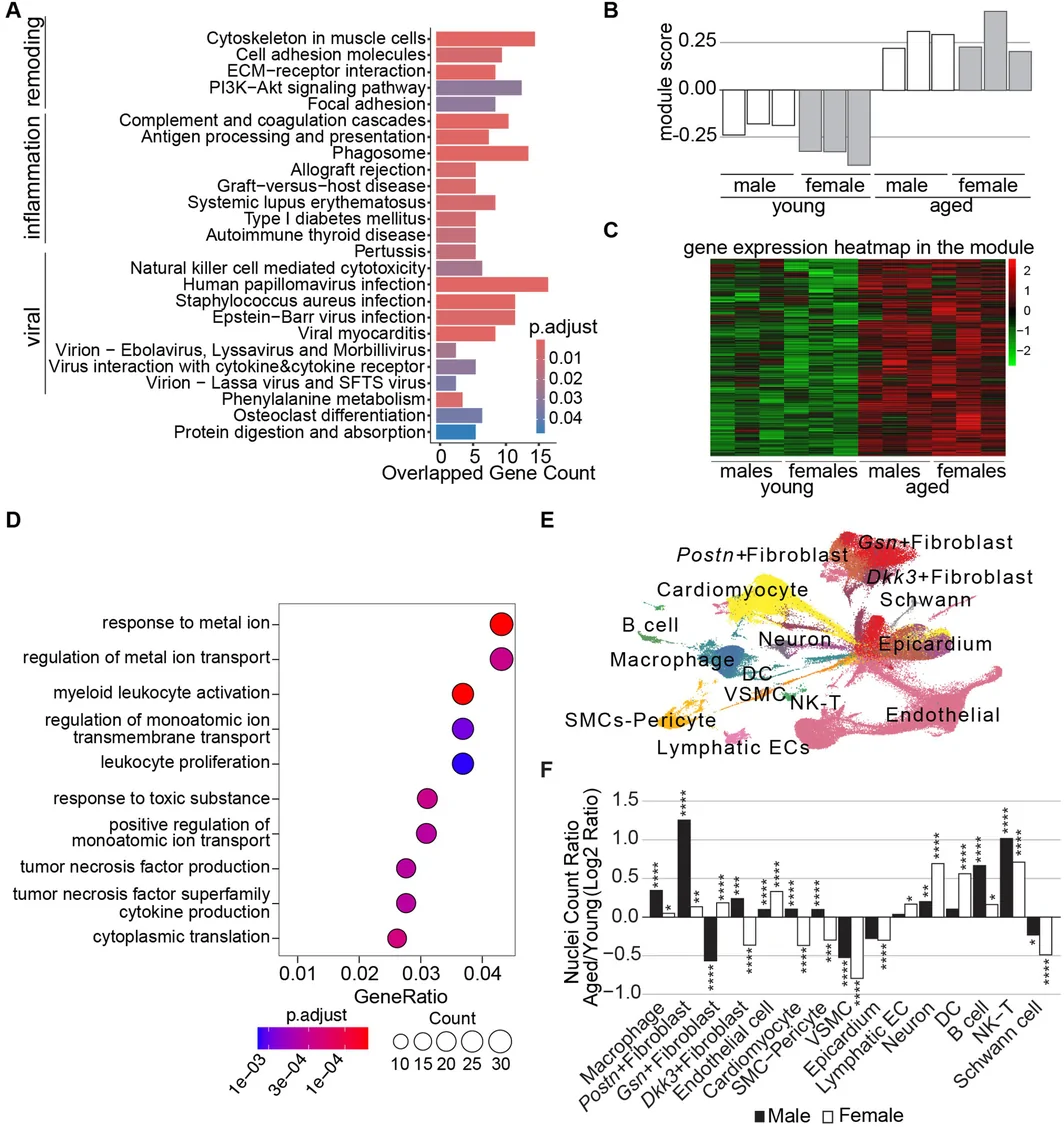
Age induces cardiac inflammation and remodeling changes and increases number of macrophages and *Postn* ^+^ fibroblasts. (A) Enriched KEGG pathways based on significant bulk RNA‐seq DEGs between young (6 m) and aged (24 m) mice (males and females combined; *n* = 3 per sex/age); FDR < 0.05; color scale (blue to red)—adjusted *p* value. (B) Significant age‐related module from weighted gene co‐expression network analysis (WGCNA); FDR < 0.05 (*n* = 3 per sex/age). (C) Expression heatmap of genes in the module from (B); color scale (green‐black‐red)—gene expression (lcpm‐log of counts per million). (D) Enriched GO BP (FDR < 0.05) based on genes in the age‐related WGCNA module. (E) Cardiac nuclei segregated according to cell types on the uniform manifold approximation and projection (UMAP) embedding plot; combined young (6 m) and aged (24 m) males and females (*n* = 3 per sex/age); each point is a single nucleus, color represents cell type, Wilcoxon rank‐sum test. (F) Age‐related changes in cell composition: Nuclei count ratios (log2 ratio, aged/young) for each cell type in males and females; two‐proportion *Z*‐test (*p < 0.05, **p < 0.01, ***p < 0.001).

To investigate age‐related transcriptional changes in cardiac cells specifically implicated in inflammaging, we compared snRNA‐seq profiles of cells from 6‐ versus 24‐month‐old whole hearts (Figure 1E). Cells were manually annotated using previously published markers (Figure S3). Age‐related changes in cell‐type composition were assessed by comparing nuclei proportions between aged and young hearts in males and females. Macrophages and *Postn* ^+^ fibroblasts increased with age, while *Gsn* ^+^ fibroblasts showed a modest increase in females but not in males (Figure 1F). Overall macrophage population was annotated using *C1qa*, encoding the A‐chain polypeptide of complement subcomponent C1q and a core biomarker to diverse populations of macrophages. Several established macrophage markers were examined in this population, and it was characterized by high expression of *C1qc*, *Lyz2*, *Ctsb*, *Csf1r* and low expression of *Ccr2* (Figure S4). Gelsolin (*Gsn*) is a calcium‐regulated protein that binds to actin filaments. *Gsn* ^+^ fibroblasts were selected for further age‐associated analysis, given their established role in ECM production, marked by expression of decorin (*Dcn*) and collagen (*Col1a1*) (Forte et al. 2020). In line with this, *Gsn* ^+^ and *Postn* ^+^ fibroblasts formed a transcriptional continuum, with aging shifting cells toward the *Postn* ^+^ fibroblast‐associated state in both sexes, without implying a uniform or irreversible transition (Figure S5).

### 3.2 Inflammaging Markers Are Localized to Valves and Aorta

To characterize the anatomical organization of cardiac cell populations specifically associated with inflammaging, we analyzed sections of 6‐ and 24‐month male and female mouse hearts encompassing all four cardiac chambers together with the valves, aortic root, and proximal great vessels. Aorta and valve areas had the highest estimated proportion of macrophages and *Gsn* ^+^ fibroblasts, as revealed by inferring proportional representation using deconvolution analysis that combines cell‐type signatures from snRNA‐seq data and spatial transcriptomic data (Visium, 10× Genomics) (Figure 2) (Cable et al. 2022).

**FIGURE 2:**
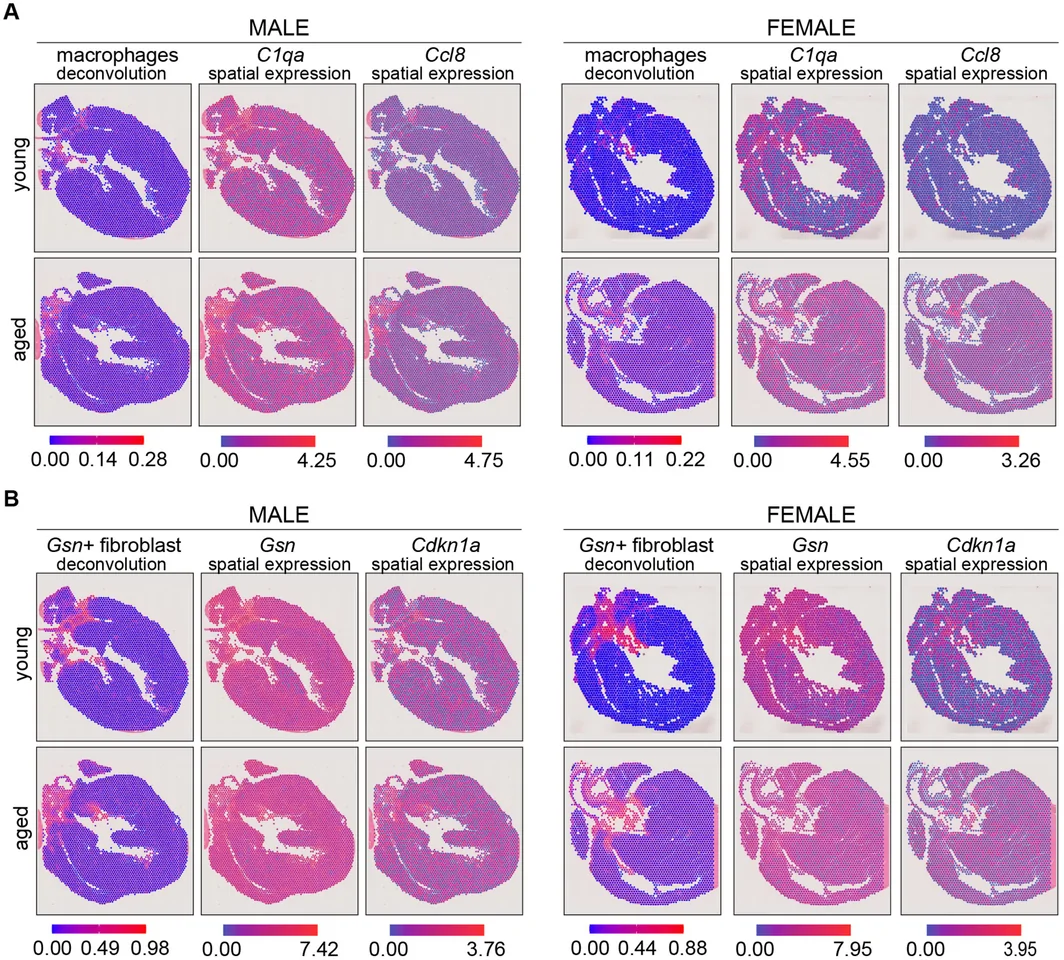
Aorta and valve areas had the highest estimated proportion of macrophages and *Gsn* ^+^ fibroblasts. Spot deconvolution of macrophages (A) and *Gsn* ^+^ fibroblasts (B) using snRNA‐seq transcriptional profiles as a reference to infer cell proportions (blue to red color scale—estimated proportions) as well as spatial expression of cell markers (*C1qa*—macrophages (A); *Gsn*—*Gsn* ^+^ fibroblasts (B)), *Ccl8* (A) and *Cdkn1a* (B) using spatial transcriptomics data (Visium, 10× Genomics) (blue to red color scale—gene expression); representative examples, *n* = 3 sex/age.

Spatial analysis also enabled evaluation of gene expression and cell‐type composition across cardiac regions. Localization of general markers for macrophages in the valves and aorta of male and female hearts showed no significant changes in 24‐month old hearts (Figure 2A). Specifically, localized expression of *C1qa* was not significantly affected by age. However, expression of C‐C motif chemokine ligand 8 (*Ccl8*) became more prominent in the myocardium in both male and female hearts by 24 months (Figure 2A). Notably, *Ccl8* is a key component of the senescence‐associated secretory phenotype (SASP) and robustly upregulated in senescent cells and aging tissues, driving chronic inflammation, immune cell recruitment, and tissue dysfunction (Sanborn et al. 2025).

### 3.3 Aged Macrophages Acquire Stromal‐Associated and Inflammatory Transcriptional Changes

Further analysis of age‐associated changes across the macrophage compartment highlighted increased engagement of aged macrophages in stromal and vascular remodeling. In GO BP enrichment analysis of upregulated DEGs from the snRNA‐seq dataset, aged macrophages were enriched in biological processes such as growth factor and receptor signaling, including ERK1/2, PDGF‐, and VEGF‐associated signaling, concomitant with regulation of fibroblast proliferation and smooth muscle cell migration (Figure 3A). Analysis per‐sex revealed weaker enrichment of MAPK/Ras signaling and fibroblast proliferation‐related processes in males, while females were enriched for muscle‐ and cardiac morphogenesis‐associated processes (Figure 3B,C).

**FIGURE 3:**
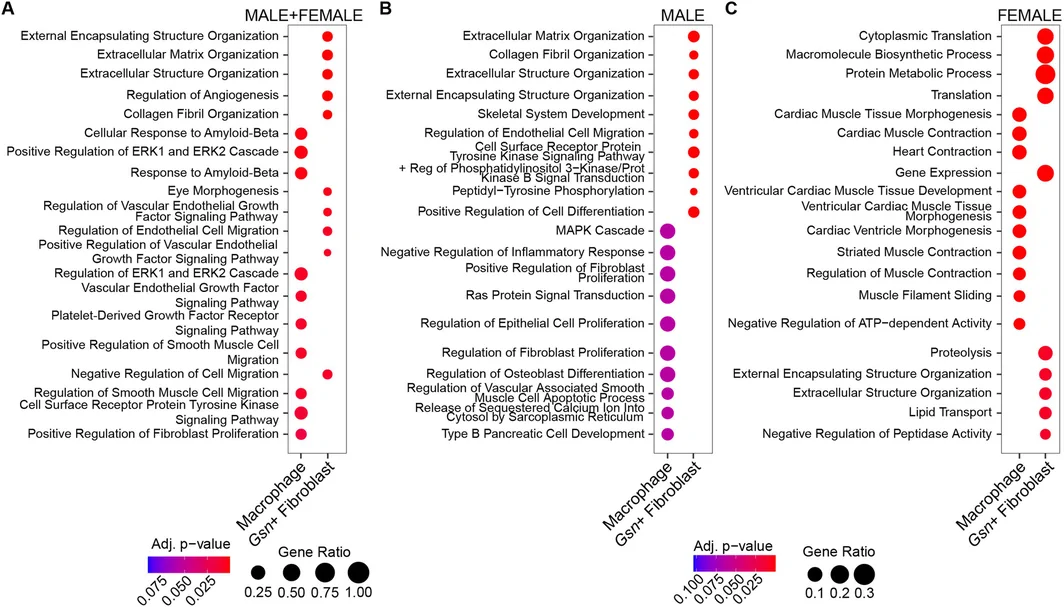
Aging macrophages and *Gsn* ^+^ fibroblasts acquire stromal‐associated and ECM remodeling transcriptional changes, respectively. Dot plots show top ten enriched GO BP (adj. *p*‐value < 0.05) based on significantly upregulated DEGs with aging (young vs. aged, *n* = 3 per age/sex, adj. *p*‐value < 0.05, log2FC > 0.36) in both sexes (A), males only (B), and females only (C). Dot color indicates the adjusted *p*‐value (red = more significant); dot size reflects the gene ratio (proportion of upregulated DEGs annotated to that term), per the scales shown below each panel with males and females only on the same scale.

*Ccl8* provided a link between the whole heart and spatial datasets. It was an age‐associated DEG in bulk RNA‐seq (Table S1) and belonged to the age‐related WGCNA module (Table S5). *Ccl8*‐expressing regions in the aorta were associated with granulocyte chemotaxis, myeloid leukocyte migration, humoral immune responses, ECM organization, cell‐substrate adhesion, wound healing, connective tissue development, and phagocytosis (Figures 4A–C, S6 and S7). These associations identify *Ccl8* macrophages as an immune‐associated component of the broader whole‐heart aging response, consistent with inflammatory recruitment and local tissue remodeling.

**FIGURE 4:**
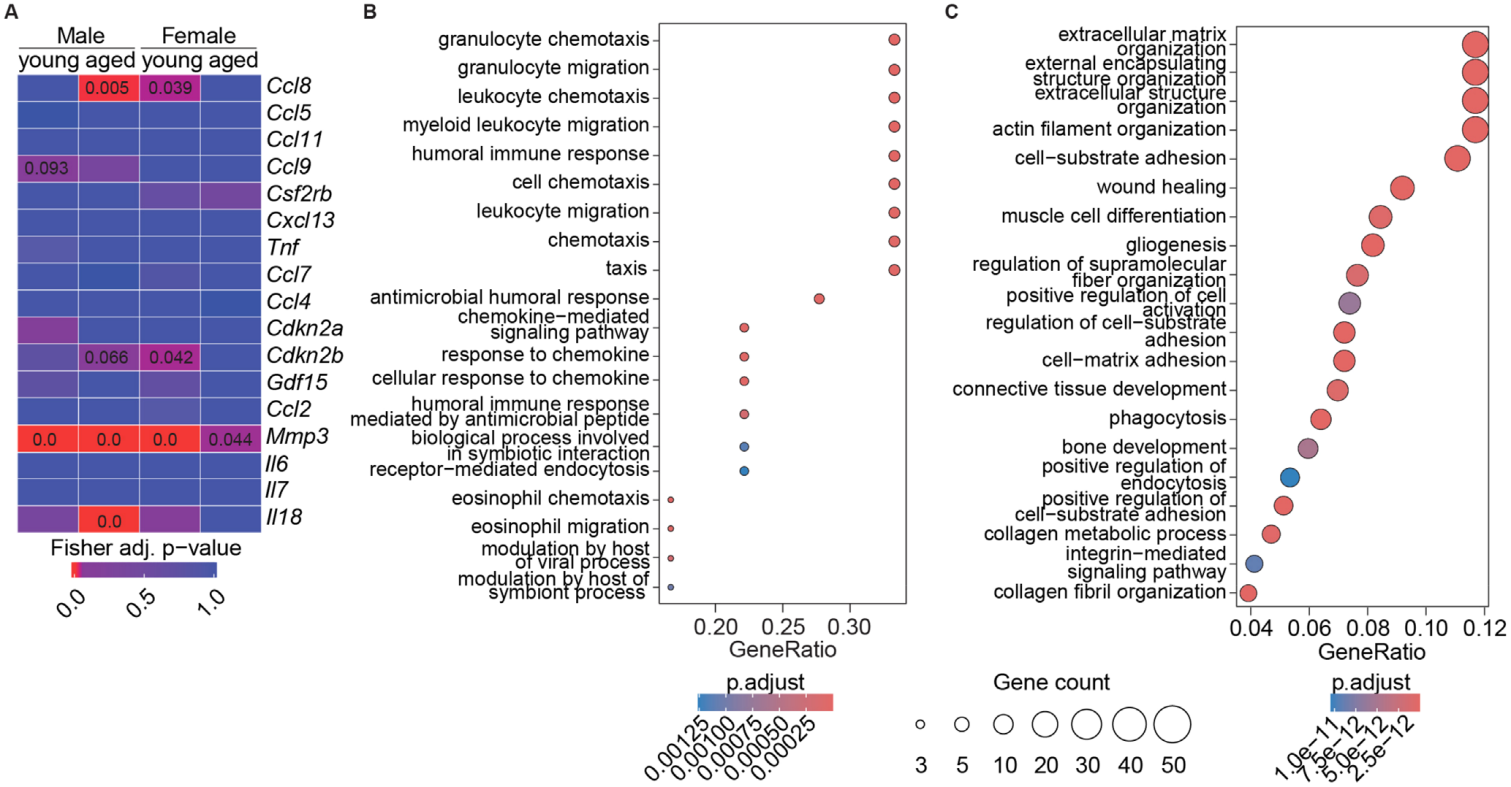
*Ccl8* is significantly correlated with aged male aorta and is associated with inflammation and ECM remodeling. (A) Permutation‐based significance testing of 17 senescence‐associated genes in aortic spots, with *p*‐values aggregated using Fisher's method (young (*n* = 1) and aged (*n* = 3) males; young (*n* = 1) and aged (*n* = 1) females); color scale (blue to red)—FDR corrected *p* value. (B, C) Enriched GO BP based on DEGs were calculated within macrophage‐enriched spots between *Ccl8^+^* and *Ccl8^−^* spots in (B) and within *Ccl8^+^* spots, between macrophage‐enriched and non‐enriched spots in (C). Dot size reflects gene count; color scale (blue to red)—adjusted *p*‐value.

### 3.4 Aged Fibroblasts Are Associated With ECM Remodeling

Gene markers of fibroblast lineages and macrophages were expressed in same spots in valves and aorta (Figure 2). *Gsn* is a gene marker of fibroblast‐lineage cells co‐expressed with *Cdkn1a* (encoding p21, a cell cycle inhibitor associated with senescence) within the same spots in the aorta and valves, spatially locating *Cdkn1a* expression to fibroblast‐lineage‐enriched regions identified in deconvolution analysis (Figures 2B and 5B). Whereas *Gsn* expression in the valves and aorta of male and female hearts showed no significant changes in 24‐month hearts (Figure 2B), expression of *Cdkn1a* became more pronounced in the myocardium in both male and female hearts by 24 months (Figure 2B).

**FIGURE 5:**
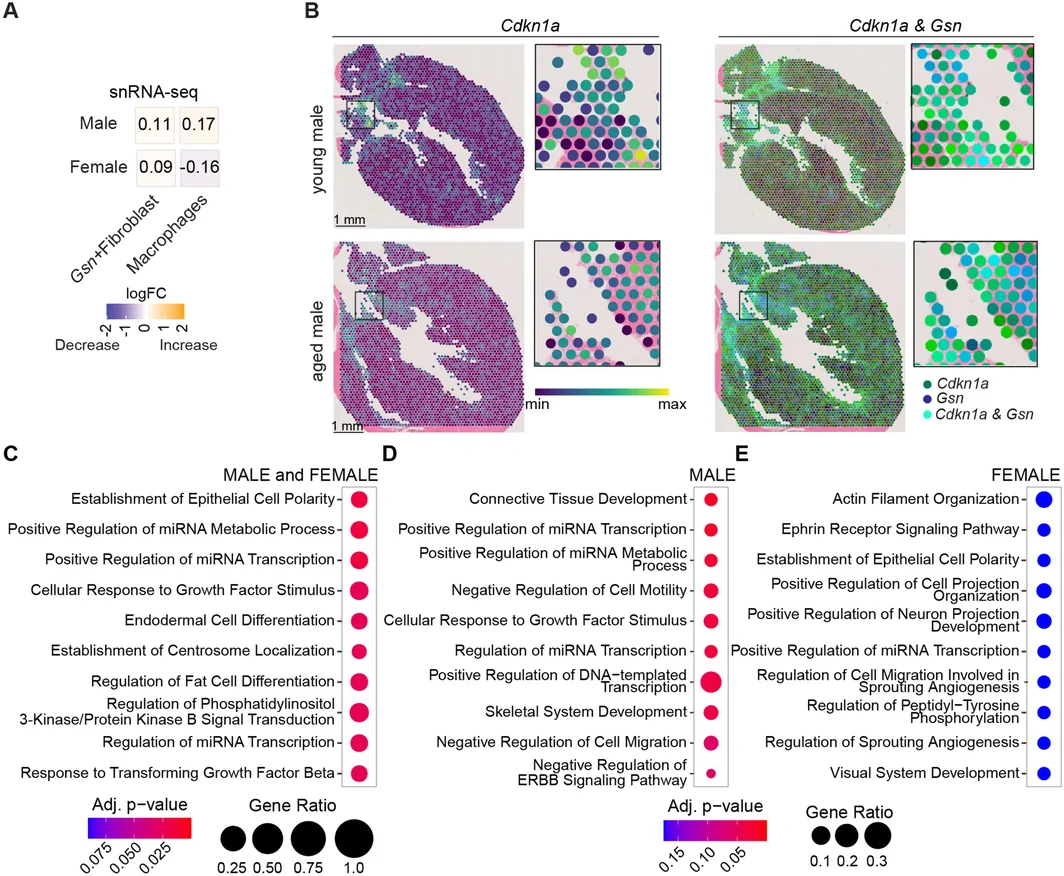
*Cdkn1a^+^* *Gsn^+^* fibroblast numbers increase with age and *Cdkn1a^+^* expression is predominant in valves. (A) Log ratio (aged/young) of the number of *Cdkn1a^+^* *Gsn^+^* fibroblasts and macrophages (*n* = 6 per sex); color scale (purple‐white‐orange)—log2FC. (B) Expression of *Cdkn1a* (green) and *Gsn* (dark blue) in mouse heart; cyan—co‐expression of *Cdkn1a* and *Gsn*; color scale (blue‐yellow)—normalized gene expression. (C, D, E) Dot plots show enriched GO BP (adj. *p*‐value < 0.05) based on significantly upregulated DEGs in *Cdkn1a^+^* *Gsn^+^* fibroblasts (*Cdkn1a^+^* vs. *Cdkn1a^—^*, *n* = 6 per sex, adj. *p*‐value < 0.05, log2FC > 0.36) in both sexes combined (C), males only (D), and females only (E). Dot color indicates the adjusted *p*‐value (red = more significant); dot size reflects the gene ratio (proportion of upregulated DEGs annotated to that term), per the scales shown below each panel with males and females only on the same scale.

Aged *Gsn* ^+^ fibroblasts showed enrichment of GO BP involved in ECM and collagen organization, together with angiogenesis regulation, VEGF signaling, and endothelial cell migration (Figure 3A), consistent with increased stromal involvement in age‐associated tissue remodeling. Notably, sex‐stratified GO BP analysis demonstrated that ECM organization was prominent in aged male fibroblasts, whereas aged female fibroblasts were characterized predominantly by translation and protein biosynthetic processes (Figure 3B,C).

Consistent with *Cdkn1a* ^+^ spatial expression, *Cdkn1a* ^+^ *Gsn* ^+^ fibroblasts were more abundant in aged hearts (Figure 5A). *Cdkn1a* was also co‐expressed with *Gsn* (fibroblast marker) in valves and aorta (Figure 5B). *Cdkn1a* ^+^ *Gsn* ^+^ fibroblasts displayed GO enrichment of biological processes associated to growth factor‐responsive signaling, including TGF‐β‐ and PI3K‐associated processes, and regulation of miRNA metabolism and transcription (Figures 5C and S8, Table S12). These findings suggest that *Cdkn1a* expression is associated with altered growth factor signaling and transcriptional regulation in fibroblasts. When analyzed per sex, significant enrichment in *Cdkn1a* ^+^ fibroblasts was restricted to males and included miRNA regulation, growth‐factor responses, and regulation of cell migration (Figure 5D,E). No significantly enriched GO BP were identified in females due to limited overlap of upregulated DEGs with existing terms.

### 3.5 *Cdkn1a* ^*+*^ Fibroblast‐Lineage Cell Programs Are Partially Conserved in Human Hearts

To assess the clinical relevance of mouse transcriptional profiles, we performed cross‐species alignment against a snRNA‐seq non‐failing human heart atlas comprising samples from various ages (Bleckwehl et al. 2025; Litvinukova et al. 2020). Transcripts in mouse cell types exhibited strong concordance with corresponding human cell populations, with 100% of mouse macrophages and 78.1% of mouse dendritic cells aligned with human myeloid populations whereas 76.2% of mouse *Gsn* ^+^ fibroblasts and 100% of mouse *Postn* ^+^ fibroblasts and 80.1% of *Dkk3* ^+^ fibroblasts aligned with human fibroblast population, consisting of nine fibroblast‐lineage subclusters including myofibroblast subpopulations (Figure 6A).

**FIGURE 6:**
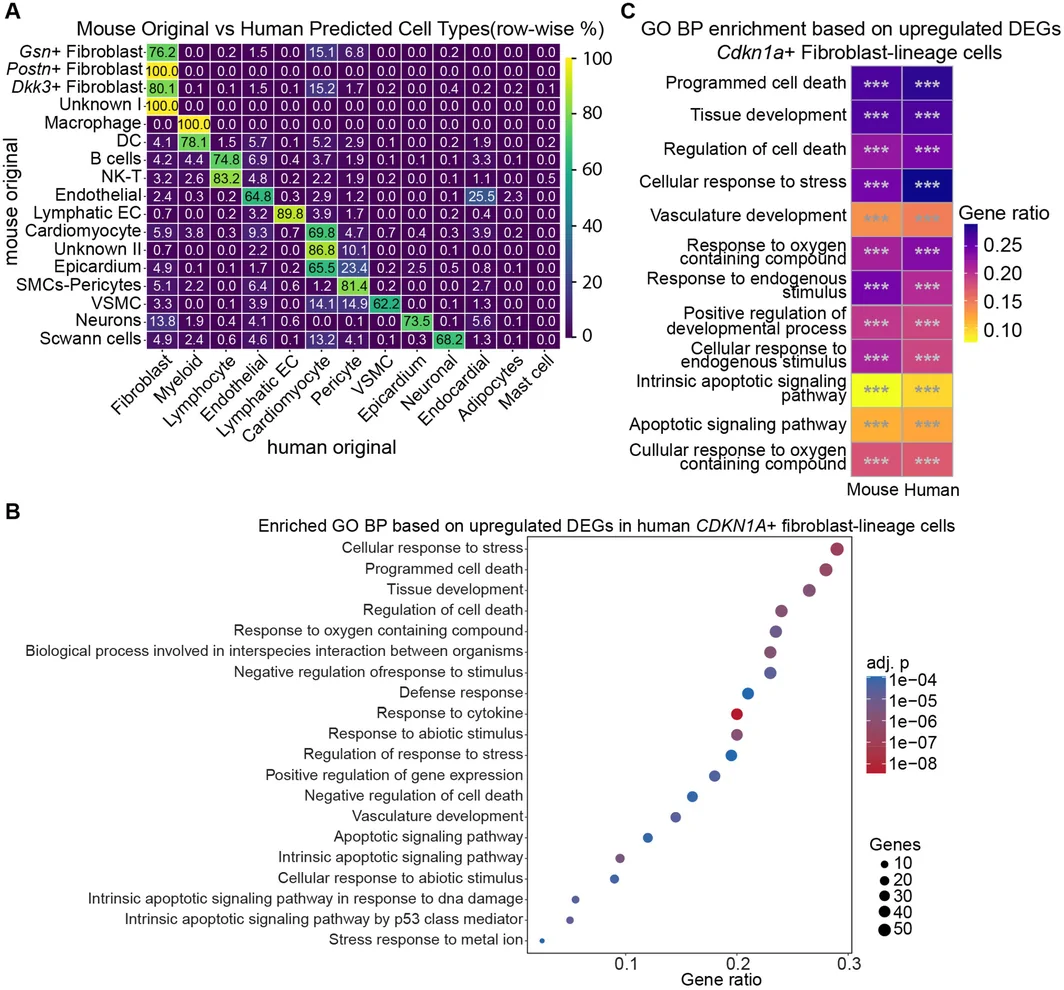
Cross‐species integration revealed conserved biological processes between human *CDKN1A* ^+^ and mouse *Cdkn1a* ^+^ fibroblast‐lineage cells. (A) Mouse/Human alignment map of cardiac cells (quantified using confusion matrix); color scale (blue‐yellow)—aligned percentage (%); human fibroblast population consisted of nine fibroblast‐lineage and two myofibroblast subpopulations. (B) Enriched GO BP (upregulated DEGs) in *CDKN1A* ^+^ human fibroblast‐lineage population. Dot size reflects gene count; color scale (blue to red)—adjusted *p*‐value. No term was significantly enriched among downregulated DEGs. (C) The 12 most significant of the 140 GO BP shared terms enriched among upregulated DEGs in human *CDKN1A* ^+^ and mouse *Cdkn1a* ^+^ fibroblast‐lineage populations (*Gsn* ^+^, *Dkk3* ^+^, and *Postn* ^+^ fibroblasts); (Benjamini–Hochberg adjusted *p* < 0.05 in each species; 381 terms enriched in mouse, 322 in human), ranked by combined significance across the two species; color scale (yellow to blue)—gene ratio; ***‐adjusted *p* < 0.001.

GO analysis of human *CDKN1A* ^+^ fibroblast‐lineage populations showed upregulation of cellular stress and cell death programs, including cellular response to stress, programmed cell death, and tissue development, alongside a strongly significant cytokine response process (Figure 6B), with no biological process significantly enriched among downregulated DEGs. Conservation of upregulated GO BP between mouse and human *Cdkn1a* ^+^ fibroblast‐lineage populations was partial: of 381 GO BP terms enriched in mouse and 322 in human, 140 were shared (36.7% and 43.5%, respectively), led by programmed cell death, regulation of cell death, cellular response to stress and tissue development (top 12 terms displayed, Figure 6C), accompanied by upregulation of p53 target genes in both species (*MDM2*, *ZMAT3* and *EDA2R*). Nonetheless, the majority of enriched processes was not shared between the two species. In summary, comparison of *CDKN1A*‐expressing human and mouse fibroblast‐lineage cells identified partial conservation of age‐related changes rather than a uniform cross‐species signature.

## 4 Discussion

In this study, we describe spatially and cellularly resolved signatures of macrophages and fibroblasts (key immune and stromal components of cardiac inflammaging) converging on the valve/aortic root from which inflammation‐ and senescence‐associated signals extend into the myocardium with age. Bulk RNA‐seq and WGCNA established inflammation and ECM remodeling as the dominant transcriptional signatures of the aging heart. Single‐nucleus profiling further defined macrophages as an expanding population and core driver of cardiac inflammaging, with *Gsn* ^+^ fibroblasts appearing to represent a precursor state along a continuum toward the *Postn* ^+^ phenotype rather than a fixed, discrete lineage. Spatial transcriptomic analysis provided evidence supporting that cardiac inflammaging is not a diffuse, heart‐wide process but one anchored in specific anatomical niches from which it progressively encroaches on the myocardium. These observations suggest that coordinated macrophage and fibroblast responses potentially extend the concept of an immune‐fibrotic interconnected network by placing it within a defined anatomical context.

Enriched GO BP findings point toward macrophage‐fibroblast interaction and mutual activation being central to age‐related remodeling. Aged macrophages upregulated growth‐factor and stromal signaling pathways (PDGF, VEGF, ERK1/2) alongside genes promoting fibroblast proliferation, while aged fibroblasts reciprocally upregulated ECM/collagen organization and angiogenesis programs. Analysis of *Cdkn1a* ^+^ *Gsn* ^+^ fibroblasts further identified a transcriptional profile enriched for TGF‐β‐ and PI3K‐associated processes. These complementary molecular programs are consistent with the idea of a macrophage‐fibroblast communication circuit promoting stromal remodeling in the aging heart, similar to reciprocal interactions previously described during cardiac fibrosis and pathological remodeling (Guarnieri and Turley 2026; Zhang et al. 2025).

The spatial data encompassing all four chambers, valves, and proximal great vessels provide this paper's most distinctive contribution to prior aging atlases (Schaum et al. 2020; Tabula Muris Consortium 2020; Vidal et al. 2019), anchoring cell populations anatomically and revealing enrichment of macrophages and *Gsn* ^+^ fibroblasts in valves and the aortic root. This extends previously described vascular/perivascular aging niches (Rodriguez Morales et al. 2025) to valvular and proximal aortic regions and is consistent with these regions serving as resident niches for immune and ECM‐remodeling activity during age‐related dysfunction (Mammoto et al. 2022; Scott et al. 2021). Aging did not amplify general macrophage and fibroblast markers, with *C1qa* and *Gsn* remaining spatially constant. Instead, aging was marked by region‐specific changes including an age‐related increase in *Cdkn1a* ^+^ *Gsn* ^+^ fibroblasts and *Cdkn1a* expression concentrating in the valve/aorta niche. Aortic enrichment of *Ccl8* added anatomical specificity to its previously reported association with profibrotic macrophage vascular niches in the aging heart of 18‐month‐old hearts, where it also drove age‐related enrichment of immune cell migration, granulocyte chemotaxis and ERK1/2 signaling consistent with our findings (Rodriguez Morales et al. 2025). Notably, both *Ccl8* and *Cdkn1a* became more prominent in the myocardium with age. The non‐uniform, regional expression of these senescence‐associated markers (Sanborn et al. 2025; Saul et al. 2022) suggests that senescence hallmarks (Gorgoulis et al. 2019) may represent localized, spatially defined changes rather than a diffuse, whole‐organ property. This distinction may be an important refinement to the conceptualization of cardiac inflammaging. Collectively, these observations extend previous studies, positing that inflammaging originates from senescence‐associated gene expression that accumulates preferentially in specific locations. Whether this focal expression reflects a locally contained process or contributes to broader systemic effects remains an open question.

Inclusion of both sexes at 24 months, a stage underrepresented in prior transcriptomic resources (Schaum et al. 2020; Tabula Muris Consortium 2020; Vidal et al. 2019), provided insight into sex‐dependent molecular features of advanced cardiac aging. Sex‐divergent patterns of aged fibroblasts from the entire heart revealed the engagement of specific molecular programs: males showed ECM/collagen and MAPK/Ras‐dominated signatures, including a male‐restricted *Cdkn1a* ^+^ fibroblast enrichment, while females were characterized more by cardiac morphogenesis and protein biosynthetic/translational processes. This is consistent with clinical observations that cardiac aging in men declines earlier and more linearly while women show later, accelerated post‐menopausal decline (Calvo‐Lopez et al. 2024), suggesting that these differing clinical trajectories may map onto distinct fibroblast transcriptional programs rather than a shared profile evolving at different rates.

Cross‐species comparison provided compelling evidence of strong alignment between mouse and human myeloid and fibroblast populations. Conservation of specific upregulated biological processes in *CDKN1A* ^+^ fibroblast‐lineage cells (notably cellular stress‐ and cell death‐ associated programs) supported the relevance of the mouse model to human cardiac aging. However, fewer than half of the enriched processes were shared between species, indicating that senescence‐associated transcriptional programs are only partially conserved, tempering the direct translation of mechanistic findings from mouse to human.

Fibroblasts and macrophages independently emerged as cellular ‘aging hubs’ in a large‐scale human plasma proteomic study, with fibroblast aging ranking among the strongest cell‐type‐specific predictors of incident heart failure (Ding et al. 2026). These convergent human proteomic findings corroborate orthogonal support, at the protein level and in an independent species, for a role of the macrophage‐fibroblast axis in cardiac aging identified transcriptionally in our aging mouse model. While the present study is limited to transcriptomic characterization, validation of proteins of interest is an important future direction. Currently, we are developing a cardiac panel based on Cell DIVE Imaging (Gerdes et al. 2013), a multiplex platform that simultaneously detects and localizes multiple proteins within whole heart tissue sections by antibody‐based detection, to examine presence of proteins of interest, such as p21 (Figure S9).

Together, these results support a model in which cardiac aging is amplified within a valve/aortic macrophage‐fibroblast niche, with senescence‐associated molecular features, including *Ccl8* and *Cdkn1a*, becoming progressively more prominent in the broader myocardium with age. These responses propose distinct sex‐specific molecular patterns and conservation of selected features in human tissue. The study reinforces the concept of inflammaging as a systemic, reciprocal process, refining where that process possibly initiates and how it changes anatomically, supporting a valve/aortic niche model rather than a uniform myocardial one. Moving beyond correlative evidence, determining whether this niche is a site of origin for inflammaging, rather than merely a site of detection, will require functional intervention, such as senolytic clearance of *Cdkn1a* ^+^ fibroblasts or blockade of *Ccl8*. Further studies will be needed to establish these cell types as causal drivers of age‐related cardiac dysfunction rather than markers of a broader aging process.

## Author Contributions

A.A.K., A.S., and Y.Z. contributed equally to this work. A.A.K. and A.S., interpreted results and wrote the paper. Y.Z. analyzed single‐nucleus sequencing data and led data/code deposition. A.S. analyzed spatial transcriptomics under supervision of B.S.W. H.C. analyzed bulk RNA‐seq. J.A.D., R.R., and G.T.C. prepared samples for analyses. J.A.D. and R.R. refined experimental designs. L.F. and S.H. analyzed human single‐nucleus sequencing data. J.M.M. shaped the paper structure and supervised the project. P.R., R.K., and N.A.R. conceptualized the project and acquired funding. All authors reviewed and gave final approval to the paper.

## Funding

This work was supported by The Jackson Laboratory Senescence Tissue Mapping Center (JAX‐Sen TMC U54 AG079753). We acknowledge assistance from the shared Scientific Services at The Jackson Laboratory, which were supported in part by the National Cancer Institute (NCI) grant (P30CA034196). G.T.C. was supported by JAX Scholar Award.

## Ethics Statement

All animal experiments were performed according to the National Institute of Health Guide for the Care and Use of Laboratory Animals (National Research Council) and were approved by The Jackson Laboratory's Animal Care Use Committee.

## Conflicts of Interest

The authors declare no conflicts of interest.

## Supporting information


**Figure S1:** Experimental design. Cardiac gene expression was compared between 6‐ and 24‐month‐old C57BL/6J mice (males and females, *n* = 3 per sex/age group) using four complementary transcriptomics approaches: bulk RNA‐seq (homogenization and mRNA extraction), single‐nucleus RNA‐seq (snRNA‐seq; homogenization and lysis), single‐cell RNA‐seq (scRNA‐seq; mechanical and enzymatic tissue dissociation), and spatial transcriptomics (Visium, 10× Genomics; FFPE tissue sections).
**Figure S2:** (A) Pseudo‐bulk principal component analysis (PCA) of bulk RNA‐seq data; each point represents one sample, colored by sex and age. (B) Volcano plot of cardiac DEGs based on bulk RNA‐seq between young (6 m) and aged (24 m) mice (males and females combined; *n* = 3 per sex/age); edgeR quasi‐likelihood F‐test (FDR < 0.05); red—upregulated in aged; blue—downregulated in aged; total = 287 DEGs.
**Figure S3:** Cell type annotation and marker gene expression for snRNA‐seq. (A) UMAP embedding of nuclei (snRNA‐seq; *n* = 116,002) colored by age: young (yellow, 6 m) and aged (blue, 24 m); males and females shown separately (*n* = 3 per sex/age); each point represents a single nucleus. (B) Dot plots showing expression of cell type marker genes used for cluster annotation in snRNA‐seq; dot size reflects percentage of nuclei expressing a given gene (annotations based on Forte et al. 2020); color scale (blue to red) reflects average normalized expression. (C) Pseudo‐bulk PCA of snRNA‐seq; each point represents one sample; filled circles—male; open circles—female; peach—aged; teal—young. (D) Stacked bar plots showing cell type proportions per sample for snRNA‐seq; samples ordered by age group (aged 24 m, young 6 m) and sex. (E) Pie charts of cell type proportions averaged per sex and age group for snRNA‐seq; young and aged shown separately for males (*n* = 3) and females (*n* = 3).
**Figure S4:** Expression of macrophage/myeloid marker genes across annotated cardiac cell types, stratified by age. Dot plot based on snRNA‐seq showing expression of canonical macrophage and monocyte‐lineage markers (*C1qa*, *C1qc*, *Cd68*, *Lyz2*, *Ctsb*, *Ccr2*, *Lyve1*, *Folr2*, *C3ar1*, *Cd74*, *Csf1r*, and *Ccl8*) across identified cardiac cell populations in young (6 months) and old (24 months) groups. Dot size represents the percentage of cells expressing each gene within a cluster, and color intensity represents scaled average expression per cell.
**Figure S5:** Fibroblast‐lineage cells form a transcriptional continuum, with aging shifting cells toward the *Postn^+^* fibroblast‐associated state in both sexes, without implying a uniform or irreversible transition. (A) Principal component analysis (PCA) of nuclei expressing *Gsn^+^* fibroblast (red) and *Postn^+^* fibroblasts (brown) from snRNA‐seq (both sexes, young and aged combined, *n* = 3 per age/sex); each point represents a single nucleus. (B) Pseudotime (PC1) values between *Gsn^+^* fibroblasts (red) and *Postn^+^* fibroblasts (green); Wilcoxon test, *p* < 2 × 10^−16^. (C) Rightward shift in cumulative pseudotime distribution (−log10 cumulative probability) in aged cells (red) relative to young (blue) (Kolmogorov–Smirnov test) (*Gsn^+^* and *Postn^+^* fibroblasts combined). (D) Mean pseudotime per individual in young (blue) versus aged (red) hearts (*n* = 6, two‐tailed Wilcoxon rank‐sum test, *p* = 0.39). (E) same as D per sex. (F) Heatmap of representative genes ordered along PC1 (pseudotime) using clustering by peak expression; expression is scaled per gene (*Z*‐score); each line ‐single nucleus; color scale from blue (low expression) to red (high expression).
Method for Pseudotime analysis:
*Gsn* ^+^ and *Postn* ^+^ fibroblasts were segregated from the full snRNA‐seq Seurat object, filtered, normalized (Seurat's NormalizeData) and scaled (ScaleData). Highly variable genes (VST method) were used to compute the PCA embedding (50 components) and visualize UMAP (the first five PCs). The preprocessed object was converted to a SingleCellExperiment object. Pseudotime inference was performed with Slingshot, using PCA coordinates as the reduced dimensional space, cluster labels as inputs, and *Gsn* ^+^ fibroblasts as the root state. As slingshot pseudotime was highly consistent with PC1, PC1 was used as surrogate for subsequent analysis.
All cells were assigned pseudotime/PC1 values. Spearman correlations between pseudotime and extracted scaled gene expression (from scale. data in Seurat object) were computed for all genes: the correlation coefficient and its associated *p*‐value were obtained (cor.test), followed by FDR correction. Genes (FDR < 0.05) were ranked by correlation strength (positively and negatively were ranked separately). A high‐confidence curated gene subset was assembled as following: nuclei were ordered by pseudotime; genes were ranked by their peak‐expression index (defined as the pseudotime position at which each gene's LOESS‐smoothed expression curve reached its maximum with capped expression values at ±2 prior to rendering, and disabling column clustering to preserve pseudotime ordering); GO BP enrichment was done using subsets of genes, where genes/cells ordered using both cells' position along the pseudotime/PC1 and gene's ranking. The curated gene subset was visualized on a heatmap, capturing heterogeneity and continuum of transcriptional dynamics. Pseudotime/PC1 distributions were compared between *Gsn* ^+^ fibroblasts and *Postn* ^+^ fibroblasts (Wilcoxon rank‐sum, *p* < 2 × 10^−16^, visualized using violin plots). Age effect was evaluated at the mouse level by computing mean pseudotime/PC1 per animal, with sex‐stratified analyses performed separately (Kolmogorov–Smirnov test for cumulative probability curves and two‐tailed Wilcoxon rank‐sum for boxplots). All visualizations were generated using ggplot2, ggpubr, pheatmap, and color‐blind‐safe palettes.
**Figure S6:** Permutation‐based significance of senescence‐associated gene expression in aortic spots. Heatmap showing FDR‐corrected *p*‐values from permutation testing of 17 published mouse heart‐specific senescence‐associated genes (Sanborn et al. 2025) across 12 Visium samples (aortic spots containing nuclei; *n* = 3 per sex/age group, except 2 aged females). Expression of each gene per sample was tested against a randomly generated null distribution. Each column represents one Visium sample; color scale (blue to red)—FDR‐corrected *p*‐value; sex and age annotations shown in top 2 rows: green‐ males, pink—females, purple—old; orange—young; gray—data from aorta of 2 aged females is not available because of the section orientation.
**Figure S7:** *Ccl8* expression and macrophage enrichment in aortic spots. Scatter plots showing normalized *Ccl8* expression (*x*‐axis) and inferred macrophage proportion (*y*‐axis) per spot for each aged male Visium sample (*n* = 3). Spots were classified as: *Ccl8*‐expressing with macrophage proportion > 0.05 (pink box); *Ccl8*‐expressing with macrophage proportion < 0.05; or spots without *Ccl8* expression and macrophage proportion > 0.05. Significance (TRUE, *p* < 0.05; FALSE) indicates spots in aorta.
**Figure S8:** Differential gene expression in *Cdkn1a^+^* fibroblast‐lineage cells. (A, B) Volcano plots showing DEGs between *Cdkn1a^+^* and *Cdkn1a^−^* *Gsn^+^* (A) and *Postn^+^* fibroblasts in snRNA‐seq data, stratified by sex (males and females; *n* = 6 per sex; young & aged combined); Wilcoxon rank‐sum test; adj. *p* value < 0.05; |log2FC| > 0.36; red—upregulated; blue—downregulated; gray—not significant (NS); *Cdkn1a^+/−^* cells defined as nuclei with non‐zero or zero normalized *Cdkn1a* expression, respectively.
**Figure S9:** Cell DIVE multiplex imaging of DAPI and p21 in a longitudinal mouse heart section. Preliminary raw data of (A) DAPI and (B) p21 (encoded by *Cdkn1a*) immunofluorescence staining across the entire longitudinal heart section. (C, D, G, H) Intermediate‐magnification views of the valve/aortic root (C, D) and myocardium (G, H) region selected from (A, B), respectively, as indicated by white boxes. (E, F) Higher‐magnification views of the aorta as indicated by white boxes in (C, D); color scale represents fluorescence intensity (arbitrary units) for each channel.

**Table S1:** Age‐related differentially expressed genes in bulk RNA‐seq; 6‐ versus 24‐month‐old C57BL/6J mice, *n* = 6 per group (*n* = 3 per sex; DEG analysis by age, both sexes combined), FDR < 0.05.
**Table S2:** Sex‐related differentially expressed genes in bulk RNA‐seq; 6‐ versus 24‐month‐old C57BL/6J mice, *n* = 3 per sex/age; FDR < 0.05.
**Table S3:** Differentially expressed genes in the significantly enriched KEGG pathways; aged mice; bulk RNA‐seq.
**Table S4:** List of all WGCNA modules that were tested in bulk RNA‐seq. Only 1 module in analysis by age had FDR < 0.05.
**Table S5:** List of genes in the significant age‐related WGCNA module (MEgreen module #31, genes from main Figure 1B,C), logFC and FDR from DEG analysis by age using bulk RNA‐seq (young vs. aged, *n* = 6; both sexes combined).
**Table S6:** Aging differentially expressed genes in macrophages; snRNA‐seq; 6‐ versus 24‐month‐old C57BL/6J mice, *n* = 3 per age/sex; adj. *p*‐value < 0.05, |log2FC| > 0.36.
**Table S7:** Top ten enriched Gene Ontology terms for Biological Process (GO BP) based on aged up‐ and down‐regulated DEGs in macrophages; snRNA‐seq, both sex combined, adj. *p*‐value < 0.05, |log2FC| > 0.36.
**Table S8:** Aging differentially expressed genes in *Gsn^+^* fibroblasts; snRNA‐seq; 6‐ versus 24‐month‐old C57BL/6J mice, *n* = 3 per age/sex; adj. *p*‐value < 0.05, |log2FC| > 0.36.
**Table S9:** Top ten enriched Gene Ontology terms for Biological Process (GO BP) based on age up‐ and down‐regulated DEGs in *Gsn^+^* fibroblasts; snRNA‐seq, both sex combined, adj. *p*‐value < 0.05, |log2FC| > 0.36.
**Table S10:** Aging differentially expressed genes in endothelial cells; snRNA‐seq; 6‐ versus 24‐month‐old C57BL/6J mice, *n* = 3 per age/sex; adj. *p*‐value < 0.05, |log2FC| > 0.36.
**Table S11:** Top ten enriched Gene Ontology terms for Biological Process (GO BP) based on age up‐ and down‐regulated DEGs in endothelial cells; snRNA‐seq, both sex combined, adj. *p*‐value < 0.05, |log2FC| > 0.36.
**Table S12:** Top ten enriched Gene Ontology terms for Biological Process (GO BP) based on age up‐ and down‐regulated DEGs in *Cdkn1a^+^* versus *Cdkn1a^−^ Gsn^+^* fibroblasts; snRNA‐seq, both age & sex combined, adj. *p*‐value < 0.05, |log2FC| > 0.36.

## Data Availability

The data that support the findings of this study are openly available in The Cellular Senescence Network (SenNet) Data Portal at https://doi.org/10.60586/SNT224.DNPS.747; https://github.com/TheJacksonLaboratory/HeartSeneDataAnalysis; https://jackson‐aging‐heart‐site.vercel.app/.
