# Supplementary material for: Alterations in the Immune‐Fibrotic Axis Are Spatially Localized in the Aging Heart
Source: Aging Cell. 2026 Nov 5;25(11):e70768. doi: 10.1111/acel.70768 (PMC13740023; doi:10.1111/acel.70768)
Supplement: Supplementary file 1 [file ACEL-25-e70768-s001.docx]

**Supplementary File 1 (Figures S1-9)**

**Alterations in the immune-fibrotic axis are spatially localized in the aging heart**

**Running title:** Localized Cell Signatures in Aging Mouse Hearts

Aleksandra Aljakna Khan^1,7^, Amalia Sintou^1,7^, Yue Zhao^2,7^, Antonios Somarakis^2^, Harshpreet Chandok^2^, Juliana Alcoforado Diniz^2^, Ramalakshmi Ramasamy^2^, Gretchen T. Clark^1^, Leon Frommer^3^, Sikander Hayat^3,4,5^, Brian S. White^2^, Paul Robson^2^, Ron Korstanje^1^, J. Matthew Mahoney^1,8^, Nadia A. Rosenthal^1,6,8^

^1^The Jackson Laboratory, Bar Harbor, ME, USA

^2^The Jackson Laboratory for Genomic Medicine, Farmington, CT, USA

^3^Department of Medicine 2, RWTH Aachen University, Medical Faculty, Aachen, Germany

^4^Cardiovascular Research Institute and Department of Medicine, Icahn School of Medicine at Mount Sinai, New York, NY, USA

^5^Windreich Department of Artificial Intelligence and Human Health, Icahn School of Medicine at Mount Sinai, New York, NY, USA

^6^National Heart and Lung Institute, Imperial College, London, UK.

^7^These authors contributed equally

^8^Co-corresponding authors

**Figure S1 | Experimental design.** Cardiac gene expression was compared between 6- and 24-month-old C57BL/6J mice (males and females, n=3 per sex/age group) using four complementary transcriptomics approaches: bulk RNA-seq (homogenization and mRNA extraction), single-nucleus RNA-seq (snRNA-seq; homogenization and lysis), single-cell RNA-seq (scRNA-seq; mechanical and enzymatic tissue dissociation), and spatial transcriptomics (Visium, 10x Genomics; FFPE tissue sections).

**Figure S2 |** (**A**) Pseudo-bulk principal component analysis (PCA) of bulk RNA-seq data; each point represents one sample, colored by sex and age. (**B**) Volcano plot of cardiac DEGs based on bulk RNA-seq between young (6m) and aged (24m) mice (males and females combined; n=3 per sex/age); edgeR quasi-likelihood F-test (FDR<0.05); red — upregulated in aged; blue — downregulated in aged; total = 287 DEGs.

**Figure S3 |** **Cell type annotation and marker gene expression for snRNA-seq.** (**A**) UMAP embedding of nuclei (snRNA-seq; n=116,002) colored by age: young (yellow, 6m) and aged (blue, 24m); males and females shown separately (n=3 per sex/age); each point represents a single nucleus. (**B**) Dot plots showing expression of cell type marker genes used for cluster annotation in snRNA-seq; dot size reflects percentage of nuclei expressing a given gene (annotations based on Forte et al., 2020); color scale (blue to red) reflects average normalized expression. (**C**) Pseudo-bulk PCA of snRNA-seq; each point represents one sample; filled circles — male; open circles — female; peach — aged; teal — young. (**D**) Stacked bar plots showing cell type proportions per sample for snRNA-seq; samples ordered by age group (aged 24m, young 6m) and sex. (**E**) Pie charts of cell type proportions averaged per sex and age group for snRNA-seq; young and aged shown separately for males (n=3) and females (n=3).

**Figure S4 |** **Expression of macrophage/myeloid marker genes across annotated cardiac cell types, stratified by age**. Dot plot based on snRNA-seq showing expression of canonical macrophage and monocyte-lineage markers (*C1qa, C1qc, Cd68, Lyz2, Ctsb, Ccr2, Lyve1, Folr2, C3ar1, Cd74, Csf1r,* and *Ccl8*) across identified cardiac cell populations in Young (6 months) and Old (24 months) groups. Dot size represents the percentage of cells expressing each gene within a cluster, and color intensity represents scaled average expression per cell.

**Figure S5 |** **Fibroblast-lineage cells** **form a transcriptional continuum, with aging shifting cells toward the *Postn^+^* fibroblast-associated state in both sexes, without implying a uniform or irreversible transition. (A)** Principal component analysis (PCA) of nuclei expressing *Gsn^+^* fibroblast (red) and *Postn^+^* fibroblasts (brown) from snRNA-seq (both sexes, young and aged combined, n=3 per age/sex); each point represents a single nucleus. **(B)** Pseudotime (PC1) values between *Gsn^+^* fibroblasts (red) and *Postn^+^* fibroblasts (green); Wilcoxon test, p < 2 × 10⁻¹⁶. **(C)** Rightward shift in cumulative pseudotime distribution (–log₁₀ cumulative probability) in aged cells (red) relative to young (blue) (Kolmogorov–Smirnov test) (*Gsn^+^* and *Postn^+^* fibroblasts combined). **(D)** Mean pseudotime per individual in young (blue) vs. aged (red) hearts (n=6, two-tailed Wilcoxon rank-sum test, p = 0.39). **(E)** same as D per sex. **(F)** Heatmap of representative genes ordered along PC1 (pseudotime) using clustering by peak expression; expression is scaled per gene (Z-score); each line -single nucleus; color scale from blue (low expression) to red (high expression)

**Method for Pseudotime analysis**

*Gsn^+^* and *Postn^+^* fibroblasts were segregated from the full snRNA-seq Seurat object, filtered, normalized (Seurat’s NormalizeData) and scaled (ScaleData). Highly variable genes (VST method) were used to compute the PCA embedding (50 components) and visualize UMAP (the first five PCs). The preprocessed object was converted to a SingleCellExperiment object. Pseudotime inference was performed with Slingshot, using PCA coordinates as the reduced dimensional space, cluster labels as inputs, and *Gsn^+^* fibroblasts as the root state. As slingshot pseudotime was highly consistent with PC1, PC1 was used as surrogate for subsequent analysis.

All cells were assigned pseudotime/PC1 values. Spearman correlations between pseudotime and extracted scaled gene expression (from scale.data in Seurat object) were computed for all genes: the correlation coefficient and its associated p-value were obtained (cor.test), followed by FDR correction. Genes (FDR < 0.05) were ranked by correlation strength (positively and negatively were ranked separately). A high-confidence curated gene subset was assembled as following: nuclei were ordered by pseudotime; genes were ranked by their peak-expression index (defined as the pseudotime position at which each gene's LOESS-smoothed expression curve reached its maximum with capped expression values at ±2 prior to rendering, and disabling column clustering to preserve pseudotime ordering); GO BP enrichment was done using subsets of genes, where genes/cells ordered using both cells’ position along the pseudotime/PC1 and gene’s ranking. The curated gene subset was visualized on a heatmap, capturing heterogeneity and continuum of transcriptional dynamics.

Pseudotime/PC1 distributions were compared between *Gsn^+^* fibroblasts and *Postn^+^* fibroblasts (Wilcoxon rank-sum, p < 2 × 10⁻¹⁶, visualized using violin plots). Age effect was evaluated at the mouse level by computing mean pseudotime/PC1 per animal, with sex-stratified analyses performed separately (Kolmogorov–Smirnov test for cumulative probability curves and two-tailed Wilcoxon rank-sum for boxplots). All visualizations were generated using ggplot2, ggpubr, pheatmap, and color-blind-safe palettes.

**Figure S6 |** **Permutation-based significance of senescence-associated gene expression in aortic spots**. Heatmap showing FDR-corrected p-values from permutation testing of 17 published mouse heart-specific senescence-associated genes (Sanborn et al., 2025) across 12 Visium samples (aortic spots containing nuclei; n=3 per sex/age group, except 2 aged females). Expression of each gene per sample was tested against a randomly generated null distribution. Each column represents one Visium sample; color scale (blue to red) – FDR-corrected p-value; sex and age annotations shown in top 2 rows: green- males, pink – females, purple – old; orange – young; grey – data from aorta of 2 aged females is not available because of the section orientation.

**Figure S7 |** ***Ccl8* expression and macrophage enrichment in aortic spots.** Scatter plots showing normalized *Ccl8* expression (x-axis) and inferred macrophage proportion (y-axis) per spot for each aged male Visium sample (n=3). Spots were classified as: *Ccl8*-expressing with macrophage proportion >0.05 (pink box); *Ccl8*-expressing with macrophage proportion <0.05; or spots without *Ccl8* expression and macrophage proportion >0.05. Significance (TRUE, p<0.05; FALSE) indicates spots in aorta.

**Figure S8** *|* **Differential gene expression in *Cdkn1a*+ fibroblast-lineage cells.** (**A, B**) Volcano plots showing DEGs between *Cdkn1a*^+^ and *Cdkn1a*^−^ *Gsn*^+^ (A) and *Postn^+^* fibroblasts in snRNA-seq data, stratified by sex (males and females; n=6 per sex; young & aged combined); Wilcoxon rank-sum test; adj.p.value<0.05; |log2FC| >0.36; red — upregulated; blue — downregulated; grey — not significant (NS); *Cdkn1a*+/− cells defined as nuclei with non-zero or zero normalized *Cdkn1a* expression, respectively.

**Figure S9** *|* **Cell DIVE multiplex imaging of DAPI and p21 in a longitudinal mouse heart section.** Preliminary raw data of **(A)** DAPI and **(B)** p21 (encoded by *Cdkn1a*) immunofluorescence staining across the entire longitudinal heart section. **(C, D, G, H)** Intermediate-magnification views of the valve/aortic root (C, D) and myocardium (G, H) region selected from (A, B), respectively, as indicated by white boxes. **(E, F)** Higher-magnification views of the aorta as indicated by white boxes in (C, D); color scale represents fluorescence intensity (arbitrary units) for each channel.
