# Supplementary material for: Alterations in the Immune‐Fibrotic Axis Are Spatially Localized in the Aging Heart
Source: Aging Cell. 2026 Nov 5;25(11):e70768. doi: 10.1111/acel.70768 (PMC13740023; doi:10.1111/acel.70768)
Supplement: Supplementary file 2 [file ACEL-25-e70768-s002.docx]

**Alterations in the immune-fibrotic axis are spatially localized in the aging heart**

Supplementary File 2 (Tables S1-12)

| **#** | **Name of Table**  **in Supplementary File 2** | **Analyses** |
| --- | --- | --- |
| 1 | Table S1 DEG by age | Bulk RNA-seq; DEG by AGE, [young vs aged, n=6, both sexes combined (n=3 per sex/age)]; |
| 2 | Table S2 DEG by sex | Bulk RNA-seq; DEG by SEX |
| 3 | Table S3 DEG in KEGG | List of differentially expressed genes mapping to enriched age-related KEGG pathways, bulk RNA-seq (Main text, Figure 1a) |
| 4 | Table S4 WGCNA | List of all WGCNA modules that were tested in bulk RNA Seq |
| 5 | Table S5 List of genes in green WGCNA | List of genes in the significant age-related WGCNA module (green module #31, genes from Figure 1d |
| 6 | Table S6 Macrophage DEG | snRNA-seq; young vs aged; both sexes, males, female |
| 7 | Table S7 Macrophage GO BP | Top 10 GO BP analysis using upregulated and downregulated DEGs from snRNA-seq |
| 8 | Table S8 *Gsn*+ Fibroblast DEG | snRNA-seq; young vs aged; both sexes, males, female |
| 9 | Table S9 *Gsn*+ Fibroblast GO BP | Top 10 GO BP analysis using upregulated and downregulated DEGs from snRNA-seq |
| 10 | Table S10 Endothelial Cells DEG | snRNA-seq; young vs aged; both sexes, males, female |
| 11 | Table S11 Endothelial Cells GO BP | Top 10 GO BP analysis using upregulated and downregulated DEGs from snRNA-seq |
| 12 | Table S12 *Cdkn1a*+ *Gsn*+ Fibroblast GO BP | Top 10 GO BP analysis: *Cdkn1a*+ vs *Cdkn1a-;* Young and aged combined, both sexes |

**Table S1**. Age-related differentially expressed genes in bulk RNA-seq; 6- vs 24-month-old C57BL/6J mice, n=6 per group (n=3 per sex; DEG analysis by age, both sexes combined), FDR<0.05.

| **Gene Name** | **logFC** | **FDR** | **Up/Down DEG** | **Name** |
| --- | --- | --- | --- | --- |
| *Dpp6* | 4.54 | 0.043 | up | dipeptidylpeptidase 6 |
| *Kcnk1* | 4.04 | 0.003 | up | potassium channel, subfamily K, member 1 |
| *Klk1b26* | 3.93 | 0.008 | up | kallikrein 1-related petidase b26 |
| *Cyp2b10* | 3.90 | 0.004 | up | cytochrome P450, family 2, subfamily b, polypeptide 10 |
| *C130026I21Rik* | 3.59 | 0.024 | up | NA |
| *Ccl8* | 3.26 | 0.01 | up | C-C motif chemokine ligand 8 |
| *Edn3* | 3.23 | 0.003 | up | endothelin 3 |
| *Pah* | 3.12 | 0.004 | up | phenylalanine hydroxylase |
| *AC147806.2* | 2.92 | 0.001 | up | No associated gene |
| *Serpina1b* | 2.86 | 0.003 | up | serine (or cysteine) preptidase inhibitor, clade A, member 1B |
| *Prom2* | 2.77 | 0.034 | up | prominin 2 |
| *Serpina1e* | 2.67 | 0.011 | up | serine (or cysteine) peptidase inhibitor, clade A, member 1E |
| *Lypd6* | 2.67 | 0.022 | up | LY6/PLAUR domain containing 6 |
| *AC168977.1* | 2.58 | 0.034 | up |  |
| *Csprs* | 2.51 | 0.003 | up | component of Sp100-rs |
| *Ppp2r2c* | 2.49 | 0.02 | up | protein phosphatase 2, regulatory subunit B, gamma |
| *Vgll2* | 2.44 | 0.02 | up | vestigial like family member 2 |
| *AC167036.1* | 2.43 | 0.017 | up |  |
| *Cdh22* | 2.33 | 0.003 | up | cadherin 22 |
| *Ryr1* | 2.22 | 0.016 | up | ryanodine receptor 1, skeletal muscle |
| *Gsta2* | 2.16 | 0.018 | up | glutathione S-transferase, alpha 2 (Yc2) |
| *Gm10719* | 2.11 | 0.043 | up |  |
| *Ano5* | 2.10 | 0.003 | up | anoctamin 5 |
| *Gm7609* | 2.04 | 0.003 | up | predicted pseudogene 7609 |
| *Cela1* | 2.01 | 0.036 | up | chymotrypsin-like elastase family, member 1 |
| *Cfb* | 1.99 | 0.039 | up | complement factor B |
| *Gm36161* | 1.98 | 0.003 | up | predicted gene, 36161 |
| *Dlgap1* | 1.71 | 0.029 | up | DLG associated protein 1 |
| *Nlrc3* | 1.70 | 0.046 | up | NLR family, CARD domain containing 3 |
| *Acsm5* | 1.70 | 0.003 | up | acyl-CoA synthetase medium-chain family member 5 |
| *Mpeg1* | 1.70 | 0.038 | up | macrophage expressed gene 1 |
| *Ctss* | 1.68 | 0.028 | up | cathepsin S |
| *Prkcq* | 1.64 | 0.024 | up | protein kinase C, theta |
| *Klra9* | 1.58 | 0.017 | up | killer cell lectin-like receptor subfamily A, member 9 |
| *H2-DMb1* | 1.57 | 0.029 | up | histocompatibility 2, class II, locus Mb1 |
| *Amy1* | 1.57 | 0.003 | up | amylase 1, salivary |
| *Trem2* | 1.56 | 0.008 | up | triggering receptor expressed on myeloid cells 2 |
| *Ly86* | 1.52 | 0.044 | up | lymphocyte antigen 86 |
| *Serpina3n* | 1.51 | 0.003 | up | serine (or cysteine) peptidase inhibitor, clade A, member 3N |
| *Apod* | 1.49 | 0.003 | up | apolipoprotein D |
| *Gas2l3* | 1.48 | 0.019 | up | growth arrest-specific 2 like 3 |
| *Shisa3* | 1.47 | 0.033 | up | shisa family member 3 |
| *Mmp3* | 1.42 | 0.022 | up | matrix metallopeptidase 3 |
| *Clec4n* | 1.41 | 0.048 | up | C-type lectin domain family 4, member n |
| *Irf5* | 1.40 | 0.044 | up | interferon regulatory factor 5 |
| *C4b* | 1.39 | 0.001 | up | complement C4B (Chido blood group) |
| *Capn3* | 1.38 | 0.018 | up | calpain 3 |
| *AC149090.1* | 1.31 | 0.017 | up |  |
| *Gm21188* | 1.31 | 0.035 | up | NA |
| *Irf7* | 1.27 | 0.008 | up | interferon regulatory factor 7 |
| *Cd209f* | 1.26 | 0.008 | up | CD209f antigen |
| *Ccr2* | 1.24 | 0.032 | up | C-C motif chemokine receptor 2 |
| *Fcna* | 1.23 | 0.042 | up | ficolin A |
| *Gpnmb* | 1.22 | 0.035 | up | glycoprotein (transmembrane) nmb |
| *Tdrp* | 1.21 | 0.022 | up | testis development related protein |
| *Cd209g* | 1.20 | 0.029 | up | CD209g antigen |
| *Ric3* | 1.20 | 0.003 | up | RIC3 acetylcholine receptor chaperone |
| *Naip5* | 1.19 | 0.044 | up | NLR family, apoptosis inhibitory protein 5 |
| *Fcgr1* | 1.17 | 0.008 | up | Fc receptor, IgG, high affinity I |
| *Cds1* | 1.16 | 0.003 | up | CDP-diacylglycerol synthase 1 |
| *Isg15* | 1.15 | 0.025 | up | ISG15 ubiquitin-like modifier |
| *Dio2* | 1.15 | 0.034 | up | deiodinase, iodothyronine, type II |
| *Ms4a7* | 1.15 | 0.033 | up | membrane-spanning 4-domains, subfamily A, member 7 |
| *Naip2* | 1.15 | 0.034 | up | NLR family, apoptosis inhibitory protein 2 |
| *Myot* | 1.11 | 0.036 | up | myotilin |
| *Ass1* | 1.10 | 0.029 | up | argininosuccinate synthetase 1 |
| *Ms4a6c* | 1.10 | 0.05 | up | membrane-spanning 4-domains, subfamily A, member 6C |
| *H2-T23* | 1.10 | 0.042 | up | histocompatibility 2, T region locus 23 |
| *Slamf9* | 1.09 | 0.022 | up | SLAM family member 9 |
| *Trpm6* | 1.09 | 0.047 | up | transient receptor potential cation channel, subfamily M, member 6 |
| *Lair1* | 1.08 | 0.044 | up | leukocyte-associated Ig-like receptor 1 |
| *Postn* | 1.07 | 0.003 | up | periostin, osteoblast specific factor |
| *Rmrp* | 1.07 | 0.034 | up | RNA component of mitochondrial RNAase P |
| *Cd68* | 1.06 | 0.022 | up | CD68 antigen |
| *Ackr4* | 1.04 | 0.041 | up | atypical chemokine receptor 4 |
| *Ms4a6d* | 1.04 | 0.024 | up | membrane-spanning 4-domains, subfamily A, member 6D |
| *Abat* | 1.03 | 0.034 | up | 4-aminobutyrate aminotransferase |
| *Ccl6* | 1.02 | 0.041 | up | C-C motif chemokine ligand 6 |
| *Skap2* | 1.02 | 0.005 | up | src family associated phosphoprotein 2 |
| *Clic1* | 1.02 | 0.048 | up | chloride intracellular channel 1 |
| *Tmem37* | 1.01 | 0.031 | up | transmembrane protein 37 |
| *Tyrobp* | 1.01 | 0.033 | up | TYRO protein tyrosine kinase binding protein |
| *Pld4* | 1.01 | 0.044 | up | phospholipase D family member 4 |
| *Aldh3b1* | 1.01 | 0.032 | up | aldehyde dehydrogenase 3 family, member B1 |
| *Trim30a* | 1.00 | 0.041 | up | tripartite motif-containing 30A |
| *Capg* | 0.99 | 0.008 | up | capping actin protein, gelsolin like |
| *B2m* | 0.99 | 0.017 | up | beta-2 microglobulin |
| *Plau* | 0.98 | 0.044 | up | plasminogen activator, urokinase |
| *Sncg* | 0.96 | 0.029 | up | synuclein, gamma |
| *S100a4* | 0.95 | 0.037 | up | S100 calcium binding protein A4 |
| *Lyz2* | 0.94 | 0.033 | up | lysozyme 2 |
| *Tmem176a* | 0.94 | 0.035 | up | transmembrane protein 176A |
| *Fxyd5* | 0.93 | 0.027 | up | FXYD domain-containing ion transport regulator 5 |
| *C1qa* | 0.92 | 0.008 | up | complement component 1, q subcomponent, alpha polypeptide |
| *Pamr1* | 0.91 | 0.04 | up | peptidase domain containing associated with muscle regeneration 1 |
| *C1qb* | 0.90 | 0.008 | up | complement component 1, q subcomponent, beta polypeptide |
| *Scn4b* | 0.90 | 0.018 | up | sodium channel, type IV, beta |
| *Srgn* | 0.88 | 0.049 | up | serglycin |
| *Neurl3* | 0.88 | 0.033 | up | neuralized E3 ubiquitin protein ligase 3 |
| *Ifit3b* | 0.88 | 0.015 | up | interferon-induced protein with tetratricopeptide repeats 3B |
| *Casp12* | 0.88 | 0.008 | up | caspase 12 |
| *Ifit1* | 0.87 | 0.044 | up | interferon-induced protein with tetratricopeptide repeats 1 |
| *Casp4* | 0.87 | 0.044 | up | caspase 4, apoptosis-related cysteine peptidase |
| *H2-K1* | 0.86 | 0.045 | up | histocompatibility 2, K1, K region |
| *Hmox1* | 0.86 | 0.029 | up | heme oxygenase 1 |
| *Pirb* | 0.86 | 0.047 | up | paired Ig-like receptor B |
| *Slc41a2* | 0.86 | 0.029 | up | solute carrier family 41, member 2 |
| *Icam1* | 0.85 | 0.047 | up | intercellular adhesion molecule 1 |
| *Bend6* | 0.85 | 0.044 | up | BEN domain containing 6 |
| *Wfdc17* | 0.85 | 0.021 | up | WAP four-disulfide core domain 17 |
| *Lrrcc1* | 0.84 | 0.004 | up | leucine rich repeat and coiled-coil domain containing 1 |
| *Crlf2* | 0.83 | 0.039 | up | cytokine receptor-like factor 2 |
| *Tmem150c* | 0.83 | 0.014 | up | transmembrane protein 150C |
| *H2-D1* | 0.83 | 0.017 | up | histocompatibility 2, D region locus 1 |
| *H2-T22* | 0.82 | 0.033 | up | histocompatibility 2, T region locus 22 |
| *Cilp* | 0.82 | 0.039 | up | cartilage intermediate layer protein, nucleotide pyrophosphohydrolase |
| *Ifi204* | 0.81 | 0.008 | up | interferon activated gene 204 |
| *Oasl2* | 0.80 | 0.048 | up | 2'-5' oligoadenylate synthetase-like 2 |
| *BC028528* | 0.80 | 0.043 | up | cDNA sequence BC028528 |
| *AW112010* | 0.80 | 0.025 | up | expressed sequence AW112010 |
| *Bnc2* | 0.79 | 0.007 | up | basonuclin zinc finger protein 2 |
| *C1qc* | 0.79 | 0.017 | up | complement component 1, q subcomponent, C chain |
| *Gimap4* | 0.77 | 0.034 | up | GTPase, IMAP family member 4 |
| *Gngt2* | 0.75 | 0.041 | up | guanine nucleotide binding protein (G protein), gamma transducing activity polypeptide 2 |
| *Anxa2* | 0.74 | 0.041 | up | annexin A2 |
| *Fcgr3* | 0.73 | 0.017 | up | Fc receptor, IgG, low affinity III |
| *H2-Q4* | 0.73 | 0.038 | up | histocompatibility 2, Q region locus 4 |
| *Ifit3* | 0.72 | 0.039 | up | interferon-induced protein with tetratricopeptide repeats 3 |
| *Lgals3bp* | 0.71 | 0.046 | up | lectin, galactoside-binding, soluble, 3 binding protein |
| *Shtn1* | 0.71 | 0.03 | up | shootin 1 |
| *Ftl1* | 0.70 | 0.034 | up | ferritin light polypeptide 1 |
| *S100a11* | 0.69 | 0.044 | up | S100 calcium binding protein A11 |
| *Ifitm3* | 0.69 | 0.01 | up | interferon induced transmembrane protein 3 |
| *St8sia6* | 0.69 | 0.017 | up | ST8 alpha-N-acetyl-neuraminide alpha-2,8-sialyltransferase 6 |
| *Myl12b* | 0.68 | 0.04 | up | myosin, light chain 12B, regulatory |
| *Crip1* | 0.68 | 0.01 | up | cysteine-rich protein 1 |
| *Ly6a* | 0.68 | 0.004 | up | lymphocyte antigen 6 family member A |
| *Ociad2* | 0.67 | 0.038 | up | OCIA domain containing 2 |
| *Emp3* | 0.66 | 0.034 | up | epithelial membrane protein 3 |
| *S100a10* | 0.65 | 0.044 | up | S100 calcium binding protein A10 (calpactin) |
| *Pltp* | 0.64 | 0.039 | up | phospholipid transfer protein |
| *Cx3cl1* | 0.64 | 0.041 | up | C-X3-C motif chemokine ligand 1 |
| *Pla2g4a* | 0.63 | 0.049 | up | phospholipase A2, group IVA (cytosolic, calcium-dependent) |
| *Ces2g* | 0.63 | 0.039 | up | carboxylesterase 2G |
| *Il33* | 0.63 | 0.044 | up | interleukin 33 |
| *Fcgr2b* | 0.62 | 0.022 | up | Fc receptor, IgG, low affinity IIb |
| *Cpne8* | 0.62 | 0.021 | up | copine VIII |
| *Anxa1* | 0.62 | 0.02 | up | annexin A1 |
| *Icam2* | 0.60 | 0.034 | up | intercellular adhesion molecule 2 |
| *Cyb5a* | 0.58 | 0.042 | up | cytochrome b5 type A (microsomal) |
| *Pnp* | 0.57 | 0.048 | up | purine-nucleoside phosphorylase |
| *Maoa* | 0.56 | 0.034 | up | monoamine oxidase A |
| *Cd38* | 0.55 | 0.042 | up | CD38 antigen |
| *Cxcl16* | 0.54 | 0.048 | up | C-X-C motif chemokine ligand 16 |
| *Tagln2* | 0.53 | 0.034 | up | transgelin 2 |
| *Cd302* | 0.52 | 0.044 | up | CD302 antigen |
| *Rai14* | 0.52 | 0.019 | up | retinoic acid induced 14 |
| *Nedd9* | 0.51 | 0.044 | up | neural precursor cell expressed, developmentally down-regulated gene 9 |
| *F13a1* | 0.49 | 0.01 | up | coagulation factor XIII, A1 subunit |
| *Rnf115* | 0.48 | 0.018 | up | ring finger protein 115 |
| *Gbp2* | 0.47 | 0.021 | up | guanylate binding protein 2 |
| *C1s1* | 0.46 | 0.033 | up | complement component 1, s subcomponent 1 |
| *Ctsh* | 0.45 | 0.026 | up | cathepsin H |
| *Gm13212* | 0.45 | 0.042 | up | NA |
| *Ephx1* | 0.45 | 0.032 | up | epoxide hydrolase 1, microsomal |
| *Gbp6* | 0.44 | 0.034 | up | guanylate binding protein 6 |
| *Sh3bgrl* | 0.44 | 0.045 | up | SH3-binding domain glutamic acid-rich protein like |
| *Anxa4* | 0.42 | 0.043 | up | annexin A4 |
| *Mindy3* | 0.42 | 0.049 | up | MINDY lysine 48 deubiquitinase 3 |
| *C1ra* | 0.41 | 0.044 | up | complement component 1, r subcomponent A |
| *Atp6v0e* | 0.41 | 0.044 | up | ATPase, H+ transporting, lysosomal V0 subunit E |
| *S100a13* | 0.41 | 0.044 | up | S100 calcium binding protein A13 |
| *Vim* | 0.41 | 0.043 | up | vimentin |
| *Plbd1* | 0.40 | 0.044 | up | phospholipase B domain containing 1 |
| *Fuca1* | 0.39 | 0.036 | up | fucosidase, alpha-L- 1, tissue |
| *Tmem50a* | 0.38 | 0.035 | up | transmembrane protein 50A |
| *Ostm1* | 0.38 | 0.044 | up | osteopetrosis associated transmembrane protein 1 |
| *Csf1r* | 0.37 | 0.048 | up | colony stimulating factor 1 receptor |
| *Entpd1* | 0.36 | 0.041 | up | ectonucleoside triphosphate diphosphohydrolase 1 |
| *Klhl5* | 0.36 | 0.044 | up | kelch-like 5 |
| *Sptlc2* | 0.30 | 0.048 | up | serine palmitoyltransferase, long chain base subunit 2 |
| *Sri* | 0.29 | 0.041 | up | sorcin |
| *Arf4* | 0.28 | 0.045 | up | ARF GTPase 4 |
| *Isoc1* | -0.27 | 0.042 | down | isochorismatase domain containing 1 |
| *Rnf216* | -0.29 | 0.044 | down | ring finger protein 216 |
| *Pex6* | -0.29 | 0.048 | down | peroxisomal biogenesis factor 6 |
| *Extl3* | -0.30 | 0.048 | down | exostosin-like glycosyltransferase 3 |
| *Las1l* | -0.33 | 0.044 | down | LAS1-like (S. cerevisiae) |
| *Col1a2* | -0.35 | 0.044 | down | collagen, type I, alpha 2 |
| *Gtf3c1* | -0.36 | 0.041 | down | general transcription factor III C 1 |
| *Col6a2* | -0.38 | 0.045 | down | collagen, type VI, alpha 2 |
| *Idua* | -0.39 | 0.039 | down | iduronidase, alpha-L |
| *Lmtk2* | -0.40 | 0.023 | down | lemur tyrosine kinase 2 |
| *Nbas* | -0.40 | 0.039 | down | neuroblastoma amplified sequence |
| *Pank1* | -0.40 | 0.044 | down | pantothenate kinase 1 |
| *Emc1* | -0.40 | 0.041 | down | ER membrane protein complex subunit 1 |
| *Cln6* | -0.41 | 0.042 | down | ceroid-lipofuscinosis, neuronal 6 |
| *Gdpd5* | -0.42 | 0.049 | down | glycerophosphodiester phosphodiesterase domain containing 5 |
| *Frmd4a* | -0.44 | 0.043 | down | FERM domain containing 4A |
| *Myo18a* | -0.45 | 0.045 | down | myosin XVIIIA |
| *Adhfe1* | -0.45 | 0.041 | down | alcohol dehydrogenase, iron containing, 1 |
| *Ralgapa1* | -0.45 | 0.034 | down | Ral GTPase activating protein, alpha subunit 1 |
| *Mapk8ip3* | -0.46 | 0.02 | down | mitogen-activated protein kinase 8 interacting protein 3 |
| *Ift172* | -0.46 | 0.044 | down | intraflagellar transport 172 |
| *Polr1a* | -0.47 | 0.044 | down | polymerase (RNA) I polypeptide A |
| *Cluh* | -0.47 | 0.044 | down | clustered mitochondria homolog |
| *Mast4* | -0.48 | 0.048 | down | microtubule associated serine/threonine kinase family member 4 |
| *Tgfbr3* | -0.49 | 0.04 | down | transforming growth factor, beta receptor III |
| *Ppm1e* | -0.49 | 0.044 | down | protein phosphatase 1E (PP2C domain containing) |
| *Map3k5* | -0.51 | 0.034 | down | mitogen-activated protein kinase kinase kinase 5 |
| *Glg1* | -0.51 | 0.038 | down | golgi apparatus protein 1 |
| *Vps13d* | -0.52 | 0.043 | down | vacuolar protein sorting 13D |
| *Rbpms2* | -0.53 | 0.048 | down | RNA binding protein with multiple splicing 2 |
| *Col1a1* | -0.54 | 0.036 | down | collagen, type I, alpha 1 |
| *Phlpp1* | -0.55 | 0.049 | down | PH domain and leucine rich repeat protein phosphatase 1 |
| *Plce1* | -0.55 | 0.044 | down | phospholipase C, epsilon 1 |
| *Col5a3* | -0.57 | 0.038 | down | collagen, type V, alpha 3 |
| *Osbpl3* | -0.57 | 0.031 | down | oxysterol binding protein-like 3 |
| *Ntn1* | -0.58 | 0.049 | down | netrin 1 |
| *Nhsl1* | -0.58 | 0.034 | down | NHS like 1 |
| *Armcx3* | -0.58 | 0.011 | down | armadillo repeat containing, X-linked 3 |
| *Lrpprc* | -0.59 | 0.043 | down | leucine-rich PPR-motif containing |
| *Kcnb1* | -0.59 | 0.018 | down | potassium voltage gated channel, Shab-related subfamily, member 1 |
| *Acacb* | -0.60 | 0.024 | down | acetyl-Coenzyme A carboxylase beta |
| *Tmem94* | -0.60 | 0.024 | down | transmembrane protein 94 |
| *Rnf150* | -0.60 | 0.041 | down | ring finger protein 150 |
| *Lama2* | -0.61 | 0.049 | down | laminin, alpha 2 |
| *Atp11a* | -0.61 | 0.041 | down | ATPase, class VI, type 11A |
| *Ppfibp2* | -0.62 | 0.035 | down | PTPRF interacting protein, binding protein 2 (liprin beta 2) |
| *Ppl* | -0.63 | 0.047 | down | periplakin |
| *Parm1* | -0.63 | 0.048 | down | prostate androgen-regulated mucin-like protein 1 |
| *Obsl1* | -0.64 | 0.042 | down | obscurin-like 1 |
| *Pdzrn3* | -0.65 | 0.035 | down | PDZ domain containing RING finger 3 |
| *Tceal8* | -0.65 | 0.033 | down | transcription elongation factor A (SII)-like 8 |
| *Igf1r* | -0.66 | 0.029 | down | insulin-like growth factor I receptor |
| *Col6a3* | -0.66 | 0.029 | down | collagen, type VI, alpha 3 |
| *Dmxl2* | -0.67 | 0.045 | down | Dmx-like 2 |
| *Lamb2* | -0.67 | 0.044 | down | laminin, beta 2 |
| *Tbc1d4* | -0.68 | 0.03 | down | TBC1 domain family, member 4 |
| *Adcy5* | -0.69 | 0.034 | down | adenylate cyclase 5 |
| *Sox6* | -0.69 | 0.044 | down | SRY (sex determining region Y)-box 6 |
| *Neo1* | -0.70 | 0.041 | down | neogenin |
| *Irs1* | -0.70 | 0.034 | down | insulin receptor substrate 1 |
| *Nav2* | -0.73 | 0.043 | down | neuron navigator 2 |
| *Lingo3* | -0.73 | 0.044 | down | leucine rich repeat and Ig domain containing 3 |
| *Plxnb1* | -0.73 | 0.034 | down | plexin B1 |
| *Ltbp1* | -0.73 | 0.041 | down | latent transforming growth factor beta binding protein 1 |
| *Pcdh7* | -0.74 | 0.036 | down | protocadherin 7 |
| *Hspg2* | -0.74 | 0.048 | down | perlecan (heparan sulfate proteoglycan 2) |
| *Slc7a1* | -0.74 | 0.039 | down | solute carrier family 7 (cationic amino acid transporter, y+ system), member 1 |
| *Ppp1r9a* | -0.75 | 0.04 | down | protein phosphatase 1, regulatory subunit 9A |
| *Carns1* | -0.75 | 0.02 | down | carnosine synthase 1 |
| *Ank2* | -0.76 | 0.044 | down | ankyrin 2, brain |
| *Ppargc1b* | -0.76 | 0.021 | down | peroxisome proliferative activated receptor, gamma, coactivator 1 beta |
| *Kcnd2* | -0.76 | 0.044 | down | potassium voltage-gated channel, Shal-related family, member 2 |
| *Plec* | -0.76 | 0.046 | down | plectin |
| *Celsr2* | -0.77 | 0.044 | down | cadherin, EGF LAG seven-pass G-type receptor 2 |
| *Syde2* | -0.79 | 0.039 | down | synapse defective 1, Rho GTPase, homolog 2 (C. elegans) |
| *Myo18b* | -0.79 | 0.039 | down | myosin XVIIIb |
| *Sned1* | -0.80 | 0.016 | down | sushi, nidogen and EGF-like domains 1 |
| *Aff2* | -0.82 | 0.047 | down | AF4/FMR2 family, member 2 |
| *Aqp4* | -0.82 | 0.02 | down | aquaporin 4 |
| *Zfp879* | -0.83 | 0.04 | down | zinc finger protein 879 |
| *Fzd3* | -0.83 | 0.041 | down | frizzled class receptor 3 |
| *Frem2* | -0.85 | 0.034 | down | Fras1 related extracellular matrix protein 2 |
| *Lgr6* | -0.89 | 0.032 | down | leucine-rich repeat-containing G protein-coupled receptor 6 |
| *Hmcn1* | -0.89 | 0.031 | down | hemicentin 1 |
| *Slc38a4* | -0.96 | 0.017 | down | solute carrier family 38, member 4 |
| *Nckap5* | -0.98 | 0.022 | down | NCK-associated protein 5 |
| *Flvcr2* | -0.99 | 0.044 | down | feline leukemia virus subgroup C cellular receptor 2 |
| *Tmem35a* | -0.99 | 0.044 | down | transmembrane protein 35A |
| *Sorcs2* | -1.00 | 0.03 | down | sortilin-related VPS10 domain containing receptor 2 |
| *Kcna6* | -1.00 | 0.036 | down | potassium voltage-gated channel, shaker-related, subfamily, member 6 |
| *Obscn* | -1.03 | 0.041 | down | obscurin, cytoskeletal calmodulin and titin-interacting RhoGEF |
| *Gal3st2c* | -1.03 | 0.008 | down | galactose-3-O-sulfotransferase 2C |
| *Rnf207* | -1.03 | 0.029 | down | ring finger protein 207 |
| *Peg3* | -1.04 | 0.022 | down | paternally expressed 3 |
| *Myh6* | -1.07 | 0.034 | down | myosin, heavy polypeptide 6, cardiac muscle, alpha |
| *Col6a6* | -1.14 | 0.042 | down | collagen, type VI, alpha 6 |
| *Pde1c* | -1.16 | 0.033 | down | phosphodiesterase 1C |
| *Kcna1* | -1.17 | 0.027 | down | potassium voltage-gated channel, shaker-related subfamily, member 1 |
| *Kcne1* | -1.23 | 0.044 | down | potassium voltage-gated channel, Isk-related subfamily, member 1 |
| *Plekhh1* | -1.23 | 0.046 | down | pleckstrin homology domain containing, family H (with MyTH4 domain) member 1 |
| *Armcx4* | -1.26 | 0.044 | down | armadillo repeat containing, X-linked 4 |
| *Nrk* | -1.37 | 0.005 | down | Nik related kinase |
| *Cdh20* | -1.54 | 0.038 | down | cadherin 20 |
| *Ddc* | -2.36 | 0.004 | down | dopa decarboxylase |

**Table S2**. Sex-related differentially expressed genes in bulk RNA-seq; 6- vs 24-month-old C57BL/6J mice, n=3 per sex/age; FDR<0.05

| **Gene Name** | **logFC** | **FDR** | **direction** | **Name** |
| --- | --- | --- | --- | --- |
| Ddx3y | 15.0 | 1.25E-07 | up | DEAD box helicase 3, Y-linked |
| Eif2s3y | 13.7 | 5.64E-07 | up | eukaryotic translation initiation factor 2, subunit 3, structural gene Y-linked |
| Uty | 6.7 | 2.94E-09 | up | ubiquitously transcribed tetratricopeptide repeat containing, Y-linked |
| Kdm5d | 5.8 | 7.92E-08 | up | lysine demethylase 5D |
| C7 | 3.0 | 7.23E-05 | up | complement component 7 |
| Cnmd | 1.8 | 2.74E-02 | up | chondromodulin |
| Lvrn | 1.7 | 6.87E-03 | up | laeverin |
| Slc16a6 | 0.8 | 8.52E-03 | up | solute carrier family 16 (monocarboxylic acid transporters), member 6 |
| Eif2s3x | -0.6 | 1.46E-03 | down | eukaryotic translation initiation factor 2, subunit 3, structural gene X-linked |
| Lepr | -1.0 | 6.87E-03 | down | leptin receptor |
| Kdm6a | -1.1 | 2.79E-02 | down | lysine (K)-specific demethylase 6A |
| Xist | -12.9 | 1.23E-08 | down | inactive X specific transcripts |

**Table S3**. Differentially expressed genes in the significantly enriched KEGG pathways; aged mice; bulk RNA-seq

| **Enriched Pathway** | **Genes mapping to the pathway** |
| --- | --- |
|  | **(red - upregulated, blue - downregulated)** |
| Complement and coagulation cascades | *C1qa, C1qb, C1qc, C1ra, C1s1, C4b, Cfb, F13a1, Plau, Serpina1b, Serpina1e* |
| Staphylococcus aureus infection | *C1qa, C1qb, C1qc, C1ra, C1s1, C4b, Cfb, Fcgr1, Fcgr2b, Fcgr3, H2-DMb1, Icam1* |
| Phagosome | *C1ra, Fcgr1, Fcgr2b, Fcgr3, Cd209f, Cd209g, H2-D1, H2-DMb1, H2-K1, H2-Q4, H2-T22, H2-T23, Ctss, Atp6v0e* |
| Cytoskeleton in muscle cells | *Vim, Capn3, Myot, Col1a1, Col1a2, Col5a3, Col6a2, Col6a3, Col6a6, Myh6, Lama2, Plec, Hspg2, Obscn, Ank2* |
| ECM-receptor interaction | *Col1a1, Col1a2, Col6a2, Col6a3, Col6a6, Lama2, Lamb2, Hspg2, Frem2* |
| Viral myocarditis | *H2-D1, H2-DMb1, H2-K1, H2-Q4, H2-T22, H2-T23, Icam1, Lama2, Myh6* |
| Human papillomavirus infection | *H2-D1, H2-K1, H2-Q4, H2-T22, H2-T23, Isg15, Atp6v0e, Oasl2, Ppp2r2c, Col1a1, Col1a2, Col6a2, Col6a3, Col6a6, Lama2, Lamb2, Fzd3* |
| Antigen processing and presentation | *B2m, Ctss, H2-D1, H2-DMb1, H2-K1, H2-Q4, H2-T22,H2-T23* |
| Epstein-Barr virus infection | *Vim, Icam1, B2m, H2-D1, H2-DMb1, H2-K1, H2-Q4, H2-T22, H2-T23, Isg15, Irf7, Entpd1* |
| Phenylalanine metabolism | *Aldh3b1, Maoa, Pah, Ddc* |
| Allograft rejection | *H2-D1, H2-DMb1, H2-K1, H2-Q4, H2-T22, H2-T23* |
| Graft-versus-host disease | *H2-D1, H2-DMb1, H2-K1, H2-Q4, H2-T22, H2-T23* |
| Cell adhesion molecules | *H2-D1, H2-DMb1, H2-K1, H2-Q4, H2-T22, H2-T23, Icam1, Icam2, Glg1, Neo1* |
| Systemic lupus erythematosus | *C1qa, C1qb, C1qc, C1ra, C1s1, C4b, Fcgr1, Fcgr3, H2-DMb1* |
| Type I diabetes mellitus | *H2-D1, H2-DMb1, H2-K1, H2-Q4, H2-T22, H2-T23* |
| Autoimmune thyroid disease | *H2-D1, H2-DMb1, H2-K1, H2-Q4, H2-T22, H2-T23* |
| Pertussis | *C1qa, C1qb, C1qc, C1ra, C1s1, C4b* |
| Virion-Ebolavirus, Lyssavirus and Morbillivirus | *Cd209f, Cd209g, Fcgr2b* |
| Natural killer cell mediated cytotoxicity | *H2-D1, H2-K1, H2-T23, Icam1, Icam2, Tyrobp, Klra9* |
| Viral protein interaction with cytokine and cytokine receptor | *Ackr4, Ccl6, Ccl8, Ccr2, Csf1r, Cx3cl1* |
| PI3K-Akt signaling pathway | *Col1a1, Col1a2, Col6a2, Col6a3, Col6a6, Lama2, Lamb2, Igf1r, Irs1, Phlpp1, Csf1r, Gngt2, Ppp2r2c* |
| Focal adhesion | *Col1a1, Col1a2, Col6a2, Col6a3, Col6a6, Lama2, Lamb2, Igf1r, Myl12b* |
| Virion-Lassa virus and SFTS virus | *Cd209f, Cd209g, Pcdh7* |
| Osteoclast differentiation | *Csf1r, Trem2, Fcgr1, Fcgr2b, Fcgr3, Tyrobp, Pirb* |
| Protein digestion and absorption | *Col1a1, Col1a2, Col5a3, Col6a2, Col6a3, Col6a6* |

**Table S4**. List of all WGCNA modules that were tested in bulk RNA Seq. Only 1 module in analysis by **age** had FDR<0.05

| **Module #** | **Module Name** | **FDR_age** | **FDR_sex** | **FDR_interaction** |
| --- | --- | --- | --- | --- |
| **31** | **MEgreen** | **0.000** | 0.193 | 0.487 |
| 12 | MElightgreen | 0.051 | 0.134 | 0.487 |
| 10 | MEblue | 0.239 | 0.985 | 0.623 |
| 3 | MEyellow | 0.278 | 0.985 | 0.878 |
| 28 | MEblack | 0.339 | 0.471 | 0.623 |
| 11 | MEroyalblue | 0.434 | 0.985 | 0.878 |
| 14 | MEorange | 0.434 | 0.193 | 0.487 |
| 19 | MEturquoise | 0.434 | 0.985 | 0.623 |
| 23 | MEmagenta | 0.434 | 0.985 | 0.623 |
| 29 | MEcyan | 0.434 | 0.985 | 0.623 |
| 1 | MEgrey60 | 0.505 | 0.462 | 0.623 |
| 2 | MEgreenyellow | 0.505 | 0.462 | 0.623 |
| 4 | MEdarkturquoise | 0.505 | 0.462 | 0.623 |
| 5 | MEpink | 0.505 | 0.462 | 0.623 |
| 15 | MEpaleturquoise | 0.505 | 0.485 | 0.487 |
| 25 | **MEsaddlebrown** | 0.505 | **0.002** | 0.878 |
| 30 | MEdarkgrey | 0.505 | 0.936 | 0.781 |
| 17 | MEskyblue | 0.561 | 0.985 | 0.878 |
| 13 | MElightyellow | 0.566 | 0.512 | 0.623 |
| 18 | MEdarkgreen | 0.599 | 0.730 | 0.487 |
| 32 | MEgrey | 0.667 | 0.738 | 0.487 |
| 22 | MEwhite | 0.955 | 0.462 | 0.878 |
| 6 | **MEsteelblue** | 0.996 | **0.002** | 0.878 |
| 7 | MEtan | 0.996 | 0.985 | 0.723 |
| 8 | MEdarkorange | 0.996 | 0.512 | 0.623 |
| 9 | MEbrown | 0.996 | 0.820 | 0.820 |
| 16 | MEpurple | 0.996 | 0.288 | 0.487 |
| 20 | MEdarkred | 0.996 | 0.462 | 0.623 |
| 21 | MEred | 0.996 | 0.985 | 0.623 |
| 24 | MElightcyan | 0.996 | 0.985 | 0.623 |
| 26 | **MEmidnightblue** | 0.996 | **0.004** | 0.053 |
| 27 | MEsalmon | 0.996 | 0.985 | 0.623 |

**Table S5**. List of genes in the significant age-related WGCNA module (MEgreen module #31, genes from main Figure 1B,C), logFC and FDR from DEG analysis by age using bulk RNA-seq (young vs aged, n=6; both sexes combined)

| **Gene** | **logFC in bulk RNA-seq DEG analysis by age** | **FDR** | **Name** |
| --- | --- | --- | --- |
| *Cdh4* | na | ns | cadherin 4 |
| *Ckmt1* | na | ns | creatine kinase, mitochondrial 1, ubiquitous |
| *Sez6* | na | ns | seizure related gene 6 |
| *Hoxb6* | na | ns | homeobox B6 |
| *Serpinf1* | na | ns | serine (or cysteine) peptidase inhibitor, clade F, member 1 |
| *S100a4* | 0.95 | 0.0373 | S100 calcium binding protein A4 |
| *S100a6* | na | ns | S100 calcium binding protein A6 (calcyclin) |
| *Icam2* | 0.6 | 0.0339 | intercellular adhesion molecule 2 |
| *B9d1* | na | ns | B9 protein domain 1 |
| *Timp1* | na | ns | tissue inhibitor of metalloproteinase 1 |
| *Lsr* | na | ns | lipolysis stimulated lipoprotein receptor |
| *Eef1e1* | na | ns | eukaryotic translation elongation factor 1 epsilon 1 |
| *Rac1* | na | ns | Rac family small GTPase 1 |
| *Blvra* | na | ns | biliverdin reductase A |
| *Unc119* | na | ns | unc-119 lipid binding chaperone |
| *Nudt14* | na | ns | nudix hydrolase 14 |
| *Nab1* | na | ns | Ngfi-A binding protein 1 |
| *Pnkp* | na | ns | polynucleotide kinase 3'- phosphatase |
| *Sri* | 0.29 | 0.041 | sorcin |
| *Sult2b1* | na | ns | sulfotransferase family, cytosolic, 2B, member 1 |
| *Dlgap1* | 1.71 | 0.0293 | DLG associated protein 1 |
| *Csnk1g2* | na | ns | casein kinase 1, gamma 2 |
| *St8sia6* | 0.69 | 0.0167 | ST8 alpha-N-acetyl-neuraminide alpha-2,8-sialyltransferase 6 |
| *Nosip* | na | ns | nitric oxide synthase interacting protein |
| *Dgcr6* | na | ns | DiGeorge syndrome critical region gene 6 |
| *Cyp17a1* | na | ns | cytochrome P450, family 17, subfamily a, polypeptide 1 |
| *Homer3* | na | ns | homer scaffolding protein 3 |
| *Has1* | na | ns | hyaluronan synthase 1 |
| *Nqo1* | na | ns | NAD(P)H dehydrogenase, quinone 1 |
| *Cd244a* | na | ns | CD244 molecule A |
| *Adgre1* | na | ns | adhesion G protein-coupled receptor E1 |
| *Nmrk2* | na | ns | nicotinamide riboside kinase 2 |
| *Arpc2* | na | ns | actin related protein 2/3 complex, subunit 2 |
| *Rps9* | na | ns | ribosomal protein S9 |
| *Crip1* | 0.68 | 0.01 | cysteine-rich protein 1 |
| *Neurl1a* | na | ns | neuralized E3 ubiquitin protein ligase 1A |
| *Ihh* | na | ns | Indian hedgehog |
| *Tph2* | na | ns | tryptophan hydroxylase 2 |
| *Id3* | na | ns | inhibitor of DNA binding 3 |
| *Crlf1* | na | ns | cytokine receptor-like factor 1 |
| *4930432K21Rik* | na | ns | Brme1, break repair meiotic recombinase recruitment factor 1 |
| *Sertad1* | na | ns | SERTA domain containing 1 |
| *Mgst1* | na | ns | microsomal glutathione S-transferase 1 |
| *Rps15a* | na | ns | ribosomal protein S15A |
| *Ccl8* | 3.26 | 0.0102 | C-C motif chemokine ligand 8 |
| *Syn2* | na | ns | synapsin II |
| *Rps25* | na | ns | ribosomal protein S25 |
| *Rassf1* | na | ns | Ras association (RalGDS/AF-6) domain family member 1 |
| *Spire2* | na | ns | spire type actin nucleation factor 2 |
| *Fads1* | na | ns | fatty acid desaturase 1 |
| *Gabra1* | na | ns | gamma-aminobutyric acid type A receptor subunit alpha 1 |
| *Chp1* | na | ns | calcineurin-like EF hand protein 1 |
| *Cluap1* | na | ns | clusterin associated protein 1 |
| *Pam16* | na | ns | presequence translocase-asssociated motor 16 |
| *Clec4f* | na | ns | C-type lectin domain family 4, member f |
| *Galns* | na | ns | galactosamine (N-acetyl)-6-sulfatase |
| *Npdc1* | na | ns | neural proliferation, differentiation and control 1 |
| *Cyp2j9* | na | ns | cytochrome P450, family 2, subfamily j, polypeptide 9 |
| *Mocs2* | na | ns | molybdenum cofactor synthesis 2 |
| *Anxa9* | na | ns | annexin A9 |
| *Lbp* | na | ns | lipopolysaccharide binding protein |
| *Hsd11b1* | na | ns | hydroxysteroid 11-beta dehydrogenase 1 |
| *Atp1b4* | na | ns | ATPase Na+/K+ transporting, beta 4 polypeptide |
| *Ift27* | na | ns | intraflagellar transport 27 |
| *Serinc3* | na | ns | serine incorporator 3 |
| *Cadps2* | na | ns | Ca2+-dependent activator protein for secretion 2 |
| *Sult4a1* | na | ns | sulfotransferase family 4A, member 1 |
| *Ccl6* | 1.02 | 0.0405 | C-C motif chemokine ligand 6 |
| *Ccl4* | na | ns | C-C motif chemokine ligand 4 |
| *Noxo1* | na | ns | NADPH oxidase organizer 1 |
| *Calm3* | na | ns | calmodulin 3 |
| *Ubb* | na | ns | ubiquitin B |
| *Akt3* | na | ns | thymoma viral proto-oncogene 3 |
| *Nts* | na | ns | neurotensin |
| *Dusp6* | na | ns | dual specificity phosphatase 6 |
| *Sgk1* | na | ns | serum/glucocorticoid regulated kinase 1 |
| *Cry1* | na | ns | cryptochrome circadian regulator 1 |
| *Pah* | 3.12 | 0.0038 | phenylalanine hydroxylase |
| *Mybpc1* | na | ns | myosin binding protein C, slow-type |
| *Srgn* | 0.88 | 0.0493 | serglycin |
| *2310011J03Rik* | na | ns | RIKEN cDNA 2310011J03 gene |
| *Sumo3* | na | ns | small ubiquitin-like modifier 3 |
| *Tpgs1* | na | ns | tubulin polyglutamylase complex subunit 1 |
| *Fstl3* | na | ns | follistatin-like 3 |
| *Tcn2* | na | ns | transcobalamin 2 |
| *Drg1* | na | ns | developmentally regulated GTP binding protein 1 |
| *Rtn4* | na | ns | reticulon 4 |
| *Fkbp1b* | na | ns | FK506 binding protein 1b |
| *Kif3c* | na | ns | kinesin family member 3C |
| *Sh3yl1* | na | ns | Sh3 domain YSC-like 1 |
| *Snx11* | na | ns | sorting nexin 11 |
| *Gfap* | na | ns | glial fibrillary acidic protein |
| *Nova1* | na | ns | NOVA alternative splicing regulator 1 |
| *Bdkrb2* | na | ns | bradykinin receptor, beta 2 |
| *Serpina3n* | 1.51 | 0.003 | serine (or cysteine) peptidase inhibitor, clade A, member 3N |
| *Gm20498* | na | ns | S2bpcox16, synaptojanin 2 binding protein Cox16 readthrough |
| *Ifi27l2b* | na | ns | interferon, alpha-inducible protein 27 like 2B |
| *Ppp4r4* | na | ns | protein phosphatase 4, regulatory subunit 4 |
| *Akr1c12* | na | ns | aldo-keto reductase family 1, member C12 |
| *Erg28* | na | ns | ergosterol biosynthesis 28 |
| *Aoah* | na | ns | acyloxyacyl hydrolase |
| *Serpinb9b* | na | ns | serine (or cysteine) peptidase inhibitor, clade B, member 9b |
| *Pxdc1* | na | ns | PX domain containing 1 |
| *Gadd45g* | na | ns | growth arrest and DNA-damage-inducible 45 gamma |
| *Nkd2* | na | ns | naked cuticle 2 |
| *Arsk* | na | ns | arylsulfatase K |
| *Btf3* | na | ns | basic transcription factor 3 |
| *Acox2* | na | ns | acyl-Coenzyme A oxidase 2, branched chain |
| *Arf4* | 0.28 | 0.0447 | ARF GTPase 4 |
| *Arhgef3* | na | ns | Rho guanine nucleotide exchange factor 3 |
| *Gdf10* | na | ns | growth differentiation factor 10 |
| *Cacna2d3* | na | ns | calcium channel, voltage-dependent, alpha2/delta subunit 3 |
| *C6* | na | ns | complement component 6 |
| *Dcstamp* | na | ns | dendrocyte expressed seven transmembrane protein |
| *Eif3e* | na | ns | eukaryotic translation initiation factor 3, subunit E |
| *Gm29394* | na | ns | predicted gene 29394 |
| *Litaf* | na | ns | LPS-induced TN factor |
| *Apod* | 1.49 | 0.0031 | apolipoprotein D |
| *Slurp1* | na | ns | secreted Ly6/Plaur domain containing 1 |
| *Arc* | na | ns | activity regulated cytoskeletal-associated protein |
| *Mpv17l* | na | ns | Mpv17 transgene, kidney disease mutant-like |
| *Ntan1* | na | ns | N-terminal Asn amidase |
| *Fetub* | na | ns | fetuin beta |
| *Cbr3* | na | ns | carbonyl reductase 3 |
| *Cela1* | 2.01 | 0.0364 | chymotrypsin-like elastase family, member 1 |
| *Soat2* | na | ns | sterol O-acyltransferase 2 |
| *Prr13* | na | ns | proline rich 13 |
| *Sncg* | 0.96 | 0.0295 | synuclein, gamma |
| *Gabrr2* | na | ns | gamma-aminobutyric acid type A receptor subunit rho 2 |
| *Spats1* | na | ns | spermatogenesis associated, serine-rich 1 |
| *Trem2* | 1.56 | 0.0076 | triggering receptor expressed on myeloid cells 2 |
| *C3* | na | ns | complement component 3 |
| *Thumpd2* | na | ns | THUMP domain containing 2 |
| *Btnl2* | na | ns | butyrophilin-like 2 |
| *Myot* | 1.11 | 0.0365 | myotilin |
| *Lox* | na | ns | lysyl oxidase |
| *Csf1r* | 0.37 | 0.0482 | colony stimulating factor 1 receptor |
| *Cyb5a* | 0.58 | 0.0416 | cytochrome b5 type A (microsomal) |
| *Anxa1* | 0.62 | 0.0196 | annexin A1 |
| *Tmem138* | na | ns | transmembrane protein 138 |
| *Ms4a2* | na | ns | membrane-spanning 4-domains, subfamily A, member 2 |
| *Ostf1* | na | ns | osteoclast stimulating factor 1 |
| *Trpm6* | 1.09 | 0.0471 | transient receptor potential cation channel, subfamily M, member 6 |
| *Ms4a8a* | na | ns | membrane-spanning 4-domains, subfamily A, member 8A |
| *Ccdc86* | na | ns | coiled-coil domain containing 86 |
| *Fas* | na | ns | Fas cell surface death receptor |
| *Rpp30* | na | ns | ribonuclease P/MRP 30 subunit |
| *Il33* | 0.63 | 0.0442 | interleukin 33 |
| *Shoc2* | na | ns | Shoc2, leucine rich repeat scaffold protein |
| *Fam45a* | na | ns | Dennd10, DENN domain containing 10 |
| *Smndc1* | na | ns | survival motor neuron domain containing 1 |
| *Maoa* | 0.56 | 0.0345 | monoamine oxidase A |
| *Rab40b* | na | ns | Rab40B, member RAS oncogene family |
| *Ankrd2* | na | ns | ankyrin repeat domain 2 |
| *Abcc2* | na | ns | ATP-binding cassette, sub-family C member 2 |
| *Pfkfb1* | na | ns | 6-phosphofructo-2-kinase/fructose-2,6-biphosphatase 1 |
| *Gpr143* | na | ns | G protein-coupled receptor 143 |
| *Adgra1* | na | ns | adhesion G protein-coupled receptor A1 |
| *Sumf2* | na | ns | sulfatase modifying factor 2 |
| *Hprt* | na | ns | Hprt1, hypoxanthine phosphoribosyltransferase 1 |
| *Pfkfb4* | na | ns | 6-phosphofructo-2-kinase/fructose-2,6-biphosphatase 4 |
| *Il6* | na | ns | interleukin 6 |
| *Rpl14* | na | ns | ribosomal protein L14 |
| *Unc5a* | na | ns | unc-5 netrin receptor A |
| *Casp12* | 0.88 | 0.0076 | caspase 12 |
| *Sgk3* | na | ns | serum/glucocorticoid regulated kinase 3 |
| *Gsta3* | na | ns | glutathione S-transferase, alpha 3 |
| *Ube2w* | na | ns | ubiquitin-conjugating enzyme E2W (putative) |
| *Cps1* | na | ns | carbamoyl-phosphate synthetase 1 |
| *Sumo1* | na | ns | small ubiquitin-like modifier 1 |
| *2010300C02Rik* | na | ns | Cracdl, capping protein inhibiting regulator of actin like |
| *Efhd1* | na | ns | EF hand domain containing 1 |
| *Htr4* | na | ns | 5 hydroxytryptamine (serotonin) receptor 4 |
| *3110009E18Rik* | na | ns | RIKEN cDNA 3110009E18 gene |
| *Arl8a* | na | ns | ADP-ribosylation factor-like 8A |
| *Rab29* | na | ns | RAB29, member RAS oncogene family |
| *Ifi211* | na | ns | interferon activated gene 211 |
| *Tagln2* | 0.53 | 0.0343 | transgelin 2 |
| *Slamf9* | 1.09 | 0.022 | SLAM family member 9 |
| *Selp* | na | ns | selectin, platelet |
| *Usf1* | na | ns | upstream transcription factor 1 |
| *Myoc* | na | ns | myocilin |
| *Dnajc1* | na | ns | DnaJ heat shock protein family (Hsp40) member C1 |
| *Nek6* | na | ns | NIMA (never in mitosis gene a)-related expressed kinase 6 |
| *Kif5c* | na | ns | kinesin family member 5C |
| *Slc25a25* | na | ns | solute carrier family 25 (mitochondrial carrier, phosphate carrier), member 25 |
| *Lcn2* | na | ns | lipocalin 2 |
| *Dpp7* | na | ns | dipeptidylpeptidase 7 |
| *Ssna1* | na | ns | SS nuclear autoantigen 1 |
| *Frzb* | na | ns | frizzled-related protein |
| *Dnajc10* | na | ns | DnaJ heat shock protein family (Hsp40) member C10 |
| *Bmp2* | na | ns | bone morphogenetic protein 2 |
| *Prom2* | 2.77 | 0.0339 | prominin 2 |
| *Tgm3* | na | ns | transglutaminase 3, E polypeptide |
| *Fam210b* | na | ns | family with sequence similarity 210, member B |
| *Stmn2* | na | ns | stathmin-like 2 |
| *Tpd52* | na | ns | tumor protein D52 |
| *Edn3* | 3.23 | 0.0031 | endothelin 3 |
| *Lrrcc1* | 0.84 | 0.004 | leucine rich repeat and coiled-coil domain containing 1 |
| *Postn* | 1.07 | 0.0026 | periostin, osteoblast specific factor |
| *Ptx3* | na | ns | pentraxin related gene |
| *Tspan2* | na | ns | tetraspanin 2 |
| *Hsd3b1* | na | ns | hydroxy-delta-5-steroid dehydrogenase, 3 beta- and steroid delta-isomerase 1 |
| *Mcub* | na | ns | mitochondrial calcium uniporter dominant negative beta subunit |
| *Tlr2* | na | ns | toll-like receptor 2 |
| *Ctso* | na | ns | cathepsin O |
| *Pdgfc* | na | ns | platelet-derived growth factor, C polypeptide |
| *Hcn3* | na | ns | hyperpolarization-activated, cyclic nucleotide-gated K+ 3 |
| *Rnf115* | 0.48 | 0.0178 | ring finger protein 115 |
| *Spata1* | na | ns | spermatogenesis associated 1 |
| *Sdcbp* | na | ns | syndecan binding protein |
| *2310002L09Rik* | na | ns | RIKEN cDNA 2310002L09 gene |
| *Chmp5* | na | ns | charged multivesicular body protein 5 |
| *Bnc2* | 0.79 | 0.007 | basonuclin zinc finger protein 2 |
| *Sh3gl2* | na | ns | SH3-domain GRB2-like 2 |
| *Artn* | na | ns | artemin |
| *Pramef12* | na | ns | PRAME like 12 |
| *Fuca1* | 0.39 | 0.0365 | fucosidase, alpha-L- 1, tissue |
| *Akr1a1* | na | ns | aldo-keto reductase family 1, member A1 |
| *Mrto4* | na | ns | mRNA turnover 4, ribosome maturation factor |
| *Pla2g2f* | na | ns | phospholipase A2, group IIF |
| *Pla2g2e* | na | ns | phospholipase A2, group IIE |
| *Cda* | na | ns | cytidine deaminase |
| *Sema3a* | na | ns | sema domain, immunoglobulin domain (Ig), short basic domain, secreted, (semaphorin) 3A |
| *Utp11* | na | ns | UTP11 small subunit processome component |
| *Rpl22* | na | ns | ribosomal protein L22 |
| *Abcb1b* | na | ns | ATP-binding cassette, sub-family B member 1B |
| *Prkcz* | na | ns | protein kinase C, zeta |
| *Sdf4* | na | ns | stromal cell derived factor 4 |
| *Cd38* | 0.55 | 0.0416 | CD38 antigen |
| *Kcnip4* | na | ns | Kv channel interacting protein 4 |
| *Hgfac* | na | ns | hepatocyte growth factor activator |
| *Ppp2r2c* | 2.49 | 0.0196 | protein phosphatase 2, regulatory subunit B, gamma |
| *Ociad2* | 0.67 | 0.0379 | OCIA domain containing 2 |
| *Cgref1* | na | ns | cell growth regulator with EF hand domain 1 |
| *Gabra4* | na | ns | gamma-aminobutyric acid type A receptor subunit alpha 4 |
| *Amtn* | na | ns | amelotin |
| *Plac8* | na | ns | placenta-specific 8 |
| *Cds1* | 1.16 | 0.0026 | CDP-diacylglycerol synthase 1 |
| *Fgf5* | na | ns | fibroblast growth factor 5 |
| *Nos1* | na | ns | nitric oxide synthase 1, neuronal |
| *Ereg* | na | ns | epiregulin |
| *Arpc3* | na | ns | actin related protein 2/3 complex, subunit 3 |
| *Chek2* | na | ns | checkpoint kinase 2 |
| *Slc46a3* | na | ns | solute carrier family 46, member 3 |
| *Tfpi2* | na | ns | tissue factor pathway inhibitor 2 |
| *Dlx6* | na | ns | distal-less homeobox 6 |
| *Akr1b8* | na | ns | aldo-keto reductase family 1, member B8 |
| *Gpnmb* | 1.22 | 0.035 | glycoprotein (transmembrane) nmb |
| *4921507P07Rik* | na | ns | Spmip4, sperm microtubule inner protein 4 |
| *Clec5a* | na | ns | C-type lectin domain family 5, member a |
| *Hpgds* | na | ns | hematopoietic prostaglandin D synthase |
| *Anxa4* | 0.42 | 0.0432 | annexin A4 |
| *Wnt7a* | na | ns | wingless-type MMTV integration site family, member 7A |
| *Lrrc23* | na | ns | leucine rich repeat containing 23 |
| *Rasgef1a* | na | ns | RasGEF domain family, member 1A |
| *Clec4a2* | na | ns | C-type lectin domain family 4, member a2 |
| *Plbd1* | 0.4 | 0.0442 | phospholipase B domain containing 1 |
| *Mgp* | na | ns | matrix Gla protein |
| *Atp2b2* | na | ns | ATPase, Ca++ transporting, plasma membrane 2 |
| *Tnfrsf1a* | na | ns | tumor necrosis factor receptor superfamily, member 1a |
| *Cyp2b10* | 3.9 | 0.004 | cytochrome P450, family 2, subfamily b, polypeptide 10 |
| *Gas2* | na | ns | growth arrest specific 2 |
| *Kctd15* | na | ns | potassium channel tetramerisation domain containing 15 |
| *Mphosph10* | na | ns | M-phase phosphoprotein 10 (U3 small nucleolar ribonucleoprotein) |
| *Lrrc28* | na | ns | leucine rich repeat containing 28 |
| *Nox4* | na | ns | NADPH oxidase 4 |
| *2200002D01Rik* | na | ns | RIKEN cDNA 2200002D01 gene |
| *Ryr1* | 2.22 | 0.0164 | ryanodine receptor 1, skeletal muscle |
| *Cdipt* | na | ns | CDP-diacylglycerol--inositol 3-phosphatidyltransferase |
| *Klk10* | na | ns | kallikrein related-peptidase 10 |
| *Itgam* | na | ns | integrin alpha M |
| *Chp2* | na | ns | calcineurin-like EF hand protein 2 |
| *Pdilt* | na | ns | protein disulfide isomerase-like, testis expressed |
| *Acsm5* | 1.7 | 0.0026 | acyl-CoA synthetase medium-chain family member 5 |
| *Akip1* | na | ns | A kinase interacting protein 1 |
| *Tnni2* | na | ns | troponin I, skeletal, fast 2 |
| *Igsf1* | na | ns | immunoglobulin superfamily, member 1 |
| *Arhgef6* | na | ns | Rac/Cdc42 guanine nucleotide exchange factor 6 |
| *F9* | na | ns | coagulation factor IX |
| *Syp* | na | ns | synaptophysin |
| *Rab39b* | na | ns | RAB39B, member RAS oncogene family |
| *Map3k15* | na | ns | mitogen-activated protein kinase kinase kinase 15 |
| *Cetn2* | na | ns | centrin 2 |
| *Pir* | na | ns | pirin |
| *Vegfd* | na | ns | vascular endothelial growth factor D |
| *Ggn* | na | ns | gametogenetin |
| *Cd209a* | na | ns | CD209a antigen |
| *Cd209d* | na | ns | CD209d antigen |
| *Tnfsf13b* | na | ns | tumor necrosis factor (ligand) superfamily, member 13b |
| *Adam5* | na | ns | a disintegrin and metallopeptidase domain 5 |
| *Asah1* | na | ns | N-acylsphingosine amidohydrolase 1 |
| *Cntnap4* | na | ns | contactin associated protein-like 4 |
| *Cx3cl1* | 0.64 | 0.0405 | C-X3-C motif chemokine ligand 1 |
| *Ces2g* | 0.63 | 0.0395 | carboxylesterase 2G |
| *Vstm5* | na | ns | V-set and transmembrane domain containing 5 |
| *Capn9* | na | ns | calpain 9 |
| *Adamts8* | na | ns | ADAM metallopeptidase with thrombospondin type 1 motif 8 |
| *Nxpe2* | na | ns | neurexophilin and PC-esterase domain family, member 2 |
| *Fez1* | na | ns | fasciculation and elongation protein zeta 1 |
| *Olfm2* | na | ns | olfactomedin 2 |
| *Lysmd2* | na | ns | LysM, putative peptidoglycan-binding, domain containing 2 |
| *Fam219b* | na | ns | family with sequence similarity 219, member B |
| *Ctsh* | 0.45 | 0.0258 | cathepsin H |
| *Parp16* | na | ns | poly (ADP-ribose) polymerase family, member 16 |
| *Nradd* | na | ns | neurotrophin receptor associated death domain |
| *Cpne4* | na | ns | copine IV |
| *Fhl3* | na | ns | four and a half LIM domains 3 |
| *Folr2* | na | ns | folate receptor beta |
| *Pram1* | na | ns | PML-RAR alpha-regulated adaptor molecule 1 |
| *Ilvbl* | na | ns | ilvB (bacterial acetolactate synthase)-like |
| *Trpa1* | na | ns | transient receptor potential cation channel, subfamily A, member 1 |
| *Srxn1* | na | ns | sulfiredoxin 1 homolog (S. cerevisiae) |
| *Cyb5r4* | na | ns | cytochrome b5 reductase 4 |
| *Adgre4* | na | ns | adhesion G protein-coupled receptor E4 |
| *Klra9* | 1.58 | 0.0167 | killer cell lectin-like receptor subfamily A, member 9 |
| *Crlf2* | 0.83 | 0.039 | cytokine receptor-like factor 2 |
| *Adgrb3* | na | ns | adhesion G protein-coupled receptor B3 |
| *Gabrb3* | na | ns | GABRB3, gamma-aminobutyric acid type A receptor subunit beta 3 |
| *Tlr13* | na | ns | toll-like receptor 13 |
| *B3galt2* | na | ns | UDP-Gal:betaGlcNAc beta 1,3-galactosyltransferase, polypeptide 2 |
| *Kcnk10* | na | ns | potassium channel, subfamily K, member 10 |
| *Kcnk1* | 4.04 | 0.0031 | potassium channel, subfamily K, member 1 |
| *Ccdc17* | na | ns | coiled-coil domain containing 17 |
| *Lyl1* | na | ns | lymphoblastomic leukemia 1 |
| *Tspan8* | na | ns | tetraspanin 8 |
| *Cpne2* | na | ns | copine II |
| *Pkd1l2* | na | ns | polycystic kidney disease 1 like 2 |
| *Slc24a1* | na | ns | solute carrier family 24 (sodium/potassium/calcium exchanger), member 1 |
| *Slc41a2* | 0.86 | 0.0295 | solute carrier family 41, member 2 |
| *Zyg11a* | na | ns | zyg-11 family member A, cell cycle regulator |
| *Ppp1r1c* | na | ns | protein phosphatase 1, regulatory inhibitor subunit 1C |
| *Grn* | na | ns | granulin |
| *Ccdc122* | na | ns | coiled-coil domain containing 122 |
| *Cpne7* | na | ns | copine VII |
| *Tmem106a* | na | ns | transmembrane protein 106A |
| *Hrh2* | na | ns | histamine receptor H2 |
| *Igsf6* | na | ns | immunoglobulin superfamily, member 6 |
| *Becn1* | na | ns | beclin 1, autophagy related |
| *Hpse* | na | ns | heparanase |
| *Tgfbi* | na | ns | transforming growth factor, beta induced |
| *Fbxl21* | na | ns | F-box and leucine-rich repeat protein 21 |
| *Ntng2* | na | ns | netrin G2 |
| *Gnptg* | na | ns | N-acetylglucosamine-1-phosphotransferase, gamma subunit |
| *Gdpd4* | na | ns | glycerophosphodiester phosphodiesterase domain containing 4 |
| *Ggta1* | na | ns | glycoprotein galactosyltransferase alpha 1, 3 |
| *Cep19* | na | ns | centrosomal protein 19 |
| *Cdhr3* | na | ns | cadherin-related family member 3 |
| *Slc2a6* | na | ns | solute carrier family 2 (facilitated glucose transporter), member 6 |
| *Lmo1* | na | ns | LIM domain only 1 |
| *Alx1* | na | ns | ALX homeobox 1 |
| *Dennd3* | na | ns | DENN domain containing 3 |
| *Psenen* | na | ns | presenilin enhancer gamma secretase subunit |
| *Zscan21* | na | ns | zinc finger and SCAN domain containing 21 |
| *Inhbb* | na | ns | inhibin beta-B |
| *Lrg1* | na | ns | leucine-rich alpha-2-glycoprotein 1 |
| *Tmprss13* | na | ns | transmembrane protease, serine 13 |
| *Krt80* | na | ns | keratin 80 |
| *Lsm1* | na | ns | LSM1 homolog, mRNA degradation associated |
| *Rims2* | na | ns | regulating synaptic membrane exocytosis 2 |
| *Tulp1* | na | ns | TUB like protein 1 |
| *Ephx3* | na | ns | epoxide hydrolase 3 |
| *Kctd8* | na | ns | potassium channel tetramerisation domain containing 8 |
| *Gpr160* | na | ns | G protein-coupled receptor 160 |
| *Armc3* | na | ns | armadillo repeat containing 3 |
| *Mat1a* | na | ns | methionine adenosyltransferase 1A |
| *1110032A03Rik* | na | ns | Cfap68, cilia and flagella associated protein 68 |
| *Dnah10* | na | ns | dynein, axonemal, heavy chain 10 |
| *Snx10* | na | ns | sorting nexin 10 |
| *Ankrd35* | na | ns | ankyrin repeat domain 35 |
| *Gdf15* | na | ns | growth differentiation factor 15 |
| *Rpf2* | na | ns | ribosome production factor 2 homolog |
| *C1s1* | 0.46 | 0.0327 | complement component 1, s subcomponent 1 |
| *Atf5* | na | ns | activating transcription factor 5 |
| *BC028528* | 0.8 | 0.0431 | cDNA sequence BC028528 |
| *Ciart* | na | ns | circadian associated repressor of transcription |
| *Vill* | na | ns | villin-like |
| *Ephx1* | 0.45 | 0.0316 | epoxide hydrolase 1, microsomal |
| *1700028J19Rik* | na | ns | RIKEN cDNA 1700028J19 gene |
| *Pi4kb* | na | ns | phosphatidylinositol 4-kinase beta |
| *Nipal2* | na | ns | NIPA-like domain containing 2 |
| *Rps21* | na | ns | ribosomal protein S21 |
| *Ak5* | na | ns | adenylate kinase 5 |
| *F13a1* | 0.49 | 0.0102 | coagulation factor XIII, A1 subunit |
| *Dap* | na | ns | death-associated protein |
| *Rpl22l1* | na | ns | ribosomal protein L22 like 1 |
| *Megf9* | na | ns | multiple EGF-like-domains 9 |
| *Igfbp2* | na | ns | insulin-like growth factor binding protein 2 |
| *Ccdc81* | na | ns | coiled-coil domain containing 81 |
| *Baat* | na | ns | bile acid-Coenzyme A: amino acid N-acyltransferase |
| *Batf2* | na | ns | basic leucine zipper transcription factor, ATF-like 2 |
| *Srrm3* | na | ns | serine/arginine repetitive matrix 3 |
| *Rhou* | na | ns | ras homolog family member U |
| *Fkbp6* | na | ns | FK506 binding protein 6 |
| *Mettl11b* | na | ns | Ntmt2, N-terminal Xaa-Pro-Lys N-methyltransferase 2 |
| *Pnrc1* | na | ns | proline-rich nuclear receptor coactivator 1 |
| *Fmo2* | na | ns | flavin containing monooxygenase 2 |
| *Gas8* | na | ns | growth arrest specific 8 |
| *Gpr34* | na | ns | G protein-coupled receptor 34 |
| *Alx4* | na | ns | aristaless-like homeobox 4 |
| *Blvrb* | na | ns | biliverdin reductase B |
| *Igsf23* | na | ns | immunoglobulin superfamily, member 23 |
| *Manea* | na | ns | mannosidase, endo-alpha |
| *Efhd2* | na | ns | EF hand domain containing 2 |
| *Chil3* | na | ns | chitinase-like 3 |
| *Zmynd15* | na | ns | zinc finger, MYND-type containing 15 |
| *Igsf21* | na | ns | immunoglobulin superfamily, member 21 |
| *Map7d2* | na | ns | MAP7 domain containing 2 |
| *Nbl1* | na | ns | NBL1, DAN family BMP antagonist |
| *H2az2* | na | ns | H2A.Z histone variant 2 |
| *Adgrf1* | na | ns | adhesion G protein-coupled receptor F1 |
| *Mdfic* | na | ns | MyoD family inhibitor domain containing |
| *Upk2* | na | ns | uroplakin 2 |
| *Selenow* | na | ns | selenoprotein W |
| *Prelp* | na | ns | proline arginine-rich end leucine-rich repeat |
| *Ggact* | na | ns | gamma-glutamylamine cyclotransferase |
| *Ddx24* | na | ns | DEAD box helicase 24 |
| *Trem3* | na | ns | triggering receptor expressed on myeloid cells 3 |
| *Ydjc* | na | ns | YdjC homolog (bacterial) |
| *Rpl37* | na | ns | ribosomal protein L37 |
| *Ccdc115* | na | ns | coiled-coil domain containing 115 |
| *Bend6* | 0.85 | 0.0442 | BEN domain containing 6 |
| *S100a13* | 0.41 | 0.0435 | S100 calcium binding protein A13 |
| *Ctf1* | na | ns | cardiotrophin 1 |
| *Gjb5* | na | ns | gap junction protein, beta 5 |
| *Atf4* | na | ns | activating transcription factor 4 |
| *AA986860* | na | ns | expressed sequence AA986860 |
| *Maff* | na | ns | v-maf musculoaponeurotic fibrosarcoma oncogene family, protein F (avian) |
| *Commd10* | na | ns | COMM domain containing 10 |
| *Map3k9* | na | ns | mitogen-activated protein kinase kinase kinase 9 |
| *Ttc9* | na | ns | tetratricopeptide repeat domain 9 |
| *Dpm3* | na | ns | dolichyl-phosphate mannosyltransferase polypeptide 3 |
| *Sgtb* | na | ns | small glutamine-rich tetratricopeptide repeat (TPR)-containing, beta |
| *Maneal* | na | ns | mannosidase, endo-alpha-like |
| *Trpv3* | na | ns | transient receptor potential cation channel, subfamily V, member 3 |
| *Pnpla1* | na | ns | patatin-like phospholipase domain containing 1 |
| *B3galnt1* | na | ns | UDP-GalNAc:betaGlcNAc beta 1,3-galactosaminyltransferase, polypeptide 1 |
| *Leng9* | na | ns | leukocyte receptor cluster (LRC) member 9 |
| *Pcdhb12* | na | ns | protocadherin beta 12 |
| *Rab9b* | na | ns | RAB9B, member RAS oncogene family |
| *Mmp3* | 1.42 | 0.0215 | matrix metallopeptidase 3 |
| *Tox3* | na | ns | TOX high mobility group box family member 3 |
| *B430306N03Rik* | na | ns | Trem6l, triggering receptor expressed on myeloid cells-like 6 |
| *Efcab9* | na | ns | EF-hand calcium binding domain 9 |
| *Tafa2* | na | ns | TAFA chemokine like family member 2 |
| *Bmerb1* | na | ns | bMERB domain containing 1 |
| *Trp53i13* | na | ns | transformation related protein 53 inducible protein 13 |
| *Lacc1* | na | ns | laccase domain containing 1 |
| *Sptssa* | na | ns | serine palmitoyltransferase, small subunit A |
| *Ascc1* | na | ns | activating signal cointegrator 1 complex subunit 1 |
| *Fzd1* | na | ns | frizzled class receptor 1 |
| *Ankrd45* | na | ns | ankyrin repeat domain 45 |
| *Cdc42ep2* | na | ns | CDC42 effector protein 2 |
| *Adrb2* | na | ns | adrenergic receptor, beta 2 |
| *Mtmr11* | na | ns | myotubularin related protein 11 |
| *Nxph1* | na | ns | neurexophilin 1 |
| *Pcdhb20* | na | ns | protocadherin beta 20 |
| *Iqub* | na | ns | IQ motif and ubiquitin domain containing |
| *Plaur* | na | ns | plasminogen activator, urokinase receptor |
| *Hepacam* | na | ns | hepatocyte cell adhesion molecule |
| *BC107364* | na | ns | cDNA sequence BC107364 |
| *Rbp1* | na | ns | retinol binding protein 1, cellular |
| *Scn4b* | 0.9 | 0.0178 | sodium channel, type IV, beta |
| *Lrrc8e* | na | ns | leucine rich repeat containing 8 family, member E |
| *Bdh1* | na | ns | 3-hydroxybutyrate dehydrogenase, type 1 |
| *Tifa* | na | ns | TRAF-interacting protein with forkhead-associated domain |
| *Kctd11* | na | ns | potassium channel tetramerisation domain containing 11 |
| *Fam187b* | na | ns | family with sequence similarity 187, member B |
| *Mettl21e* | na | ns | methyltransferase like 21E |
| *Wdr27* | na | ns | WD repeat domain 27 |
| *Prr15l* | na | ns | proline rich 15-like |
| *Pbp2* | na | ns | phosphatidylethanolamine binding protein 2 |
| *A2ml1* | na | ns | alpha-2-macroglobulin like 1 |
| *Cldn2* | na | ns | claudin 2 |
| *Pcdhb13* | na | ns | protocadherin beta 13 |
| *Kcne4* | na | ns | potassium voltage-gated channel, Isk-related subfamily, gene 4 |
| *Mettl21c* | na | ns | methyltransferase 21C, AARS1 lysine |
| *Dlgap2* | na | ns | DLG associated protein 2 |
| *Olfr1396* | na | ns | Or2v2, olfactory receptor family 2 subfamily V member 2 |
| *Nxpe5* | na | ns | neurexophilin and PC-esterase domain family, member 5 |
| *A430005L14Rik* | na | ns | RIKEN cDNA A430005L14 gene |
| *A4galt* | na | ns | alpha 1,4-galactosyltransferase |
| *Cyb5r2* | na | ns | cytochrome b5 reductase 2 |
| *Entpd1* | 0.36 | 0.0405 | ectonucleoside triphosphate diphosphohydrolase 1 |
| *Ric3* | 1.2 | 0.0026 | RIC3 acetylcholine receptor chaperone |
| *Pcdhb18* | na | ns | protocadherin beta 18 |
| *Pou4f1* | na | ns | POU domain, class 4, transcription factor 1 |
| *Omd* | na | ns | osteomodulin |
| *Cyp4f16* | na | ns | cytochrome P450, family 4, subfamily f, polypeptide 16 |
| *Msx1* | na | ns | msh homeobox 1 |
| *Zbtb8b* | na | ns | zinc finger and BTB domain containing 8b |
| *Myof* | na | ns | myoferlin |
| *Tprn* | na | ns | taperin |
| *Slc35e4* | na | ns | solute carrier family 35, member E4 |
| *Mrgpre* | na | ns | MAS-related GPR, member E |
| *C1galt1c1* | na | ns | C1GALT1-specific chaperone 1 |
| *Clec4a1* | na | ns | C-type lectin domain family 4, member a1 |
| *Pcdh10* | na | ns | protocadherin 10 |
| *Ccr2* | 1.24 | 0.0318 | C-C motif chemokine receptor 2 |
| *Rtl5* | na | ns | retrotransposon Gag like 5 |
| *Vgll2* | 2.44 | 0.0196 | vestigial like family member 2 |
| *Rpl36al* | na | ns | ribosomal protein L36A-like |
| *Shisa3* | 1.47 | 0.0327 | shisa family member 3 |
| *Tdrp* | 1.21 | 0.022 | testis development related protein |
| *Agmo* | na | ns | alkylglycerol monooxygenase |
| *Sprr1a* | na | ns | small proline-rich protein 1A |
| *Il31ra* | na | ns | interleukin 31 receptor A |
| *Lypd6* | 2.67 | 0.0219 | LY6/PLAUR domain containing 6 |
| *Sprr2f* | na | ns | small proline-rich protein 2F |
| *Tmem150c* | 0.83 | 0.014 | transmembrane protein 150C |
| *Ankrd37* | na | ns | ankyrin repeat domain 37 |
| *Dcaf12l2* | na | ns | DDB1 and CUL4 associated factor 12-like 2 |
| *Nat8f3* | na | ns | N-acetyltransferase 8 (GCN5-related) family member 3 |
| *Tlr6* | na | ns | toll-like receptor 6 |
| *Rtl8c* | na | ns | retrotransposon Gag like 8C |
| *Cd209f* | 1.26 | 0.0076 | CD209f antigen |
| *Rft1* | na | ns | RFT1 homolog |
| *C130026I21Rik* | 3.59 | 0.0235 | NA |
| *St6galnac3* | na | ns | ST6 (alpha-N-acetyl-neuraminyl-2,3-beta-galactosyl-1,3)-N-acetylgalactosaminide alpha-2,6-sialyltransferase 3 |
| *Cpne8* | 0.62 | 0.0215 | copine VIII |
| *Junb* | na | ns | jun B proto-oncogene |
| *Hrh1* | na | ns | histamine receptor H1 |
| *Sv2b* | na | ns | synaptic vesicle glycoprotein 2b |
| *Fank1* | na | ns | fibronectin type 3 and ankyrin repeat domains 1 |
| *Socs3* | na | ns | suppressor of cytokine signaling 3 |
| *Cdh22* | 2.33 | 0.0031 | cadherin 22 |
| *Smagp* | na | ns | small cell adhesion glycoprotein |
| *Ier2* | na | ns | immediate early response 2 |
| *Klk1b26* | 3.93 | 0.0081 | kallikrein 1-related petidase b26 |
| *Ceacam10* | na | ns | CEA cell adhesion molecule 10 |
| *Tprkb* | na | ns | Tp53rk binding protein |
| *Ceacam2* | na | ns | CEA cell adhesion molecule 2 |
| *Dnajc8* | na | ns | DnaJ heat shock protein family (Hsp40) member C8 |
| *Spcs3* | na | ns | signal peptidase complex subunit 3 homolog (S. cerevisiae) |
| *Gm9951* | na | ns | predicted gene 9951 |
| *Klhl5* | 0.36 | 0.0443 | kelch-like 5 |
| *Cntn1* | na | ns | contactin 1 |
| *Pdlim1* | na | ns | PDZ and LIM domain 1 (elfin) |
| *Klf2* | na | ns | Kruppel-like transcription factor 2 (lung) |
| *C1ra* | 0.41 | 0.0438 | complement component 1, r subcomponent A |
| *Ano5* | 2.1 | 0.0026 | anoctamin 5 |
| *Epha6* | na | ns | Eph receptor A6 |
| *Tescl* | na | ns | tescalcin-like |
| *Rnf181* | na | ns | ring finger protein 181 |
| *Cldn23* | na | ns | claudin 23 |
| *9330182L06Rik* | na | ns | Elapor2 (endosome-lysosome associated apoptosis and autophagy regulator family member 2) |
| *Clca3a1* | na | ns | chloride channel accessory 3A1 |
| *Snx32* | na | ns | sorting nexin 32 |
| *Lman1l* | na | ns | lectin, mannose-binding 1 like |
| *Ptprn2* | na | ns | protein tyrosine phosphatase receptor type N polypeptide 2 |
| *Trnp1* | na | ns | TMF1-regulated nuclear protein 1 |
| *Ier5* | na | ns | immediate early response 5 |
| *A730036I17Rik* | na | ns | RIKEN cDNA A730036I17 gene |
| *Hamp2* | na | ns | hepcidin antimicrobial peptide 2 |
| *Nxf3* | na | ns | nuclear RNA export factor 3 |
| *BC051142* | na | ns | testis expressed basic protein 1 |
| *St6galnac2* | na | ns | ST6 (alpha-N-acetyl-neuraminyl-2,3-beta-galactosyl-1,3)-N-acetylgalactosaminide alpha-2,6-sialyltransferase 2 |
| *Rpl38* | na | ns | ribosomal protein L38 |
| *Sphk2* | na | ns | sphingosine kinase 2 |
| *Uxs1* | na | ns | UDP-glucuronate decarboxylase 1 |
| *Bloc1s2* | na | ns | biogenesis of lysosomal organelles complex-1, subunit 2 |
| *Pus7* | na | ns | pseudouridylate synthase 7 |
| *Tmem178b* | na | ns | transmembrane protein 178B |
| *Gm6169* | na | ns | Plp2rt (proteolipid protein 2, retrotransposed) |
| *Abat* | 1.03 | 0.0343 | 4-aminobutyrate aminotransferase |
| *Gsta2* | 2.16 | 0.0178 | glutathione S-transferase, alpha 2 (Yc2) |
| *Nfam1* | na | ns | Nfat activating molecule with ITAM motif 1 |
| *Serpina9* | na | ns | serine (or cysteine) peptidase inhibitor, clade A (alpha-1 antiproteinase, antitrypsin), member 9 |
| *Tmed9* | na | ns | transmembrane p24 trafficking protein 9 |
| *Fcer1g* | na | ns | Fc receptor, IgE, high affinity I, gamma polypeptide |
| *Kcnj14* | na | ns | potassium inwardly-rectifying channel, subfamily J, member 14 |
| *Pirb* | 0.86 | 0.0471 | paired Ig-like receptor B |
| *Cfi* | na | ns | complement component factor i |
| *Kcnc1* | na | ns | potassium voltage gated channel, Shaw-related subfamily, member 1 |
| *Mfsd4a* | na | ns | major facilitator superfamily domain containing 4A |
| *Skap2* | 1.02 | 0.0052 | src family associated phosphoprotein 2 |
| *Vpreb1* | na | ns | V-set pre-B cell surrogate light chain |
| *Fcgr3* | 0.73 | 0.0167 | Fc receptor, IgG, low affinity III |
| *Alox5ap* | na | ns | arachidonate 5-lipoxygenase activating protein |
| *Fam78b* | na | ns | family with sequence similarity 78, member B |
| *Rpl26* | na | ns | ribosomal protein L26 |
| *Dmkn* | na | ns | dermokine |
| *Retnla* | na | ns | resistin like alpha |
| *Wdr61* | na | ns | Skic8 (SKI8 subunit of superkiller complex) |
| *Olfr12* | na | ns | Or9s13 (olfactory receptor family 9 subfamily S member 13) |
| *Tnnt3* | na | ns | troponin T3, skeletal, fast |
| *Slc22a12* | na | ns | solute carrier family 22 (organic anion/cation transporter), member 12 |
| *BC048679* | na | ns | cDNA sequence BC048679 |
| *Zscan20* | na | ns | zinc finger and SCAN domains 20 |
| *Fmr1nb* | na | ns | Fmr1 neighbor |
| *Rpl17* | na | ns | ribosomal protein L17 |
| *Cytl1* | na | ns | cytokine-like 1 |
| *Ap3b2* | na | ns | adaptor-related protein complex 3, beta 2 subunit |
| *Csprs* | 2.51 | 0.0026 | component of Sp100-rs |
| *Manbal* | na | ns | mannosidase, beta A, lysosomal-like |
| *Cd300lb* | na | ns | CD300 molecule like family member B |
| *Tmed5* | na | ns | transmembrane p24 trafficking protein 5 |
| *Gm10131* | na | ns | Erhrt-ps (ERH mRNA splicing and mitosis factor retrotransposed, pseudogene) |
| *Rps15* | na | ns | ribosomal protein S15 |
| *Arhgap22* | na | ns | Rho GTPase activating protein 22 |
| *Cdc42ep5* | na | ns | CDC42 effector protein 5 |
| *Gpx1* | na | ns | glutathione peroxidase 1 |
| *Cntn4* | na | ns | contactin 4 |
| *Aox3* | na | ns | aldehyde oxidase 3 |
| *Selenop* | na | ns | selenoprotein P |
| *Serpina3c* | na | ns | serine (or cysteine) peptidase inhibitor, clade A, member 3C |
| *Pilrb2* | na | ns | paired immunoglobin-like type 2 receptor beta 2 |
| *Serpinb7* | na | ns | serine (or cysteine) peptidase inhibitor, clade B, member 7 |
| *Nnat* | na | ns | neuronatin |
| *Ccdc87* | na | ns | coiled-coil domain containing 87 |
| *Zfp760* | na | ns | zinc finger protein 760 |
| *Gm10226* | na | ns | Gm10509 (predicted gene 10509) |
| *Ninl* | na | ns | ninein-like |
| *Psma5* | na | ns | proteasome subunit alpha 5 |
| *Rps18-ps6* | na | ns | ribosomal protein S18, pseudogene 6 |
| *Fgfr1op* | na | ns | Cep43 (centrosomal protein 43) |
| *Lyz1* | na | ns | lysozyme 1 |
| *Cd300ld3* | na | ns | CD300 molecule like family member D3 |
| *Nkain2* | na | ns | Na+/K+ transporting ATPase interacting 2 |
| *Wfdc17* | 0.85 | 0.0215 | WAP four-disulfide core domain 17 |
| *Fam149a* | na | ns | family with sequence similarity 149, member A |
| *Mmp27* | na | ns | matrix metallopeptidase 27 |
| *Tlnrd1* | na | ns | talin rod domain containing 1 |
| *Cldn3* | na | ns | claudin 3 |
| *Tmem132b* | na | ns | transmembrane protein 132B |
| *Nsmce3* | na | ns | NSE3 homolog, SMC5-SMC6 complex component |
| *Slc17a7* | na | ns | solute carrier family 17 (sodium-dependent inorganic phosphate cotransporter), member 7 |
| *Lactbl1* | na | ns | lactamase, beta-like 1 |
| *Ces1a* | na | ns | carboxylesterase 1A |
| *Jund* | na | ns | jun D proto-oncogene |
| *Serpina1d* | na | ns | serine (or cysteine) peptidase inhibitor, clade A, member 1D |
| *Serpina1b* | 2.86 | 0.0031 | serine (or cysteine) preptidase inhibitor, clade A, member 1B |
| *Naip5* | 1.19 | 0.0435 | NLR family, apoptosis inhibitory protein 5 |
| *Psmg4* | na | ns | proteasome (prosome, macropain) assembly chaperone 4 |
| *Pcnp* | na | ns | PEST proteolytic signal containing nuclear protein |
| *Gm10339* | na | ns | RIKEN cDNA 4930521O11 gene |
| *Serpina1e* | 2.67 | 0.0113 | serine (or cysteine) peptidase inhibitor, clade A, member 1E |
| *Trpc5os* | na | ns | transient receptor potential cation channel, subfamily C, member 5, opposite strand |
| *Sod3* | na | ns | superoxide dismutase 3, extracellular |
| *Cfap47* | na | ns | cilia and flagella associated protein 47 |
| *Vma21* | na | ns | VMA21 vacuolar H+-ATPase homolog (S. cerevisiae) |
| *Trim40* | na | ns | tripartite motif-containing 40 |
| *Lst1* | na | ns | leukocyte specific transcript 1 |
| *C4b* | 1.39 | 0.0006 | complement C4B (Chido blood group) |
| *Cyp4f15* | na | ns | cytochrome P450, family 4, subfamily f, polypeptide 15 |
| *Mtarc2* | na | ns | mitochondrial amidoxime reducing component 2 |
| *Ifi204* | 0.81 | 0.0076 | interferon activated gene 204 |
| *Ifi207* | na | ns | interferon activated gene 207 |
| *Pcdhb22* | na | ns | protocadherin beta 22 |
| *Ecscr* | na | ns | endothelial cell surface expressed chemotaxis and apoptosis regulator |
| *Cdkn2b* | na | ns | cyclin dependent kinase inhibitor 2B |
| *Osgin1* | na | ns | oxidative stress induced growth inhibitor 1 |
| *Gsta1* | na | ns | glutathione S-transferase, alpha 1 (Ya) |
| *Amy1* | 1.57 | 0.0026 | amylase 1, salivary |
| *Tnfaip8l3* | na | ns | tumor necrosis factor, alpha-induced protein 8-like 3 |
| *Sprr2a3* | na | ns | small proline-rich protein 2A3 |
| *Bglap3* | na | ns | bone gamma-carboxyglutamate protein 3 |
| *Kif5a* | na | ns | kinesin family member 5A |
| *Syndig1* | na | ns | synapse differentiation inducing 1 |
| *Ism1* | na | ns | isthmin 1, angiogenesis inhibitor |
| *Zfp808* | na | ns | zinc finger protein 808 |
| *Gm10800* | na | ns | predicted gene, 75297 |
| *Gm10801* | na | ns | No associated gene |
| *Pde11a* | na | ns | phosphodiesterase 11A |
| *Sarnp* | na | ns | SAP domain containing ribonucleoprotein |
| *Zfp984* | na | ns | zinc finger protein 984 |
| *Gm13212* | 0.45 | 0.0416 | NA |
| *Ceacam15* | na | ns | CEA cell adhesion molecule 15 |
| *Gm14322* | na | ns | predicted gene 14322 |
| *Gm14440* | na | ns | Zfp1009 (zinc finger protein 1009) |
| *Col22a1* | na | ns | collagen, type XXII, alpha 1 |
| *Kcnip3* | na | ns | Kv channel interacting protein 3, calsenilin |
| *Capn3* | 1.38 | 0.0178 | calpain 3 |
| *Cd209g* | 1.2 | 0.0295 | CD209g antigen |
| *Ccr5* | na | ns | C-C motif chemokine receptor 5 |
| *Gm43302* | na | ns | predicted gene 43302 |
| *Gm8334* | na | ns | predicted gene 8334 |
| *Atg4a* | na | ns | autophagy related 4A, cysteine peptidase |
| *Ms4a6c* | 1.1 | 0.0497 | membrane-spanning 4-domains, subfamily A, member 6C |
| *Rpl36a* | na | ns | ribosomal protein L36A |
| *Gm7609* | 2.04 | 0.0026 | predicted pseudogene 7609 |
| *Rpl39* | na | ns | ribosomal protein L39 |
| *Prrt4* | na | ns | proline-rich transmembrane protein 4 |
| *Eef1akmt3* | na | ns | EEF1A lysine methyltransferase 3 |
| *Gm16741* | na | ns | predicted gene, 16741 |
| *5031425F14Rik* | na | ns | RIKEN cDNA 5031425F14 gene |
| *D630024D03Rik* | na | ns | RIKEN cDNA D630024D03 gene |
| *Gm15728* | na | ns | predicted gene 15728 |
| *Gm16933* | na | ns | predicted gene, 16933 |
| *Gm15609* | na | ns | predicted gene 15609 |
| *Gm6117* | na | ns | predicted gene 6117 |
| *5330413P13Rik* | na | ns | RIKEN cDNA 5330413P13 gene |
| *Gm15627* | na | ns | predicted gene 15627 |
| *1500009L16Rik* | na | ns | RIKEN cDNA 1500009L16 gene |
| *Gm16025* | na | ns | predicted gene 16025 |
| *Tnfsf13* | na | ns | tumor necrosis factor (ligand) superfamily, member 13 |
| *Cd300ld5* | na | ns | CD300 molecule like family member D5 |
| *Ugt1a7c* | na | ns | UDP glucuronosyltransferase 1 family, polypeptide A7C |
| *Apold1* | na | ns | apolipoprotein L domain containing 1 |
| *Gm6871* | na | ns | predicted gene 6871 |
| *Rps13* | na | ns | ribosomal protein S13 |
| *Gm17300* | na | ns | predicted gene, 17300 |
| *Gm8206* | na | ns | predicted gene 8206 |
| *Vmn2r3* | na | ns | vomeronasal 2, receptor 3 |
| *Gm17349* | na | ns |  |
| *Vmn2r95* | na | ns | vomeronasal 2, receptor 95 |
| *Yy2* | na | ns | Yy2 transcription factor |
| *Cyp2d10* | na | ns | cytochrome P450, family 2, subfamily d, polypeptide 10 |
| *Gm3604* | na | ns | predicted gene 3604 |
| *Gm10718* | na | ns |  |
| *Gm21738* | na | ns |  |
| *AC132444.5* | na | ns |  |
| *Gm10719* | 2.11 | 0.043 |  |
| *Gm21188* | 1.31 | 0.035 | NA |
| *Csta2* | na | ns | cystatin A family member 2 |
| *CAAA01147332.1* | na | ns |  |
| *Sirpb1a* | na | ns | signal-regulatory protein beta 1A |
| *Vkorc1* | na | ns | vitamin K epoxide reductase complex, subunit 1 |
| *Ccl21b* | na | ns | C-C motif chemokine ligand 21B (leucine) |
| *Gm21814* | na | ns | predicted gene, 21814 |
| *Gm11168* | na | ns |  |
| *Gm10721* | na | ns |  |
| *Gm16863* | na | ns | predicted gene, 16863 |
| *Gm26685* | na | ns | predicted gene, 26685 |
| *Gm26645* | na | ns | predicted gene, 26645 |
| *4833412C05Rik* | na | ns | RIKEN cDNA 4833412C05 gene |
| *Gm16701* | na | ns | predicted gene, 16701 |
| *Gm20757* | na | ns | predicted gene, 20757 |
| *Gm5493* | na | ns | predicted gene 5493 |
| *Pet117* | na | ns | PET117 homolog |
| *Commd1b* | na | ns | COMM domain containing 1B |
| *2900052N01Rik* | na | ns | RIKEN cDNA 2900052N01 gene |
| *Gm7160* | na | ns | predicted gene 7160 |
| *Gm29264* | na | ns | predicted gene 29264 |
| *Hoxd4* | na | ns | homeobox D4 |
| *4930568A12Rik* | na | ns | RIKEN cDNA 4930568A12 gene |
| *Gm29291* | na | ns | No associated gene |
| *2310016D03Rik* | na | ns | RIKEN cDNA 2310016D03 gene |
| *Gm38246* | na | ns | predicted gene, 38246 |
| *Gm37988* | na | ns | predicted gene, 37988 |
| *Gm43064* | na | ns | predicted gene 43064 |
| *Gbp10* | na | ns | guanylate-binding protein 10 |
| *Gm19710* | na | ns | predicted gene, 19710 |
| *9430085M18Rik* | na | ns | RIKEN cDNA 9430085M18 gene |
| *Gm43101* | na | ns | predicted gene 43101 |
| *Gm9495* | na | ns | predicted gene, 38944 |
| *Olfr1310* | na | ns | Or4f6 (olfactory receptor family 4 subfamily F member 6) |
| *Gm38944* | na | ns | predicted gene, 38944 |
| *Gm44698* | na | ns | No associated gene |
| *Gm42417* | na | ns | predicted gene, 42417 |
| *Gm42397* | na | ns | predicted gene, 42397 |
| *Gm45253* | na | ns | predicted gene 45253 |
| *Brd3os* | na | ns | bromodomain containing 3, opposite strand |
| *Gm45717* | na | ns | predicted gene 45717 |
| *Gm5086* | na | ns | predicted gene 5086 |
| *Gm48362* | na | ns | predicted gene, 48362 |
| *Gm7607* | na | ns | predicted gene 7607 |
| *Gm3776* | na | ns | Gsta13 (glutathione S-transferase alpha 13) |
| *Gm39464* | na | ns | predicted gene, 39464 |
| *Gm48610* | na | ns | No associated gene |
| *Gm36283* | na | ns | predicted gene, 36283 |
| *A730063M14Rik* | na | ns | RIKEN cDNA A730063M14 gene |
| *Gm48116* | na | ns | predicted gene, 48116 |
| *Gm40658* | na | ns | predicted gene, 40658 |
| *BB123696* | na | ns | expressed sequence BB123696 |
| *Gm47507* | na | ns | predicted gene, 47507 |
| *Gm36161* | 1.98 | 0.0026 | predicted gene, 36161 |
| *Gm41307* | na | ns | predicted gene, 41307 |
| *Bc1* | na | ns | brain cytoplasmic RNA 1 |
| *Gm41349* | na | ns | predicted gene, 41349 |
| *Gm49450* | na | ns | predicted gene, 49450 |
| *Gm30124* | na | ns | predicted gene, 30124 |
| *Gm49756* | na | ns | predicted gene, 49756 |
| *Gm49891* | na | ns | predicted gene, 49891 |
| *Gm31615* | na | ns | predicted gene, 31615 |
| *1810073O08Rik* | na | ns | RIKEN cDNA 1810073O08 gene |
| *Gm31706* | na | ns | predicted gene, 31706 |
| *Gm50216* | na | ns | predicted gene, 50216 |
| *Gm41804* | na | ns | predicted gene, 41804 |
| *AC167036.1* | 2.43 | 0.0167 |  |
| *AC147806.1* | na | ns | No associated gene |
| *AC147806.2* | 2.92 | 0.0006 | No associated gene |

**Table S6.** Aging differentially expressed genes in macrophages; snRNA-seq; 6- vs 24-month-old C57BL/6J mice, n=3 per age/sex; adj.p-value<0.05, |log2FC| >0.36

| ***Gene*** | **avg_log2FC** | **p_val_adj** | **Up/Down DEG** | **group** | **cell** | **Both sexes, males, females** |
| --- | --- | --- | --- | --- | --- | --- |
| *Cd36* | 0.57 | 5.7E-15 | up | age | macrophage | both sexes combined |
| *Rasgrp3* | 0.56 | 2.7E-16 | up | age | macrophage | both sexes combined |
| *Ccr5* | 0.55 | 2.7E-16 | up | age | macrophage | both sexes combined |
| *Gm20619* | 0.51 | 2.5E-02 | up | age | macrophage | both sexes combined |
| *Ddx60* | 0.49 | 4.3E-11 | up | age | macrophage | both sexes combined |
| *Pdgfc* | 0.48 | 7.1E-12 | up | age | macrophage | both sexes combined |
| *C130026I21Rik* | 0.48 | 3.9E-27 | up | age | macrophage | both sexes combined |
| *Wwp1* | 0.47 | 4.9E-22 | up | age | macrophage | both sexes combined |
| *Igf1* | 0.46 | 1.3E-11 | up | age | macrophage | both sexes combined |
| *Apobec1* | 0.46 | 8.8E-18 | up | age | macrophage | both sexes combined |
| *Ftl1* | 0.43 | 2.5E-12 | up | age | macrophage | both sexes combined |
| *Ube2e2* | 0.42 | 1.9E-08 | up | age | macrophage | both sexes combined |
| *2610203C22Rik* | 0.41 | 7.5E-19 | up | age | macrophage | both sexes combined |
| *Apbb1ip* | 0.40 | 1.4E-16 | up | age | macrophage | both sexes combined |
| *AC149090.1* | 0.40 | 3.5E-14 | up | age | macrophage | both sexes combined |
| *Myo1e* | 0.40 | 4.1E-04 | up | age | macrophage | both sexes combined |
| *Fam214a* | 0.39 | 1.5E-08 | up | age | macrophage | both sexes combined |
| *Cacna1a* | 0.39 | 3.4E-10 | up | age | macrophage | both sexes combined |
| *Clec12a* | 0.39 | 8.6E-14 | up | age | macrophage | both sexes combined |
| *Gm21188* | 0.39 | 4.6E-15 | up | age | macrophage | both sexes combined |
| *Lilr4b* | 0.37 | 6.6E-13 | up | age | macrophage | both sexes combined |
| *Nrp1* | 0.36 | 4.7E-12 | up | age | macrophage | both sexes combined |
| *Gpr155* | 0.36 | 1.8E-11 | up | age | macrophage | both sexes combined |
| *Fgd4* | 0.36 | 1.9E-10 | up | age | macrophage | both sexes combined |
| *Antxr2* | -0.36 | 3.3E-15 | down | age | macrophage | both sexes combined |
| *Rfx2* | -0.37 | 1.7E-17 | down | age | macrophage | both sexes combined |
| *Lars2* | -0.38 | 9.6E-22 | down | age | macrophage | both sexes combined |
| *Wdfy2* | -0.38 | 4.5E-24 | down | age | macrophage | both sexes combined |
| *Man2a1* | -0.38 | 1.6E-25 | down | age | macrophage | both sexes combined |
| *Ttn* | -0.40 | 1.7E-07 | down | age | macrophage | both sexes combined |
| *Cmss1* | -0.52 | 2.7E-33 | down | age | macrophage | both sexes combined |
| *Stxbp6* | -0.55 | 1.6E-12 | down | age | macrophage | both sexes combined |
| *Gm42418* | -0.57 | 2.2E-79 | down | age | macrophage | both sexes combined |
| *Ccnd3* | -0.58 | 7.2E-33 | down | age | macrophage | both sexes combined |
| *AY036118* | -0.68 | 4.9E-76 | down | age | macrophage | both sexes combined |
| *Ccr5* | 0.75 | 4.24E-14 | up | age | macrophage | male |
| *Ddx60* | 0.59 | 5.42E-05 | up | age | macrophage | male |
| *Cdk6* | 0.54 | 1.20E-08 | up | age | macrophage | male |
| *2610203C22Rik* | 0.52 | 3.87E-14 | up | age | macrophage | male |
| *Clec12a* | 0.51 | 1.02E-07 | up | age | macrophage | male |
| *Gm4951* | 0.46 | 1.75E-02 | up | age | macrophage | male |
| *Rasgrp3* | 0.46 | 4.46E-04 | up | age | macrophage | male |
| *Apobec1* | 0.45 | 3.38E-05 | up | age | macrophage | male |
| *C130026I21Rik* | 0.42 | 1.36E-07 | up | age | macrophage | male |
| *Igf1* | 0.42 | 1.67E-02 | up | age | macrophage | male |
| *Ly6e* | 0.41 | 1.64E-05 | up | age | macrophage | male |
| *Wwp1* | 0.41 | 9.72E-05 | up | age | macrophage | male |
| *Cmah* | 0.40 | 1.46E-03 | up | age | macrophage | male |
| *Fgd4* | 0.40 | 1.93E-04 | up | age | macrophage | male |
| *Cers6* | 0.38 | 3.09E-02 | up | age | macrophage | male |
| *Mir99ahg* | 0.38 | 7.29E-08 | up | age | macrophage | male |
| *Gm20559* | 0.37 | 7.91E-05 | up | age | macrophage | male |
| *Apbb1ip* | 0.36 | 8.41E-04 | up | age | macrophage | male |
| *Rapgef6* | 0.36 | 1.35E-03 | up | age | macrophage | male |
| *Hba-a2* | -0.36 | 7.02E-19 | down | age | macrophage | male |
| *Dlc1* | -0.37 | 1.29E-03 | down | age | macrophage | male |
| *Myl2* | -0.37 | 1.36E-07 | down | age | macrophage | male |
| *Myl3* | -0.37 | 4.32E-06 | down | age | macrophage | male |
| *Tnni3* | -0.38 | 5.56E-08 | down | age | macrophage | male |
| *Hbb-bt* | -0.38 | 8.44E-16 | down | age | macrophage | male |
| *Ppm1h* | -0.39 | 3.80E-09 | down | age | macrophage | male |
| *Fabp3* | -0.41 | 8.60E-09 | down | age | macrophage | male |
| *Atp2a2* | -0.44 | 1.90E-13 | down | age | macrophage | male |
| *Rfx2* | -0.45 | 1.15E-11 | down | age | macrophage | male |
| *Stxbp6* | -0.48 | 4.20E-02 | down | age | macrophage | male |
| *Mb* | -0.48 | 5.29E-16 | down | age | macrophage | male |
| *Pln* | -0.50 | 6.46E-14 | down | age | macrophage | male |
| *Actc1* | -0.51 | 1.03E-12 | down | age | macrophage | male |
| *Efcab11* | -0.51 | 2.63E-02 | down | age | macrophage | male |
| *Ccnd3* | -0.59 | 4.17E-14 | down | age | macrophage | male |
| *Tpm1* | -0.59 | 7.23E-24 | down | age | macrophage | male |
| *Hbb-bs* | -0.59 | 3.52E-42 | down | age | macrophage | male |
| *Ryr2* | -0.60 | 7.75E-10 | down | age | macrophage | male |
| *Tnnt2* | -0.61 | 5.87E-26 | down | age | macrophage | male |
| *Lars2* | -0.66 | 3.23E-43 | down | age | macrophage | male |
| *Ttn* | -0.71 | 4.07E-36 | down | age | macrophage | male |
| *Myh6* | -0.82 | 7.11E-48 | down | age | macrophage | male |
| *Cmss1* | -0.86 | 2.43E-64 | down | age | macrophage | male |
| *Gm42418* | -0.92 | 4.68E-114 | down | age | macrophage | male |
| *AY036118* | -1.11 | 4.50E-92 | down | age | macrophage | male |
| *Hbb-bs* | 1.46 | 7.70E-87 | up | age | macrophage | female |
| *Hba-a1* | 1.28 | 1.28E-73 | up | age | macrophage | female |
| *Hba-a2* | 0.98 | 8.79E-51 | up | age | macrophage | female |
| *Cd36* | 0.80 | 1.11E-18 | up | age | macrophage | female |
| *Tpm1* | 0.75 | 1.23E-33 | up | age | macrophage | female |
| *Rasgrp3* | 0.74 | 6.14E-10 | up | age | macrophage | female |
| *Wwp1* | 0.73 | 4.39E-30 | up | age | macrophage | female |
| *Dcn* | 0.72 | 4.76E-32 | up | age | macrophage | female |
| *Fam214a* | 0.71 | 2.24E-10 | up | age | macrophage | female |
| *Fabp3* | 0.67 | 1.74E-22 | up | age | macrophage | female |
| *Myl3* | 0.67 | 4.76E-17 | up | age | macrophage | female |
| *Actc1* | 0.66 | 4.75E-15 | up | age | macrophage | female |
| *Gpr155* | 0.66 | 9.77E-14 | up | age | macrophage | female |
| *Mb* | 0.63 | 3.81E-15 | up | age | macrophage | female |
| *Cox4i1* | 0.63 | 1.11E-18 | up | age | macrophage | female |
| *Hbb-bt* | 0.62 | 1.63E-25 | up | age | macrophage | female |
| *Myl2* | 0.61 | 1.75E-22 | up | age | macrophage | female |
| *Cox6a2* | 0.61 | 2.45E-13 | up | age | macrophage | female |
| *AC149090.1* | 0.58 | 3.33E-13 | up | age | macrophage | female |
| *Rora* | 0.58 | 2.62E-04 | up | age | macrophage | female |
| *Gsn* | 0.57 | 3.85E-20 | up | age | macrophage | female |
| *Rps8* | 0.57 | 9.55E-15 | up | age | macrophage | female |
| *Pdgfc* | 0.57 | 2.18E-04 | up | age | macrophage | female |
| *St8sia4* | 0.56 | 6.84E-11 | up | age | macrophage | female |
| *Tnnt2* | 0.56 | 3.74E-18 | up | age | macrophage | female |
| *Fabp4* | 0.55 | 1.70E-20 | up | age | macrophage | female |
| *Slc25a4* | 0.55 | 1.92E-10 | up | age | macrophage | female |
| *Ftl1* | 0.54 | 2.85E-09 | up | age | macrophage | female |
| *Lpl* | 0.52 | 1.27E-07 | up | age | macrophage | female |
| *Cst3* | 0.51 | 4.35E-12 | up | age | macrophage | female |
| *Apbb1ip* | 0.49 | 3.88E-10 | up | age | macrophage | female |
| *Mgp* | 0.48 | 7.12E-15 | up | age | macrophage | female |
| *Ddx60* | 0.48 | 8.03E-05 | up | age | macrophage | female |
| *Nrp1* | 0.47 | 2.21E-14 | up | age | macrophage | female |
| *Cd38* | 0.47 | 7.33E-03 | up | age | macrophage | female |
| *Igf1* | 0.47 | 2.78E-05 | up | age | macrophage | female |
| *Tnnc1* | 0.46 | 8.65E-12 | up | age | macrophage | female |
| *Atp5g1* | 0.45 | 3.15E-08 | up | age | macrophage | female |
| *Arhgap17* | 0.45 | 1.28E-09 | up | age | macrophage | female |
| *Fgd4* | 0.45 | 2.28E-07 | up | age | macrophage | female |
| *Tnni3* | 0.44 | 1.55E-08 | up | age | macrophage | female |
| *Cryab* | 0.43 | 2.82E-07 | up | age | macrophage | female |
| *Ly86* | 0.43 | 2.24E-06 | up | age | macrophage | female |
| *Pln* | 0.43 | 6.85E-07 | up | age | macrophage | female |
| *Selenop* | 0.42 | 3.16E-08 | up | age | macrophage | female |
| *Fth1* | 0.42 | 7.91E-09 | up | age | macrophage | female |
| *2610203C22Rik* | 0.42 | 2.37E-04 | up | age | macrophage | female |
| *Rapgef6* | 0.42 | 2.86E-05 | up | age | macrophage | female |
| *Rpl6* | 0.41 | 2.26E-04 | up | age | macrophage | female |
| *Ubb* | 0.40 | 5.82E-08 | up | age | macrophage | female |
| *Ndufb9* | 0.40 | 4.06E-07 | up | age | macrophage | female |
| *Rpl13* | 0.40 | 6.17E-07 | up | age | macrophage | female |
| *Lair1* | 0.39 | 1.98E-03 | up | age | macrophage | female |
| *Apobec1* | 0.39 | 3.31E-05 | up | age | macrophage | female |
| *Lyz2* | 0.39 | 7.82E-06 | up | age | macrophage | female |
| *Cox7c* | 0.39 | 4.49E-04 | up | age | macrophage | female |
| *Rpl21* | 0.38 | 3.19E-04 | up | age | macrophage | female |
| *Gm21188* | 0.38 | 1.33E-02 | up | age | macrophage | female |
| *Ankrd1* | 0.38 | 1.20E-11 | up | age | macrophage | female |
| *C130026I21Rik* | 0.38 | 3.34E-04 | up | age | macrophage | female |
| *Tpt1* | 0.37 | 2.70E-02 | up | age | macrophage | female |
| *Atp5h* | 0.37 | 6.00E-07 | up | age | macrophage | female |
| *Ctsc* | 0.37 | 8.54E-10 | up | age | macrophage | female |
| *Ifi203* | 0.37 | 1.95E-03 | up | age | macrophage | female |
| *Ifitm3* | 0.36 | 1.66E-03 | up | age | macrophage | female |
| *Rpl9* | 0.36 | 5.83E-04 | up | age | macrophage | female |
| *Dpyd* | 0.36 | 1.40E-02 | up | age | macrophage | female |
| *Ndufa4* | 0.36 | 2.12E-04 | up | age | macrophage | female |
| *Lilr4b* | 0.36 | 2.17E-04 | up | age | macrophage | female |
| *Zswim6* | -0.36 | 4.09E-13 | down | age | macrophage | female |
| *Gng2* | -0.36 | 2.33E-05 | down | age | macrophage | female |
| *Hivep2* | -0.36 | 2.04E-02 | down | age | macrophage | female |
| *Kif23* | -0.37 | 3.72E-07 | down | age | macrophage | female |
| *Baz1a* | -0.38 | 8.44E-08 | down | age | macrophage | female |
| *Rfx2* | -0.39 | 1.05E-07 | down | age | macrophage | female |
| *Rassf2* | -0.39 | 7.45E-12 | down | age | macrophage | female |
| *Runx1* | -0.39 | 5.87E-07 | down | age | macrophage | female |
| *Jdp2* | -0.39 | 1.01E-06 | down | age | macrophage | female |
| *Creb5* | -0.40 | 3.37E-04 | down | age | macrophage | female |
| *Galnt2* | -0.41 | 8.21E-08 | down | age | macrophage | female |
| *Lrrfip1* | -0.41 | 2.02E-09 | down | age | macrophage | female |
| *Man1a* | -0.44 | 1.51E-11 | down | age | macrophage | female |
| *Gas7* | -0.45 | 6.52E-03 | down | age | macrophage | female |
| *Dennd4a* | -0.45 | 3.36E-09 | down | age | macrophage | female |
| *App* | -0.46 | 7.19E-16 | down | age | macrophage | female |
| *Mrtfa* | -0.48 | 3.11E-09 | down | age | macrophage | female |
| *Cyth1* | -0.48 | 3.47E-13 | down | age | macrophage | female |
| *Entpd1* | -0.49 | 1.67E-08 | down | age | macrophage | female |
| *Pag1* | -0.49 | 3.81E-11 | down | age | macrophage | female |
| *Zdhhc14* | -0.50 | 9.06E-10 | down | age | macrophage | female |
| *Cmip* | -0.50 | 4.41E-25 | down | age | macrophage | female |
| *Wdfy2* | -0.52 | 2.23E-20 | down | age | macrophage | female |
| *Ccnd3* | -0.53 | 1.36E-12 | down | age | macrophage | female |
| *Antxr2* | -0.55 | 1.99E-13 | down | age | macrophage | female |
| *Cnnm2* | -0.59 | 2.92E-02 | down | age | macrophage | female |
| *Lrrc8d* | -0.59 | 5.22E-10 | down | age | macrophage | female |
| *Stxbp6* | -0.61 | 4.45E-05 | down | age | macrophage | female |
| *Cd44* | -0.63 | 1.10E-08 | down | age | macrophage | female |
| *Man2a1* | -0.70 | 1.62E-22 | down | age | macrophage | female |
| *Fkbp5* | -0.71 | 1.68E-20 | down | age | macrophage | female |
| *Pde3b* | -0.92 | 1.04E-29 | down | age | macrophage | female |

**Table S7.** Top ten enriched Gene Ontology terms for Biological Process (GO BP) based on age up- and down-regulated DEGs in macrophages; snRNA-seq, both sex combined, adj.p-value<0.05, |log2FC| >0.36

| **Term** | **Overlap** | **Adj.P.value** | **Genes** | **GeneRatio** | **comparison** | **cell_type** | **direction** |
| --- | --- | --- | --- | --- | --- | --- | --- |
| Cellular Response to Amyloid-Beta (GO:1904646) | 3/36 | 2.79E-03 | CACNA1A;IGF1;CD36 | 0.14 | Aging | Macrophage | up, both |
| Response to Amyloid-Beta (GO:1904645) | 3/45 | 2.79E-03 | CACNA1A;IGF1;CD36 | 0.14 | Aging | Macrophage | up, both |
| Positive Regulation of ERK1 and ERK2 Cascade (GO:0070374) | 4/153 | 2.79E-03 | NRP1;PDGFC;IGF1;CD36 | 0.18 | Aging | Macrophage | up, both |
| Regulation of ERK1 and ERK2 Cascade (GO:0070372) | 4/223 | 8.07E-03 | NRP1;PDGFC;IGF1;CD36 | 0.18 | Aging | Macrophage | up, both |
| Vascular Endothelial Growth Factor Signaling Pathway (GO:0038084) | 2/14 | 8.07E-03 | NRP1;MYO1E | 0.09 | Aging | Macrophage | up, both |
| Positive Regulation of Smooth Muscle Cell Migration (GO:0014911) | 2/17 | 8.60E-03 | NRP1;IGF1 | 0.09 | Aging | Macrophage | up, both |
| Platelet-Derived Growth Factor Receptor Signaling Pathway (GO:0048008) | 2/17 | 8.60E-03 | NRP1;PDGFC | 0.09 | Aging | Macrophage | up, both |
| Regulation of Smooth Muscle Cell Migration (GO:0014910) | 2/19 | 9.45E-03 | NRP1;IGF1 | 0.09 | Aging | Macrophage | up, both |
| Cell Surface Receptor Protein Tyrosine Kinase Signaling Pathway (GO:0007169) | 4/296 | 1.20E-02 | NRP1;MYO1E;PDGFC;IGF1 | 0.18 | Aging | Macrophage | up, both |
| Positive Regulation of Fibroblast Proliferation (GO:0048146) | 2/28 | 1.66E-02 | PDGFC;IGF1 | 0.09 | Aging | Macrophage | up, both |
| SNARE Complex Assembly (GO:0035493) | 1/9 | 8.33E-02 | STXBP6 | 0.09 | Aging | Macrophage | down, both |
| Negative Regulation of Exocytosis (GO:0045920) | 1/11 | 8.33E-02 | STXBP6 | 0.09 | Aging | Macrophage | down, both |
| N-glycan Processing (GO:0006491) | 1/21 | 8.33E-02 | MAN2A1 | 0.09 | Aging | Macrophage | down, both |
| tRNA Aminoacylation (GO:0043039) | 1/22 | 8.33E-02 | LARS2 | 0.09 | Aging | Macrophage | down, both |
| Positive Regulation of G1/S Transition of Mitotic Cell Cycle (GO:1900087) | 1/33 | 8.33E-02 | CCND3 | 0.09 | Aging | Macrophage | down, both |
| tRNA Aminoacylation for Protein Translation (GO:0006418) | 1/36 | 8.33E-02 | LARS2 | 0.09 | Aging | Macrophage | down, both |
| Negative Regulation of Secretion by Cell (GO:1903531) | 1/41 | 8.33E-02 | STXBP6 | 0.09 | Aging | Macrophage | down, both |
| Positive Regulation of Cell Cycle G1/S Phase Transition (GO:1902808) | 1/44 | 8.33E-02 | CCND3 | 0.09 | Aging | Macrophage | down, both |
| Regulation of Exocytosis (GO:0017157) | 1/48 | 8.33E-02 | STXBP6 | 0.09 | Aging | Macrophage | down, both |
| Positive Regulation of Fat Cell Differentiation (GO:0045600) | 1/50 | 8.33E-02 | WDFY2 | 0.09 | Aging | Macrophage | down, both |

**Table S8.** Aging differentially expressed genes in *Gsn^+^* fibroblasts; snRNA-seq; 6- vs 24-month-old C57BL/6J mice, n=3 per age/sex; adj.p-value<0.05, |log2FC| >0.36

| **Gene** | **avg_log2FC** | **p_val_adj** | **Up/Down DEG** | **group** | **cell** | **Both sexes, males, females** |
| --- | --- | --- | --- | --- | --- | --- |
| *Errfi1* | 1.01 | 5.8E-46 | up | age | Gsn+ fib | both sexes combined |
| *Igfbp7* | 0.70 | 2.9E-134 | up | age | Gsn+ fib | both sexes combined |
| *Il1r1* | 0.64 | 1.3E-73 | up | age | Gsn+ fib | both sexes combined |
| *Col8a1* | 0.62 | 7.9E-115 | up | age | Gsn+ fib | both sexes combined |
| *Naaladl2* | 0.60 | 1.0E-110 | up | age | Gsn+ fib | both sexes combined |
| *Adamtsl3* | 0.60 | 1.5E-89 | up | age | Gsn+ fib | both sexes combined |
| *Atp8b1* | 0.60 | 6.8E-81 | up | age | Gsn+ fib | both sexes combined |
| *Abca8a* | 0.58 | 2.7E-101 | up | age | Gsn+ fib | both sexes combined |
| *Rhoj* | 0.58 | 2.9E-88 | up | age | Gsn+ fib | both sexes combined |
| *Lsamp* | 0.57 | 7.3E-89 | up | age | Gsn+ fib | both sexes combined |
| *Cyth3* | 0.56 | 4.9E-87 | up | age | Gsn+ fib | both sexes combined |
| *Gfpt2* | 0.56 | 3.2E-89 | up | age | Gsn+ fib | both sexes combined |
| *Abca6* | 0.54 | 1.4E-82 | up | age | Gsn+ fib | both sexes combined |
| *Fstl1* | 0.54 | 1.5E-99 | up | age | Gsn+ fib | both sexes combined |
| *Gab2* | 0.54 | 6.5E-98 | up | age | Gsn+ fib | both sexes combined |
| *Mgp* | 0.53 | 1.6E-70 | up | age | Gsn+ fib | both sexes combined |
| *Nox4* | 0.53 | 5.7E-52 | up | age | Gsn+ fib | both sexes combined |
| *AC149090.1* | 0.53 | 3.6E-99 | up | age | Gsn+ fib | both sexes combined |
| *Pi16* | 0.53 | 3.2E-56 | up | age | Gsn+ fib | both sexes combined |
| *Grik2* | 0.52 | 1.7E-65 | up | age | Gsn+ fib | both sexes combined |
| *Lama2* | 0.52 | 2.6E-106 | up | age | Gsn+ fib | both sexes combined |
| *Pcolce2* | 0.52 | 1.9E-78 | up | age | Gsn+ fib | both sexes combined |
| *Ly6a* | 0.51 | 4.8E-74 | up | age | Gsn+ fib | both sexes combined |
| *Anks1b* | 0.51 | 4.7E-73 | up | age | Gsn+ fib | both sexes combined |
| *Ebf2* | 0.51 | 1.4E-109 | up | age | Gsn+ fib | both sexes combined |
| *Tenm3* | 0.51 | 5.2E-106 | up | age | Gsn+ fib | both sexes combined |
| *Ngf* | 0.50 | 3.0E-74 | up | age | Gsn+ fib | both sexes combined |
| *Alpk1* | 0.50 | 2.8E-55 | up | age | Gsn+ fib | both sexes combined |
| *Col14a1* | 0.50 | 8.0E-56 | up | age | Gsn+ fib | both sexes combined |
| *Malat1* | 0.50 | 1.4E-158 | up | age | Gsn+ fib | both sexes combined |
| *Fbn1* | 0.50 | 2.0E-88 | up | age | Gsn+ fib | both sexes combined |
| *Gclc* | 0.49 | 7.1E-34 | up | age | Gsn+ fib | both sexes combined |
| *Neat1* | 0.49 | 1.2E-103 | up | age | Gsn+ fib | both sexes combined |
| *Thsd7a* | 0.49 | 1.4E-82 | up | age | Gsn+ fib | both sexes combined |
| *C1qtnf7* | 0.49 | 1.4E-67 | up | age | Gsn+ fib | both sexes combined |
| *Smoc2* | 0.49 | 2.2E-91 | up | age | Gsn+ fib | both sexes combined |
| *Agmo* | 0.49 | 5.9E-85 | up | age | Gsn+ fib | both sexes combined |
| *Egfr* | 0.48 | 1.7E-74 | up | age | Gsn+ fib | both sexes combined |
| *Kcnt2* | 0.48 | 1.8E-94 | up | age | Gsn+ fib | both sexes combined |
| *Pcdh9* | 0.48 | 4.1E-101 | up | age | Gsn+ fib | both sexes combined |
| *Col5a2* | 0.48 | 7.2E-101 | up | age | Gsn+ fib | both sexes combined |
| *Mir100hg* | 0.48 | 4.4E-91 | up | age | Gsn+ fib | both sexes combined |
| *Fndc1* | 0.48 | 5.4E-59 | up | age | Gsn+ fib | both sexes combined |
| *Medag* | 0.48 | 3.4E-76 | up | age | Gsn+ fib | both sexes combined |
| *Bnc2* | 0.47 | 8.6E-81 | up | age | Gsn+ fib | both sexes combined |
| *Col4a4* | 0.47 | 1.2E-82 | up | age | Gsn+ fib | both sexes combined |
| *Mfap5* | 0.47 | 6.9E-66 | up | age | Gsn+ fib | both sexes combined |
| *Itga5* | 0.47 | 2.2E-41 | up | age | Gsn+ fib | both sexes combined |
| *Zbtb7c* | 0.47 | 7.4E-67 | up | age | Gsn+ fib | both sexes combined |
| *Stk32b* | 0.47 | 1.2E-70 | up | age | Gsn+ fib | both sexes combined |
| *Fbln2* | 0.47 | 2.1E-72 | up | age | Gsn+ fib | both sexes combined |
| *Ccn1* | 0.47 | 5.2E-46 | up | age | Gsn+ fib | both sexes combined |
| *Adamts5* | 0.47 | 2.9E-69 | up | age | Gsn+ fib | both sexes combined |
| *Ghr* | 0.47 | 3.3E-84 | up | age | Gsn+ fib | both sexes combined |
| *Sparc* | 0.47 | 5.5E-85 | up | age | Gsn+ fib | both sexes combined |
| *Col4a1* | 0.46 | 7.7E-66 | up | age | Gsn+ fib | both sexes combined |
| *Magi2* | 0.46 | 3.3E-66 | up | age | Gsn+ fib | both sexes combined |
| *Robo1* | 0.46 | 1.5E-69 | up | age | Gsn+ fib | both sexes combined |
| *Postn* | 0.46 | 5.3E-17 | up | age | Gsn+ fib | both sexes combined |
| *Cd34* | 0.46 | 2.9E-81 | up | age | Gsn+ fib | both sexes combined |
| *Islr* | 0.46 | 1.4E-39 | up | age | Gsn+ fib | both sexes combined |
| *Cpq* | 0.46 | 5.2E-102 | up | age | Gsn+ fib | both sexes combined |
| *Ltbp4* | 0.46 | 4.6E-76 | up | age | Gsn+ fib | both sexes combined |
| *Colec12* | 0.45 | 1.1E-74 | up | age | Gsn+ fib | both sexes combined |
| *Plscr4* | 0.45 | 8.0E-74 | up | age | Gsn+ fib | both sexes combined |
| *Lama4* | 0.45 | 2.0E-90 | up | age | Gsn+ fib | both sexes combined |
| *B3galt1* | 0.45 | 1.1E-89 | up | age | Gsn+ fib | both sexes combined |
| *Svep1* | 0.45 | 1.6E-60 | up | age | Gsn+ fib | both sexes combined |
| *Negr1* | 0.45 | 1.5E-81 | up | age | Gsn+ fib | both sexes combined |
| *Col5a3* | 0.44 | 1.2E-82 | up | age | Gsn+ fib | both sexes combined |
| *Itgbl1* | 0.44 | 1.4E-70 | up | age | Gsn+ fib | both sexes combined |
| *Mast4* | 0.44 | 1.8E-78 | up | age | Gsn+ fib | both sexes combined |
| *Sh3d19* | 0.44 | 1.5E-84 | up | age | Gsn+ fib | both sexes combined |
| *Zfpm2* | 0.43 | 6.0E-78 | up | age | Gsn+ fib | both sexes combined |
| *Sdc2* | 0.43 | 4.0E-84 | up | age | Gsn+ fib | both sexes combined |
| *Col3a1* | 0.43 | 1.8E-68 | up | age | Gsn+ fib | both sexes combined |
| *Morrbid* | 0.43 | 5.8E-68 | up | age | Gsn+ fib | both sexes combined |
| *Abca8b* | 0.43 | 1.8E-66 | up | age | Gsn+ fib | both sexes combined |
| *Col4a2* | 0.43 | 3.4E-61 | up | age | Gsn+ fib | both sexes combined |
| *Lsp1* | 0.43 | 7.2E-87 | up | age | Gsn+ fib | both sexes combined |
| *Sgcd* | 0.42 | 1.6E-65 | up | age | Gsn+ fib | both sexes combined |
| *Bicc1* | 0.42 | 2.1E-81 | up | age | Gsn+ fib | both sexes combined |
| *Plxdc2* | 0.42 | 2.7E-89 | up | age | Gsn+ fib | both sexes combined |
| *Nalcn* | 0.42 | 1.8E-71 | up | age | Gsn+ fib | both sexes combined |
| *Prkg1* | 0.42 | 4.3E-74 | up | age | Gsn+ fib | both sexes combined |
| *Rbms3* | 0.42 | 1.3E-89 | up | age | Gsn+ fib | both sexes combined |
| *Dchs2* | 0.42 | 4.0E-46 | up | age | Gsn+ fib | both sexes combined |
| *Serping1* | 0.42 | 2.6E-78 | up | age | Gsn+ fib | both sexes combined |
| *Pcolce* | 0.42 | 1.6E-71 | up | age | Gsn+ fib | both sexes combined |
| *Clec3b* | 0.42 | 3.1E-56 | up | age | Gsn+ fib | both sexes combined |
| *Plpp3* | 0.42 | 1.1E-52 | up | age | Gsn+ fib | both sexes combined |
| *Gulp1* | 0.41 | 3.1E-89 | up | age | Gsn+ fib | both sexes combined |
| *B4galt5* | 0.41 | 7.2E-50 | up | age | Gsn+ fib | both sexes combined |
| *Vcl* | 0.41 | 8.9E-60 | up | age | Gsn+ fib | both sexes combined |
| *Smad3* | 0.41 | 1.1E-62 | up | age | Gsn+ fib | both sexes combined |
| *Dcn* | 0.41 | 9.1E-41 | up | age | Gsn+ fib | both sexes combined |
| *Foxp2* | 0.41 | 7.0E-92 | up | age | Gsn+ fib | both sexes combined |
| *Sp100* | 0.41 | 4.3E-69 | up | age | Gsn+ fib | both sexes combined |
| *Cd55* | 0.40 | 4.0E-52 | up | age | Gsn+ fib | both sexes combined |
| *Plcl1* | 0.40 | 2.1E-59 | up | age | Gsn+ fib | both sexes combined |
| *Emp1* | 0.40 | 5.9E-74 | up | age | Gsn+ fib | both sexes combined |
| *Bcl2l1* | 0.40 | 1.1E-46 | up | age | Gsn+ fib | both sexes combined |
| *Ebf1* | 0.40 | 1.5E-79 | up | age | Gsn+ fib | both sexes combined |
| *Gpc6* | 0.40 | 1.6E-78 | up | age | Gsn+ fib | both sexes combined |
| *Pakap.1* | 0.40 | 1.7E-48 | up | age | Gsn+ fib | both sexes combined |
| *Mir99ahg* | 0.40 | 2.1E-75 | up | age | Gsn+ fib | both sexes combined |
| *Prickle2* | 0.40 | 3.6E-59 | up | age | Gsn+ fib | both sexes combined |
| *Dok6* | 0.40 | 2.1E-36 | up | age | Gsn+ fib | both sexes combined |
| *Lhfpl2* | 0.39 | 1.5E-52 | up | age | Gsn+ fib | both sexes combined |
| *B4galt1* | 0.39 | 3.2E-67 | up | age | Gsn+ fib | both sexes combined |
| *Cp* | 0.39 | 2.6E-65 | up | age | Gsn+ fib | both sexes combined |
| *Pcsk6* | 0.39 | 9.8E-82 | up | age | Gsn+ fib | both sexes combined |
| *Cped1* | 0.39 | 4.2E-78 | up | age | Gsn+ fib | both sexes combined |
| *H2-D1* | 0.39 | 4.6E-58 | up | age | Gsn+ fib | both sexes combined |
| *Ahnak2* | 0.39 | 2.7E-85 | up | age | Gsn+ fib | both sexes combined |
| *Trps1* | 0.39 | 1.3E-76 | up | age | Gsn+ fib | both sexes combined |
| *Ptgis* | 0.38 | 8.5E-57 | up | age | Gsn+ fib | both sexes combined |
| *Adgrl3* | 0.38 | 7.7E-39 | up | age | Gsn+ fib | both sexes combined |
| *Xdh* | 0.38 | 7.1E-63 | up | age | Gsn+ fib | both sexes combined |
| *Unc5c* | 0.38 | 2.7E-37 | up | age | Gsn+ fib | both sexes combined |
| *Cdk14* | 0.38 | 6.0E-76 | up | age | Gsn+ fib | both sexes combined |
| *Gxylt2* | 0.38 | 1.2E-55 | up | age | Gsn+ fib | both sexes combined |
| *Sulf1* | 0.38 | 2.0E-72 | up | age | Gsn+ fib | both sexes combined |
| *Fgfr1* | 0.38 | 1.0E-79 | up | age | Gsn+ fib | both sexes combined |
| *Pvt1* | 0.38 | 3.0E-68 | up | age | Gsn+ fib | both sexes combined |
| *Igfbp6* | 0.38 | 3.6E-33 | up | age | Gsn+ fib | both sexes combined |
| *Pard3b* | 0.38 | 1.5E-78 | up | age | Gsn+ fib | both sexes combined |
| *Rhoq* | 0.38 | 2.7E-55 | up | age | Gsn+ fib | both sexes combined |
| *Abca9* | 0.38 | 1.1E-58 | up | age | Gsn+ fib | both sexes combined |
| *Runx1t1* | 0.37 | 7.5E-70 | up | age | Gsn+ fib | both sexes combined |
| *Npas2* | 0.37 | 2.6E-56 | up | age | Gsn+ fib | both sexes combined |
| *Akt3* | 0.37 | 7.2E-84 | up | age | Gsn+ fib | both sexes combined |
| *Apbb1ip* | 0.37 | 1.6E-57 | up | age | Gsn+ fib | both sexes combined |
| *Col6a2* | 0.37 | 1.3E-60 | up | age | Gsn+ fib | both sexes combined |
| *Ftl1* | 0.37 | 3.4E-63 | up | age | Gsn+ fib | both sexes combined |
| *Vcan* | 0.37 | 1.1E-72 | up | age | Gsn+ fib | both sexes combined |
| *Sfrp1* | 0.37 | 1.2E-49 | up | age | Gsn+ fib | both sexes combined |
| *Son* | 0.37 | 1.0E-90 | up | age | Gsn+ fib | both sexes combined |
| *Ctsl* | 0.37 | 6.1E-61 | up | age | Gsn+ fib | both sexes combined |
| *Abi3bp* | 0.37 | 3.5E-71 | up | age | Gsn+ fib | both sexes combined |
| *Kcnq1ot1* | 0.37 | 4.2E-74 | up | age | Gsn+ fib | both sexes combined |
| *Prelp* | 0.37 | 3.3E-43 | up | age | Gsn+ fib | both sexes combined |
| *Celf2* | 0.36 | 3.7E-76 | up | age | Gsn+ fib | both sexes combined |
| *Ecm1* | 0.36 | 2.5E-45 | up | age | Gsn+ fib | both sexes combined |
| *Rsrp1* | 0.36 | 1.0E-94 | up | age | Gsn+ fib | both sexes combined |
| *Airn* | 0.36 | 2.7E-77 | up | age | Gsn+ fib | both sexes combined |
| *Ifitm3* | 0.36 | 1.7E-60 | up | age | Gsn+ fib | both sexes combined |
| *Lum* | 0.36 | 9.9E-60 | up | age | Gsn+ fib | both sexes combined |
| *Man1a* | 0.36 | 3.0E-79 | up | age | Gsn+ fib | both sexes combined |
| *Dclk1* | 0.36 | 1.1E-50 | up | age | Gsn+ fib | both sexes combined |
| *Ext1* | 0.36 | 1.0E-73 | up | age | Gsn+ fib | both sexes combined |
| *Loxl1* | 0.36 | 9.2E-61 | up | age | Gsn+ fib | both sexes combined |
| *Nkain2* | 0.36 | 4.4E-09 | up | age | Gsn+ fib | both sexes combined |
| *Dock7* | 0.36 | 2.0E-77 | up | age | Gsn+ fib | both sexes combined |
| *Exoc4* | 0.36 | 1.4E-66 | up | age | Gsn+ fib | both sexes combined |
| *Frmd4a* | 0.36 | 2.1E-55 | up | age | Gsn+ fib | both sexes combined |
| *Cntln* | 0.36 | 4.4E-61 | up | age | Gsn+ fib | both sexes combined |
| *Kif13b* | 0.36 | 6.8E-85 | up | age | Gsn+ fib | both sexes combined |
| *Cdk8* | -0.42 | 3.1E-04 | down | age | Gsn+ fib | both sexes combined |
| *Myl4* | -0.46 | 1.9E-05 | down | age | Gsn+ fib | both sexes combined |
| *Mb* | -0.46 | 1.5E-14 | down | age | Gsn+ fib | both sexes combined |
| *Fabp3* | -0.48 | 8.9E-03 | down | age | Gsn+ fib | both sexes combined |
| *Lpl* | -0.48 | 1.9E-14 | down | age | Gsn+ fib | both sexes combined |
| *Myl2* | -0.49 | 8.8E-16 | down | age | Gsn+ fib | both sexes combined |
| *Tmtc2* | -0.50 | 2.5E-02 | down | age | Gsn+ fib | both sexes combined |
| *Actc1* | -0.51 | 1.7E-19 | down | age | Gsn+ fib | both sexes combined |
| *Hrc* | -0.52 | 4.7E-04 | down | age | Gsn+ fib | both sexes combined |
| *Ndufa4* | -0.53 | 1.6E-02 | down | age | Gsn+ fib | both sexes combined |
| *Cd36* | -0.56 | 9.6E-05 | down | age | Gsn+ fib | both sexes combined |
| *Myl7* | -0.56 | 1.0E-17 | down | age | Gsn+ fib | both sexes combined |
| *Myom1* | -0.57 | 1.1E-03 | down | age | Gsn+ fib | both sexes combined |
| *Dsp* | -0.61 | 8.7E-15 | down | age | Gsn+ fib | both sexes combined |
| *Cmya5* | -0.66 | 2.7E-09 | down | age | Gsn+ fib | both sexes combined |
| *Tpm1* | -0.67 | 1.3E-77 | down | age | Gsn+ fib | both sexes combined |
| *Tnni3* | -0.68 | 7.1E-39 | down | age | Gsn+ fib | both sexes combined |
| *Pln* | -0.69 | 3.3E-38 | down | age | Gsn+ fib | both sexes combined |
| *Tnnt2* | -0.73 | 9.4E-61 | down | age | Gsn+ fib | both sexes combined |
| *Jarid2* | -0.77 | 4.0E-49 | down | age | Gsn+ fib | both sexes combined |
| *Atp2a2* | -0.77 | 2.9E-97 | down | age | Gsn+ fib | both sexes combined |
| *Filip1l* | -0.77 | 1.0E-64 | down | age | Gsn+ fib | both sexes combined |
| *Il31ra* | -0.81 | 1.0E-39 | down | age | Gsn+ fib | both sexes combined |
| *Gm15564* | -0.81 | 4.8E-27 | down | age | Gsn+ fib | both sexes combined |
| *Nppa* | -0.87 | 3.1E-62 | down | age | Gsn+ fib | both sexes combined |
| *Gphn* | -0.87 | 2.8E-233 | down | age | Gsn+ fib | both sexes combined |
| *Ryr2* | -0.88 | 1.3E-49 | down | age | Gsn+ fib | both sexes combined |
| *Myh6* | -0.95 | 1.8E-183 | down | age | Gsn+ fib | both sexes combined |
| *Camk1d* | -1.00 | 4.9E-275 | down | age | Gsn+ fib | both sexes combined |
| *Ttn* | -1.01 | 3.1E-218 | down | age | Gsn+ fib | both sexes combined |
| *Lars2* | -1.06 | 2.3E-290 | down | age | Gsn+ fib | both sexes combined |
| *Gm42418* | -1.17 | 0.0E+00 | down | age | Gsn+ fib | both sexes combined |
| *Cmss1* | -1.24 | 0.0E+00 | down | age | Gsn+ fib | both sexes combined |
| *AY036118* | -1.46 | 0.0E+00 | down | age | Gsn+ fib | both sexes combined |
| *Errfi1* | 1.444 | 5.46E-68 | up | age | Gsn+ fib | male |
| *Lsamp* | 1.149 | 1.36E-152 | up | age | Gsn+ fib | male |
| *Ebf2* | 1.078 | 1.28E-180 | up | age | Gsn+ fib | male |
| *Il1r1* | 1.070 | 7.94E-117 | up | age | Gsn+ fib | male |
| *Pcdh9* | 1.069 | 3.38E-174 | up | age | Gsn+ fib | male |
| *Sgcd* | 1.064 | 1.30E-136 | up | age | Gsn+ fib | male |
| *Adamtsl3* | 1.053 | 5.25E-134 | up | age | Gsn+ fib | male |
| *Rhoj* | 1.043 | 1.08E-149 | up | age | Gsn+ fib | male |
| *Mast4* | 1.020 | 3.27E-158 | up | age | Gsn+ fib | male |
| *Mir100hg* | 1.014 | 1.73E-173 | up | age | Gsn+ fib | male |
| *Negr1* | 1.013 | 6.45E-153 | up | age | Gsn+ fib | male |
| *Atp8b1* | 1.001 | 2.06E-138 | up | age | Gsn+ fib | male |
| *Egfr* | 0.990 | 4.47E-142 | up | age | Gsn+ fib | male |
| *Tenm3* | 0.983 | 2.03E-176 | up | age | Gsn+ fib | male |
| *Naaladl2* | 0.977 | 3.27E-165 | up | age | Gsn+ fib | male |
| *Gpc6* | 0.968 | 7.19E-158 | up | age | Gsn+ fib | male |
| *Gab2* | 0.958 | 3.95E-155 | up | age | Gsn+ fib | male |
| *Ngf* | 0.953 | 1.10E-120 | up | age | Gsn+ fib | male |
| *Cyth3* | 0.950 | 4.36E-135 | up | age | Gsn+ fib | male |
| *Lama2* | 0.946 | 7.99E-167 | up | age | Gsn+ fib | male |
| *Fndc1* | 0.933 | 4.73E-113 | up | age | Gsn+ fib | male |
| *Gfpt2* | 0.929 | 3.72E-126 | up | age | Gsn+ fib | male |
| *Zbtb7c* | 0.926 | 4.78E-124 | up | age | Gsn+ fib | male |
| *Adgrl3* | 0.922 | 2.55E-106 | up | age | Gsn+ fib | male |
| *Adamts12* | 0.920 | 8.60E-135 | up | age | Gsn+ fib | male |
| *C1qtnf7* | 0.905 | 4.81E-106 | up | age | Gsn+ fib | male |
| *Col14a1* | 0.901 | 4.19E-108 | up | age | Gsn+ fib | male |
| *Igfbp7* | 0.900 | 3.88E-137 | up | age | Gsn+ fib | male |
| *Plxdc2* | 0.896 | 3.70E-160 | up | age | Gsn+ fib | male |
| *Airn* | 0.890 | 4.34E-155 | up | age | Gsn+ fib | male |
| *Robo1* | 0.889 | 2.15E-127 | up | age | Gsn+ fib | male |
| *Zfpm2* | 0.884 | 6.35E-151 | up | age | Gsn+ fib | male |
| *Col5a3* | 0.881 | 1.46E-160 | up | age | Gsn+ fib | male |
| *Npas2* | 0.874 | 1.08E-127 | up | age | Gsn+ fib | male |
| *Anks1b* | 0.869 | 1.91E-102 | up | age | Gsn+ fib | male |
| *Rbms3* | 0.869 | 2.35E-155 | up | age | Gsn+ fib | male |
| *Lsp1* | 0.861 | 2.24E-177 | up | age | Gsn+ fib | male |
| *Smad3* | 0.860 | 1.39E-133 | up | age | Gsn+ fib | male |
| *Grik2* | 0.844 | 5.15E-98 | up | age | Gsn+ fib | male |
| *B3galt1* | 0.842 | 1.88E-148 | up | age | Gsn+ fib | male |
| *Cpq* | 0.841 | 1.32E-160 | up | age | Gsn+ fib | male |
| *Agmo* | 0.838 | 1.97E-127 | up | age | Gsn+ fib | male |
| *Slit2* | 0.834 | 1.87E-85 | up | age | Gsn+ fib | male |
| *Mir99ahg* | 0.832 | 3.05E-137 | up | age | Gsn+ fib | male |
| *Rora* | 0.831 | 3.72E-144 | up | age | Gsn+ fib | male |
| *Abca8a* | 0.821 | 7.67E-123 | up | age | Gsn+ fib | male |
| *Ebf1* | 0.817 | 5.38E-141 | up | age | Gsn+ fib | male |
| *Gulp1* | 0.817 | 8.52E-145 | up | age | Gsn+ fib | male |
| *Prickle2* | 0.807 | 1.15E-114 | up | age | Gsn+ fib | male |
| *Foxp2* | 0.804 | 3.08E-149 | up | age | Gsn+ fib | male |
| *Col8a1* | 0.801 | 5.69E-122 | up | age | Gsn+ fib | male |
| *Fbn1* | 0.799 | 5.93E-127 | up | age | Gsn+ fib | male |
| *Alpk1* | 0.798 | 1.25E-93 | up | age | Gsn+ fib | male |
| *Kcnt2* | 0.794 | 8.63E-142 | up | age | Gsn+ fib | male |
| *Wwox* | 0.790 | 3.13E-101 | up | age | Gsn+ fib | male |
| *Bnc2* | 0.789 | 6.29E-115 | up | age | Gsn+ fib | male |
| *Ghr* | 0.786 | 4.36E-127 | up | age | Gsn+ fib | male |
| *Thsd7a* | 0.786 | 1.70E-112 | up | age | Gsn+ fib | male |
| *Pde1a* | 0.784 | 5.54E-129 | up | age | Gsn+ fib | male |
| *Morrbid* | 0.781 | 5.89E-104 | up | age | Gsn+ fib | male |
| *Magi2* | 0.778 | 1.48E-100 | up | age | Gsn+ fib | male |
| *Medag* | 0.776 | 2.98E-124 | up | age | Gsn+ fib | male |
| *Dclk1* | 0.775 | 1.83E-107 | up | age | Gsn+ fib | male |
| *Sox5* | 0.771 | 3.84E-140 | up | age | Gsn+ fib | male |
| *Efna5* | 0.767 | 1.02E-103 | up | age | Gsn+ fib | male |
| *Itga5* | 0.765 | 4.98E-78 | up | age | Gsn+ fib | male |
| *Plcl1* | 0.762 | 1.45E-94 | up | age | Gsn+ fib | male |
| *Malat1* | 0.759 | 3.33E-179 | up | age | Gsn+ fib | male |
| *Rgs6* | 0.755 | 9.16E-50 | up | age | Gsn+ fib | male |
| *Lhfp* | 0.755 | 1.45E-138 | up | age | Gsn+ fib | male |
| *Celf2* | 0.754 | 5.49E-138 | up | age | Gsn+ fib | male |
| *Pard3b* | 0.751 | 3.57E-140 | up | age | Gsn+ fib | male |
| *Ldlrad4* | 0.751 | 1.54E-126 | up | age | Gsn+ fib | male |
| *Dlc1* | 0.747 | 8.29E-127 | up | age | Gsn+ fib | male |
| *Ext1* | 0.746 | 2.82E-131 | up | age | Gsn+ fib | male |
| *Pbx1* | 0.743 | 1.56E-133 | up | age | Gsn+ fib | male |
| *Cped1* | 0.737 | 3.04E-129 | up | age | Gsn+ fib | male |
| *Sik3* | 0.734 | 5.05E-140 | up | age | Gsn+ fib | male |
| *Psd3* | 0.733 | 8.40E-132 | up | age | Gsn+ fib | male |
| *Col5a2* | 0.733 | 5.96E-127 | up | age | Gsn+ fib | male |
| *Ank2* | 0.731 | 6.90E-131 | up | age | Gsn+ fib | male |
| *Gclc* | 0.726 | 2.47E-50 | up | age | Gsn+ fib | male |
| *B4galt1* | 0.720 | 4.00E-119 | up | age | Gsn+ fib | male |
| *Bicc1* | 0.719 | 4.10E-117 | up | age | Gsn+ fib | male |
| *Zeb1* | 0.714 | 8.29E-121 | up | age | Gsn+ fib | male |
| *Gucy1a2* | 0.711 | 6.70E-122 | up | age | Gsn+ fib | male |
| *Sh3rf3* | 0.705 | 1.19E-103 | up | age | Gsn+ fib | male |
| *Dchs2* | 0.705 | 1.80E-78 | up | age | Gsn+ fib | male |
| *Nfia* | 0.705 | 7.25E-125 | up | age | Gsn+ fib | male |
| *Vav3* | 0.701 | 9.11E-120 | up | age | Gsn+ fib | male |
| *Prkg1* | 0.701 | 6.20E-103 | up | age | Gsn+ fib | male |
| *Sdc2* | 0.697 | 1.18E-120 | up | age | Gsn+ fib | male |
| *Lncppara* | 0.697 | 2.04E-138 | up | age | Gsn+ fib | male |
| *Neat1* | 0.696 | 1.92E-132 | up | age | Gsn+ fib | male |
| *Fbxl7* | 0.685 | 8.52E-93 | up | age | Gsn+ fib | male |
| *Abi3bp* | 0.683 | 4.73E-115 | up | age | Gsn+ fib | male |
| *Cmklr1* | 0.683 | 1.34E-81 | up | age | Gsn+ fib | male |
| *Hivep2* | 0.682 | 3.27E-123 | up | age | Gsn+ fib | male |
| *Col4a1* | 0.678 | 1.70E-87 | up | age | Gsn+ fib | male |
| *Smoc2* | 0.673 | 8.97E-115 | up | age | Gsn+ fib | male |
| *Lama4* | 0.673 | 5.70E-118 | up | age | Gsn+ fib | male |
| *Ccn1* | 0.672 | 5.59E-55 | up | age | Gsn+ fib | male |
| *Col4a4* | 0.667 | 1.98E-102 | up | age | Gsn+ fib | male |
| *Fstl1* | 0.667 | 1.37E-113 | up | age | Gsn+ fib | male |
| *Antxr1* | 0.667 | 9.12E-141 | up | age | Gsn+ fib | male |
| *B4galt5* | 0.665 | 7.54E-87 | up | age | Gsn+ fib | male |
| *Nox4* | 0.663 | 6.47E-59 | up | age | Gsn+ fib | male |
| *Kcnq1ot1* | 0.661 | 1.99E-127 | up | age | Gsn+ fib | male |
| *Nkain2* | 0.661 | 7.11E-14 | up | age | Gsn+ fib | male |
| *Col4a2* | 0.660 | 1.48E-89 | up | age | Gsn+ fib | male |
| *Klf12* | 0.660 | 1.95E-117 | up | age | Gsn+ fib | male |
| *Grk5* | 0.660 | 4.37E-115 | up | age | Gsn+ fib | male |
| *Sh3d19* | 0.658 | 7.39E-111 | up | age | Gsn+ fib | male |
| *Col15a1* | 0.657 | 1.35E-98 | up | age | Gsn+ fib | male |
| *Abca6* | 0.653 | 1.05E-93 | up | age | Gsn+ fib | male |
| *Parva* | 0.653 | 1.32E-121 | up | age | Gsn+ fib | male |
| *Atrnl1* | 0.650 | 3.28E-118 | up | age | Gsn+ fib | male |
| *Fap* | 0.650 | 6.75E-81 | up | age | Gsn+ fib | male |
| *Kif13b* | 0.648 | 1.90E-142 | up | age | Gsn+ fib | male |
| *Plscr4* | 0.645 | 4.58E-99 | up | age | Gsn+ fib | male |
| *Itpr1* | 0.645 | 5.68E-118 | up | age | Gsn+ fib | male |
| *Pi16* | 0.645 | 1.42E-61 | up | age | Gsn+ fib | male |
| *Dock7* | 0.645 | 3.30E-136 | up | age | Gsn+ fib | male |
| *Sulf1* | 0.642 | 1.32E-106 | up | age | Gsn+ fib | male |
| *Plekhh2* | 0.642 | 1.16E-107 | up | age | Gsn+ fib | male |
| *Igf1r* | 0.641 | 8.24E-121 | up | age | Gsn+ fib | male |
| *Colec12* | 0.640 | 2.79E-104 | up | age | Gsn+ fib | male |
| *Ttc28* | 0.637 | 9.27E-119 | up | age | Gsn+ fib | male |
| *Creb3l2* | 0.637 | 7.34E-97 | up | age | Gsn+ fib | male |
| *Maml2* | 0.637 | 1.60E-113 | up | age | Gsn+ fib | male |
| *Bcl2l1* | 0.636 | 7.02E-76 | up | age | Gsn+ fib | male |
| *Pakap.1* | 0.634 | 1.06E-77 | up | age | Gsn+ fib | male |
| *Ptprd* | 0.632 | 4.07E-111 | up | age | Gsn+ fib | male |
| *Exoc4* | 0.632 | 4.97E-110 | up | age | Gsn+ fib | male |
| *Trps1* | 0.631 | 1.64E-112 | up | age | Gsn+ fib | male |
| *Sh3pxd2b* | 0.629 | 6.64E-94 | up | age | Gsn+ fib | male |
| *Asap1* | 0.627 | 2.01E-128 | up | age | Gsn+ fib | male |
| *Vcl* | 0.627 | 8.27E-99 | up | age | Gsn+ fib | male |
| *Stk32b* | 0.625 | 1.80E-65 | up | age | Gsn+ fib | male |
| *Frmd4a* | 0.624 | 4.09E-109 | up | age | Gsn+ fib | male |
| *Zfp521* | 0.622 | 2.25E-112 | up | age | Gsn+ fib | male |
| *Fam172a* | 0.622 | 4.18E-127 | up | age | Gsn+ fib | male |
| *Mecom* | 0.617 | 4.82E-108 | up | age | Gsn+ fib | male |
| *Adamts2* | 0.617 | 1.28E-124 | up | age | Gsn+ fib | male |
| *Ust* | 0.616 | 5.44E-114 | up | age | Gsn+ fib | male |
| *Prkn* | 0.616 | 1.40E-101 | up | age | Gsn+ fib | male |
| *Bcas3* | 0.615 | 3.48E-102 | up | age | Gsn+ fib | male |
| *Pcsk6* | 0.615 | 4.18E-114 | up | age | Gsn+ fib | male |
| *Pknox2* | 0.615 | 7.49E-90 | up | age | Gsn+ fib | male |
| *Nav1* | 0.615 | 1.96E-118 | up | age | Gsn+ fib | male |
| *Ube2e2* | 0.614 | 1.22E-108 | up | age | Gsn+ fib | male |
| *Adam19* | 0.613 | 4.85E-90 | up | age | Gsn+ fib | male |
| *Dnajc1* | 0.611 | 7.82E-128 | up | age | Gsn+ fib | male |
| *Akt3* | 0.610 | 2.72E-121 | up | age | Gsn+ fib | male |
| *Rhoq* | 0.610 | 2.23E-93 | up | age | Gsn+ fib | male |
| *Sobp* | 0.610 | 8.58E-106 | up | age | Gsn+ fib | male |
| *11-Sep* | 0.609 | 5.41E-114 | up | age | Gsn+ fib | male |
| *Arl15* | 0.608 | 5.89E-104 | up | age | Gsn+ fib | male |
| *Mill2* | 0.608 | 1.89E-122 | up | age | Gsn+ fib | male |
| *Mfap5* | 0.608 | 6.21E-86 | up | age | Gsn+ fib | male |
| *Unc5c* | 0.607 | 9.11E-66 | up | age | Gsn+ fib | male |
| *Abca8b* | 0.607 | 1.36E-88 | up | age | Gsn+ fib | male |
| *Zeb2* | 0.607 | 2.83E-100 | up | age | Gsn+ fib | male |
| *Large1* | 0.606 | 4.80E-109 | up | age | Gsn+ fib | male |
| *Lhfpl2* | 0.604 | 3.74E-75 | up | age | Gsn+ fib | male |
| *Ddr2* | 0.604 | 3.52E-124 | up | age | Gsn+ fib | male |
| *Samd4* | 0.602 | 1.86E-92 | up | age | Gsn+ fib | male |
| *Col3a1* | 0.600 | 2.98E-76 | up | age | Gsn+ fib | male |
| *2610035D17Rik* | 0.598 | 3.84E-82 | up | age | Gsn+ fib | male |
| *Mpp7* | 0.595 | 3.62E-34 | up | age | Gsn+ fib | male |
| *Ano6* | 0.593 | 1.41E-115 | up | age | Gsn+ fib | male |
| *Tcf7l1* | 0.593 | 4.05E-116 | up | age | Gsn+ fib | male |
| *Ubtd1* | 0.593 | 3.29E-66 | up | age | Gsn+ fib | male |
| *Lpar1* | 0.593 | 3.22E-96 | up | age | Gsn+ fib | male |
| *AC149090.1* | 0.591 | 8.47E-102 | up | age | Gsn+ fib | male |
| *Trhde* | 0.591 | 3.64E-66 | up | age | Gsn+ fib | male |
| *Glis3* | 0.591 | 3.81E-70 | up | age | Gsn+ fib | male |
| *Dock10* | 0.588 | 2.75E-89 | up | age | Gsn+ fib | male |
| *Plekha5* | 0.588 | 8.13E-118 | up | age | Gsn+ fib | male |
| *Fndc3a* | 0.585 | 1.67E-123 | up | age | Gsn+ fib | male |
| *Runx1t1* | 0.584 | 2.91E-89 | up | age | Gsn+ fib | male |
| *Luc7l2* | 0.583 | 9.03E-119 | up | age | Gsn+ fib | male |
| *Pvt1* | 0.582 | 2.63E-102 | up | age | Gsn+ fib | male |
| *Sntb2* | 0.580 | 2.68E-110 | up | age | Gsn+ fib | male |
| *Cdk14* | 0.580 | 1.23E-114 | up | age | Gsn+ fib | male |
| *Cblb* | 0.578 | 1.37E-106 | up | age | Gsn+ fib | male |
| *Fus* | 0.577 | 1.04E-123 | up | age | Gsn+ fib | male |
| *Sdk1* | 0.577 | 4.91E-37 | up | age | Gsn+ fib | male |
| *Cep112* | 0.576 | 2.99E-104 | up | age | Gsn+ fib | male |
| *Nedd4l* | 0.576 | 1.45E-98 | up | age | Gsn+ fib | male |
| *Svep1* | 0.574 | 4.99E-77 | up | age | Gsn+ fib | male |
| *Axl* | 0.574 | 1.89E-109 | up | age | Gsn+ fib | male |
| *Cd34* | 0.574 | 1.45E-100 | up | age | Gsn+ fib | male |
| *Rtn4* | 0.573 | 5.46E-103 | up | age | Gsn+ fib | male |
| *Ubash3b* | 0.573 | 1.38E-53 | up | age | Gsn+ fib | male |
| *Fgfr1* | 0.573 | 1.36E-117 | up | age | Gsn+ fib | male |
| *Itgbl1* | 0.571 | 6.48E-82 | up | age | Gsn+ fib | male |
| *Rftn1* | 0.571 | 2.12E-108 | up | age | Gsn+ fib | male |
| *Zbtb20* | 0.571 | 5.89E-126 | up | age | Gsn+ fib | male |
| *Adamts10* | 0.569 | 2.80E-107 | up | age | Gsn+ fib | male |
| *Vti1a* | 0.566 | 9.72E-105 | up | age | Gsn+ fib | male |
| *Pdzrn3* | 0.565 | 2.92E-77 | up | age | Gsn+ fib | male |
| *Sik2* | 0.565 | 2.21E-95 | up | age | Gsn+ fib | male |
| *Hivep3* | 0.562 | 9.15E-93 | up | age | Gsn+ fib | male |
| *Stk40* | 0.561 | 2.95E-82 | up | age | Gsn+ fib | male |
| *Gli3* | 0.561 | 2.27E-121 | up | age | Gsn+ fib | male |
| *Smg6* | 0.559 | 7.78E-109 | up | age | Gsn+ fib | male |
| *Pcolce2* | 0.558 | 1.34E-70 | up | age | Gsn+ fib | male |
| *Aopep* | 0.558 | 1.00E-108 | up | age | Gsn+ fib | male |
| *Cstf3* | 0.558 | 2.43E-95 | up | age | Gsn+ fib | male |
| *Arhgap42* | 0.558 | 1.07E-101 | up | age | Gsn+ fib | male |
| *Ncoa1* | 0.556 | 1.39E-118 | up | age | Gsn+ fib | male |
| *Man1a* | 0.556 | 3.97E-113 | up | age | Gsn+ fib | male |
| *Ptgis* | 0.556 | 3.12E-85 | up | age | Gsn+ fib | male |
| *Farp1* | 0.555 | 3.48E-107 | up | age | Gsn+ fib | male |
| *Rock2* | 0.554 | 5.96E-88 | up | age | Gsn+ fib | male |
| *5031425E22Rik* | 0.554 | 4.69E-97 | up | age | Gsn+ fib | male |
| *Lrrk1* | 0.554 | 4.72E-103 | up | age | Gsn+ fib | male |
| *Dock1* | 0.552 | 3.06E-124 | up | age | Gsn+ fib | male |
| *Gmds* | 0.552 | 9.24E-86 | up | age | Gsn+ fib | male |
| *Arhgap24* | 0.551 | 1.11E-88 | up | age | Gsn+ fib | male |
| *Gm32618* | 0.550 | 1.01E-80 | up | age | Gsn+ fib | male |
| *Cd55* | 0.550 | 1.84E-77 | up | age | Gsn+ fib | male |
| *Col6a2* | 0.549 | 1.53E-106 | up | age | Gsn+ fib | male |
| *Creb5* | 0.549 | 5.12E-79 | up | age | Gsn+ fib | male |
| *Gfra1* | 0.548 | 4.73E-59 | up | age | Gsn+ fib | male |
| *Arntl* | 0.547 | 8.00E-98 | up | age | Gsn+ fib | male |
| *Sec24d* | 0.547 | 2.57E-116 | up | age | Gsn+ fib | male |
| *Jak2* | 0.546 | 1.07E-77 | up | age | Gsn+ fib | male |
| *Rbm25* | 0.544 | 2.22E-127 | up | age | Gsn+ fib | male |
| *Phldb1* | 0.540 | 7.45E-108 | up | age | Gsn+ fib | male |
| *Arih1* | 0.537 | 1.12E-107 | up | age | Gsn+ fib | male |
| *Cntln* | 0.534 | 5.44E-87 | up | age | Gsn+ fib | male |
| *Ubtd2* | 0.533 | 2.33E-82 | up | age | Gsn+ fib | male |
| *Esyt2* | 0.533 | 5.74E-108 | up | age | Gsn+ fib | male |
| *Mtcl1* | 0.533 | 2.78E-41 | up | age | Gsn+ fib | male |
| *Sp100* | 0.530 | 8.41E-88 | up | age | Gsn+ fib | male |
| *Fam129b* | 0.530 | 2.87E-69 | up | age | Gsn+ fib | male |
| *Astn2* | 0.529 | 2.54E-43 | up | age | Gsn+ fib | male |
| *Xkr4* | 0.529 | 2.06E-31 | up | age | Gsn+ fib | male |
| *Ssbp2* | 0.529 | 3.45E-103 | up | age | Gsn+ fib | male |
| *Fmnl2* | 0.528 | 2.81E-101 | up | age | Gsn+ fib | male |
| *Ror2* | 0.527 | 2.56E-63 | up | age | Gsn+ fib | male |
| *Nbea* | 0.527 | 2.69E-114 | up | age | Gsn+ fib | male |
| *Zfand3* | 0.527 | 8.17E-111 | up | age | Gsn+ fib | male |
| *Traf5* | 0.526 | 7.24E-98 | up | age | Gsn+ fib | male |
| *St5* | 0.525 | 1.41E-88 | up | age | Gsn+ fib | male |
| *Fubp1* | 0.524 | 1.02E-126 | up | age | Gsn+ fib | male |
| *Plpp3* | 0.523 | 7.37E-56 | up | age | Gsn+ fib | male |
| *Rbms1* | 0.523 | 3.15E-90 | up | age | Gsn+ fib | male |
| *Atp9b* | 0.523 | 2.21E-117 | up | age | Gsn+ fib | male |
| *Exoc6b* | 0.522 | 5.27E-116 | up | age | Gsn+ fib | male |
| *Pdlim7* | 0.522 | 1.08E-94 | up | age | Gsn+ fib | male |
| *Pard3* | 0.522 | 1.38E-112 | up | age | Gsn+ fib | male |
| *Cald1* | 0.522 | 9.01E-99 | up | age | Gsn+ fib | male |
| *9530026P05Rik* | 0.520 | 1.17E-70 | up | age | Gsn+ fib | male |
| *Smyd3* | 0.519 | 7.28E-79 | up | age | Gsn+ fib | male |
| *Clmp* | 0.519 | 5.34E-88 | up | age | Gsn+ fib | male |
| *Arhgap32* | 0.518 | 1.71E-101 | up | age | Gsn+ fib | male |
| *Pdzd2* | 0.518 | 5.62E-101 | up | age | Gsn+ fib | male |
| *Plekha6* | 0.518 | 8.32E-98 | up | age | Gsn+ fib | male |
| *Kirrel* | 0.517 | 1.13E-106 | up | age | Gsn+ fib | male |
| *Nalcn* | 0.516 | 2.11E-71 | up | age | Gsn+ fib | male |
| *Prpf4b* | 0.516 | 7.58E-135 | up | age | Gsn+ fib | male |
| *Nfat5* | 0.516 | 1.89E-112 | up | age | Gsn+ fib | male |
| *Tcf4* | 0.515 | 8.55E-82 | up | age | Gsn+ fib | male |
| *9-Sep* | 0.515 | 4.34E-80 | up | age | Gsn+ fib | male |
| *Meis1* | 0.514 | 5.00E-97 | up | age | Gsn+ fib | male |
| *Dok6* | 0.514 | 1.32E-36 | up | age | Gsn+ fib | male |
| *Nfkb1* | 0.514 | 1.96E-105 | up | age | Gsn+ fib | male |
| *Prickle1* | 0.514 | 2.14E-64 | up | age | Gsn+ fib | male |
| *Srebf2* | 0.513 | 4.64E-92 | up | age | Gsn+ fib | male |
| *Sec24a* | 0.512 | 6.20E-91 | up | age | Gsn+ fib | male |
| *Gxylt2* | 0.512 | 1.40E-67 | up | age | Gsn+ fib | male |
| *Setbp1* | 0.512 | 1.16E-113 | up | age | Gsn+ fib | male |
| *Stat3* | 0.512 | 5.79E-98 | up | age | Gsn+ fib | male |
| *Rere* | 0.511 | 9.84E-109 | up | age | Gsn+ fib | male |
| *Mcc* | 0.511 | 5.99E-87 | up | age | Gsn+ fib | male |
| *Eif4g3* | 0.510 | 9.16E-112 | up | age | Gsn+ fib | male |
| *Spag9* | 0.509 | 1.97E-98 | up | age | Gsn+ fib | male |
| *St3gal4* | 0.507 | 3.12E-97 | up | age | Gsn+ fib | male |
| *Aff3* | 0.506 | 1.14E-78 | up | age | Gsn+ fib | male |
| *Stag1* | 0.506 | 1.08E-101 | up | age | Gsn+ fib | male |
| *Rsrc1* | 0.506 | 2.73E-106 | up | age | Gsn+ fib | male |
| *Col6a3* | 0.505 | 1.74E-98 | up | age | Gsn+ fib | male |
| *Sdccag8* | 0.505 | 9.59E-83 | up | age | Gsn+ fib | male |
| *Lncpint* | 0.505 | 1.28E-99 | up | age | Gsn+ fib | male |
| *Tcf25* | 0.503 | 2.35E-127 | up | age | Gsn+ fib | male |
| *Phf21a* | 0.503 | 3.23E-94 | up | age | Gsn+ fib | male |
| *Olfr1033* | 0.501 | 5.90E-96 | up | age | Gsn+ fib | male |
| *Chd9* | 0.501 | 3.38E-98 | up | age | Gsn+ fib | male |
| *Serpine1* | 0.501 | 6.36E-23 | up | age | Gsn+ fib | male |
| *Fer* | 0.500 | 7.39E-117 | up | age | Gsn+ fib | male |
| *Gli2* | 0.500 | 1.45E-84 | up | age | Gsn+ fib | male |
| *Nr3c2* | 0.499 | 6.08E-90 | up | age | Gsn+ fib | male |
| *Emp1* | 0.498 | 4.16E-92 | up | age | Gsn+ fib | male |
| *Auts2* | 0.497 | 4.49E-98 | up | age | Gsn+ fib | male |
| *Mgp* | 0.497 | 3.65E-39 | up | age | Gsn+ fib | male |
| *Dis3l2* | 0.497 | 3.94E-102 | up | age | Gsn+ fib | male |
| *Ppfia2* | 0.496 | 1.57E-58 | up | age | Gsn+ fib | male |
| *Son* | 0.496 | 6.92E-116 | up | age | Gsn+ fib | male |
| *Zfhx3* | 0.496 | 1.94E-107 | up | age | Gsn+ fib | male |
| *Atf7* | 0.495 | 8.71E-123 | up | age | Gsn+ fib | male |
| *Smurf1* | 0.495 | 8.10E-84 | up | age | Gsn+ fib | male |
| *Tcf12* | 0.495 | 4.45E-95 | up | age | Gsn+ fib | male |
| *Eml4* | 0.494 | 8.79E-79 | up | age | Gsn+ fib | male |
| *Ppard* | 0.493 | 2.96E-91 | up | age | Gsn+ fib | male |
| *Col5a1* | 0.493 | 2.07E-103 | up | age | Gsn+ fib | male |
| *Ror1* | 0.493 | 1.45E-94 | up | age | Gsn+ fib | male |
| *Ttyh2* | 0.492 | 5.99E-42 | up | age | Gsn+ fib | male |
| *Zswim6* | 0.490 | 2.77E-72 | up | age | Gsn+ fib | male |
| *Sparc* | 0.490 | 6.18E-82 | up | age | Gsn+ fib | male |
| *Meg3* | 0.489 | 8.86E-36 | up | age | Gsn+ fib | male |
| *Ly6a* | 0.489 | 4.22E-50 | up | age | Gsn+ fib | male |
| *Fto* | 0.489 | 6.57E-109 | up | age | Gsn+ fib | male |
| *Adgrd1* | 0.488 | 1.12E-51 | up | age | Gsn+ fib | male |
| *Plekhg1* | 0.487 | 1.26E-82 | up | age | Gsn+ fib | male |
| *St3gal1* | 0.487 | 8.31E-43 | up | age | Gsn+ fib | male |
| *Col27a1* | 0.487 | 1.36E-84 | up | age | Gsn+ fib | male |
| *Stk3* | 0.486 | 4.21E-106 | up | age | Gsn+ fib | male |
| *2210408F21Rik* | 0.485 | 3.33E-90 | up | age | Gsn+ fib | male |
| *Spaca6* | 0.484 | 1.00E-105 | up | age | Gsn+ fib | male |
| *Agap1* | 0.484 | 6.57E-94 | up | age | Gsn+ fib | male |
| *Baz2b* | 0.483 | 8.22E-115 | up | age | Gsn+ fib | male |
| *Bmp6* | 0.483 | 3.62E-78 | up | age | Gsn+ fib | male |
| *Akap13* | 0.483 | 9.10E-95 | up | age | Gsn+ fib | male |
| *Cmip* | 0.482 | 2.19E-94 | up | age | Gsn+ fib | male |
| *Tshz3* | 0.482 | 1.00E-90 | up | age | Gsn+ fib | male |
| *Pde3a* | 0.482 | 4.43E-89 | up | age | Gsn+ fib | male |
| *Nedd9* | 0.481 | 5.29E-72 | up | age | Gsn+ fib | male |
| *Rrbp1* | 0.481 | 5.06E-97 | up | age | Gsn+ fib | male |
| *Ncald* | 0.481 | 2.04E-72 | up | age | Gsn+ fib | male |
| *Dnm3* | 0.481 | 2.36E-79 | up | age | Gsn+ fib | male |
| *Ptpn12* | 0.480 | 5.73E-91 | up | age | Gsn+ fib | male |
| *Tcf7l2* | 0.480 | 2.16E-104 | up | age | Gsn+ fib | male |
| *Creb3l1* | 0.478 | 2.62E-53 | up | age | Gsn+ fib | male |
| *Cnot4* | 0.477 | 4.21E-107 | up | age | Gsn+ fib | male |
| *Stxbp1* | 0.477 | 9.13E-72 | up | age | Gsn+ fib | male |
| *Vcan* | 0.477 | 4.49E-85 | up | age | Gsn+ fib | male |
| *Smurf2* | 0.476 | 5.45E-112 | up | age | Gsn+ fib | male |
| *Slit3* | 0.475 | 5.20E-38 | up | age | Gsn+ fib | male |
| *Epb41l2* | 0.475 | 7.47E-110 | up | age | Gsn+ fib | male |
| *Adgre5* | 0.474 | 9.87E-75 | up | age | Gsn+ fib | male |
| *Setd5* | 0.473 | 8.44E-113 | up | age | Gsn+ fib | male |
| *Tnik* | 0.472 | 6.97E-82 | up | age | Gsn+ fib | male |
| *Fbln2* | 0.472 | 6.06E-69 | up | age | Gsn+ fib | male |
| *Lpp* | 0.471 | 8.91E-95 | up | age | Gsn+ fib | male |
| *Cox16* | 0.471 | 3.06E-76 | up | age | Gsn+ fib | male |
| *Fndc3b* | 0.471 | 3.15E-89 | up | age | Gsn+ fib | male |
| *Nsd3* | 0.471 | 1.94E-109 | up | age | Gsn+ fib | male |
| *Nktr* | 0.471 | 1.73E-110 | up | age | Gsn+ fib | male |
| *Gbf1* | 0.470 | 1.18E-104 | up | age | Gsn+ fib | male |
| *Sbf2* | 0.470 | 1.05E-105 | up | age | Gsn+ fib | male |
| *Phactr1* | 0.470 | 1.13E-83 | up | age | Gsn+ fib | male |
| *Ascc3* | 0.470 | 2.54E-103 | up | age | Gsn+ fib | male |
| *Fhit* | 0.469 | 2.00E-48 | up | age | Gsn+ fib | male |
| *Snx29* | 0.468 | 7.20E-95 | up | age | Gsn+ fib | male |
| *Frmd6* | 0.468 | 4.69E-88 | up | age | Gsn+ fib | male |
| *Zfc3h1* | 0.467 | 7.35E-115 | up | age | Gsn+ fib | male |
| *Babam2* | 0.467 | 7.00E-97 | up | age | Gsn+ fib | male |
| *Lima1* | 0.467 | 6.26E-119 | up | age | Gsn+ fib | male |
| *Adamts5* | 0.467 | 2.07E-63 | up | age | Gsn+ fib | male |
| *Cacna1a* | 0.467 | 2.92E-78 | up | age | Gsn+ fib | male |
| *Tgfbr3* | 0.467 | 6.64E-104 | up | age | Gsn+ fib | male |
| *Gm36535* | 0.466 | 4.13E-81 | up | age | Gsn+ fib | male |
| *Xdh* | 0.466 | 6.36E-77 | up | age | Gsn+ fib | male |
| *Islr* | 0.465 | 1.78E-33 | up | age | Gsn+ fib | male |
| *Chsy3* | 0.464 | 3.82E-39 | up | age | Gsn+ fib | male |
| *Pid1* | 0.462 | 4.46E-79 | up | age | Gsn+ fib | male |
| *Rlf* | 0.462 | 1.00E-95 | up | age | Gsn+ fib | male |
| *Cdkal1* | 0.461 | 3.36E-93 | up | age | Gsn+ fib | male |
| *Ston2* | 0.460 | 3.21E-65 | up | age | Gsn+ fib | male |
| *Gm12216* | 0.460 | 2.37E-74 | up | age | Gsn+ fib | male |
| *Trio* | 0.460 | 3.28E-108 | up | age | Gsn+ fib | male |
| *Sec24b* | 0.459 | 1.51E-100 | up | age | Gsn+ fib | male |
| *Fyn* | 0.459 | 2.76E-96 | up | age | Gsn+ fib | male |
| *Loxl1* | 0.459 | 3.49E-86 | up | age | Gsn+ fib | male |
| *Lrch3* | 0.458 | 2.25E-108 | up | age | Gsn+ fib | male |
| *Lims1* | 0.457 | 9.08E-92 | up | age | Gsn+ fib | male |
| *Disp1* | 0.457 | 1.04E-65 | up | age | Gsn+ fib | male |
| *Srsf11* | 0.456 | 3.34E-131 | up | age | Gsn+ fib | male |
| *Aldh1a2* | 0.456 | 3.84E-29 | up | age | Gsn+ fib | male |
| *Pld1* | 0.456 | 7.70E-90 | up | age | Gsn+ fib | male |
| *Meis2* | 0.455 | 1.31E-89 | up | age | Gsn+ fib | male |
| *Atxn7l1* | 0.454 | 4.88E-74 | up | age | Gsn+ fib | male |
| *Mycbp2* | 0.454 | 8.20E-102 | up | age | Gsn+ fib | male |
| *Col4a3* | 0.453 | 1.66E-50 | up | age | Gsn+ fib | male |
| *Abi1* | 0.452 | 1.27E-77 | up | age | Gsn+ fib | male |
| *Rgma* | 0.452 | 6.81E-66 | up | age | Gsn+ fib | male |
| *Kmt5b* | 0.451 | 5.91E-93 | up | age | Gsn+ fib | male |
| *Fars2* | 0.451 | 1.83E-91 | up | age | Gsn+ fib | male |
| *Fbxl17* | 0.450 | 1.07E-91 | up | age | Gsn+ fib | male |
| *Wsb1* | 0.448 | 2.65E-117 | up | age | Gsn+ fib | male |
| *Tnrc6c* | 0.448 | 7.00E-115 | up | age | Gsn+ fib | male |
| *Tmcc1* | 0.448 | 2.95E-97 | up | age | Gsn+ fib | male |
| *Ltbp4* | 0.447 | 1.88E-72 | up | age | Gsn+ fib | male |
| *Mcu* | 0.446 | 5.82E-88 | up | age | Gsn+ fib | male |
| *Lamc1* | 0.446 | 1.39E-67 | up | age | Gsn+ fib | male |
| *Rtn3* | 0.446 | 1.07E-94 | up | age | Gsn+ fib | male |
| *Rbm6* | 0.446 | 9.33E-108 | up | age | Gsn+ fib | male |
| *Kansl1* | 0.445 | 7.36E-106 | up | age | Gsn+ fib | male |
| *Arid1b* | 0.445 | 9.66E-115 | up | age | Gsn+ fib | male |
| *Spred1* | 0.445 | 1.32E-106 | up | age | Gsn+ fib | male |
| *Abtb2* | 0.445 | 2.37E-39 | up | age | Gsn+ fib | male |
| *Gas7* | 0.443 | 1.22E-57 | up | age | Gsn+ fib | male |
| *Zcchc7* | 0.443 | 1.35E-95 | up | age | Gsn+ fib | male |
| *Prdm5* | 0.443 | 8.16E-86 | up | age | Gsn+ fib | male |
| *Rsu1* | 0.443 | 2.44E-103 | up | age | Gsn+ fib | male |
| *Afap1* | 0.442 | 1.09E-90 | up | age | Gsn+ fib | male |
| *Sash1* | 0.441 | 1.48E-94 | up | age | Gsn+ fib | male |
| *Etv6* | 0.439 | 2.77E-97 | up | age | Gsn+ fib | male |
| *4632427E13Rik* | 0.438 | 3.71E-98 | up | age | Gsn+ fib | male |
| *Anxa2* | 0.437 | 2.56E-84 | up | age | Gsn+ fib | male |
| *Steap3* | 0.436 | 5.22E-67 | up | age | Gsn+ fib | male |
| *Lum* | 0.435 | 1.77E-72 | up | age | Gsn+ fib | male |
| *Zfp948* | 0.435 | 5.31E-58 | up | age | Gsn+ fib | male |
| *Trappc9* | 0.434 | 1.82E-73 | up | age | Gsn+ fib | male |
| *Rbm39* | 0.434 | 4.02E-105 | up | age | Gsn+ fib | male |
| *Ccdc171* | 0.434 | 4.93E-62 | up | age | Gsn+ fib | male |
| *Dpysl3* | 0.433 | 2.79E-69 | up | age | Gsn+ fib | male |
| *Mbd5* | 0.431 | 1.83E-97 | up | age | Gsn+ fib | male |
| *Dcbld2* | 0.431 | 2.70E-96 | up | age | Gsn+ fib | male |
| *Pcolce* | 0.429 | 5.63E-76 | up | age | Gsn+ fib | male |
| *Clec3b* | 0.429 | 4.12E-52 | up | age | Gsn+ fib | male |
| *Rab8b* | 0.429 | 1.93E-83 | up | age | Gsn+ fib | male |
| *Xrcc4* | 0.429 | 6.62E-47 | up | age | Gsn+ fib | male |
| *Naalad2* | 0.428 | 4.10E-81 | up | age | Gsn+ fib | male |
| *Fnbp1* | 0.428 | 1.34E-108 | up | age | Gsn+ fib | male |
| *Nmt2* | 0.427 | 9.81E-56 | up | age | Gsn+ fib | male |
| *Mical2* | 0.427 | 9.74E-69 | up | age | Gsn+ fib | male |
| *Galnt17* | 0.427 | 1.19E-49 | up | age | Gsn+ fib | male |
| *Rnf150* | 0.427 | 5.88E-73 | up | age | Gsn+ fib | male |
| *Tgfbr2* | 0.427 | 9.10E-88 | up | age | Gsn+ fib | male |
| *Aspa* | 0.426 | 7.02E-58 | up | age | Gsn+ fib | male |
| *Sptlc2* | 0.426 | 1.28E-77 | up | age | Gsn+ fib | male |
| *Il16* | 0.425 | 3.26E-75 | up | age | Gsn+ fib | male |
| *Jmjd1c* | 0.424 | 9.67E-104 | up | age | Gsn+ fib | male |
| *Bmper* | 0.424 | 1.17E-67 | up | age | Gsn+ fib | male |
| *Cdh19* | 0.424 | 9.83E-76 | up | age | Gsn+ fib | male |
| *Rbfox2* | 0.424 | 1.50E-96 | up | age | Gsn+ fib | male |
| *3-Mar* | 0.424 | 2.66E-60 | up | age | Gsn+ fib | male |
| *Dennd1a* | 0.423 | 2.27E-111 | up | age | Gsn+ fib | male |
| *Nup98* | 0.423 | 2.70E-88 | up | age | Gsn+ fib | male |
| *Runx2* | 0.423 | 1.91E-54 | up | age | Gsn+ fib | male |
| *Junb* | 0.422 | 1.01E-49 | up | age | Gsn+ fib | male |
| *Ccnl1* | 0.422 | 3.62E-98 | up | age | Gsn+ fib | male |
| *Apbb1ip* | 0.421 | 1.84E-62 | up | age | Gsn+ fib | male |
| *Nf1* | 0.420 | 5.27E-99 | up | age | Gsn+ fib | male |
| *Pitpna* | 0.420 | 7.52E-78 | up | age | Gsn+ fib | male |
| *Cradd* | 0.420 | 4.10E-56 | up | age | Gsn+ fib | male |
| *Apba1* | 0.419 | 1.48E-68 | up | age | Gsn+ fib | male |
| *Arglu1* | 0.419 | 3.14E-124 | up | age | Gsn+ fib | male |
| *Stard9* | 0.417 | 3.96E-90 | up | age | Gsn+ fib | male |
| *Tet2* | 0.417 | 4.37E-90 | up | age | Gsn+ fib | male |
| *Tsc22d2* | 0.415 | 2.95E-86 | up | age | Gsn+ fib | male |
| *Pla2r1* | 0.415 | 1.67E-65 | up | age | Gsn+ fib | male |
| *Ccnl2* | 0.414 | 7.24E-101 | up | age | Gsn+ fib | male |
| *Rfx3* | 0.414 | 5.76E-72 | up | age | Gsn+ fib | male |
| *Mrc2* | 0.414 | 3.58E-85 | up | age | Gsn+ fib | male |
| *Slc7a11* | 0.413 | 6.66E-39 | up | age | Gsn+ fib | male |
| *Stx8* | 0.412 | 2.25E-63 | up | age | Gsn+ fib | male |
| *Micu1* | 0.411 | 2.90E-72 | up | age | Gsn+ fib | male |
| *Ggnbp2* | 0.411 | 9.36E-99 | up | age | Gsn+ fib | male |
| *Zfr* | 0.411 | 9.56E-91 | up | age | Gsn+ fib | male |
| *Arsj* | 0.411 | 9.79E-50 | up | age | Gsn+ fib | male |
| *Prex2* | 0.410 | 4.95E-84 | up | age | Gsn+ fib | male |
| *Tra2a* | 0.410 | 1.92E-100 | up | age | Gsn+ fib | male |
| *Camk2d* | 0.410 | 2.17E-93 | up | age | Gsn+ fib | male |
| *Ap2b1* | 0.410 | 2.06E-83 | up | age | Gsn+ fib | male |
| *Bach2* | 0.410 | 7.04E-64 | up | age | Gsn+ fib | male |
| *Pde7b* | 0.410 | 6.74E-52 | up | age | Gsn+ fib | male |
| *Mllt3* | 0.407 | 1.62E-73 | up | age | Gsn+ fib | male |
| *Arnt* | 0.407 | 5.01E-103 | up | age | Gsn+ fib | male |
| *Gpatch8* | 0.407 | 5.01E-101 | up | age | Gsn+ fib | male |
| *Angpt1* | 0.407 | 8.90E-59 | up | age | Gsn+ fib | male |
| *Tshz2* | 0.407 | 3.95E-78 | up | age | Gsn+ fib | male |
| *Myo9b* | 0.407 | 1.58E-82 | up | age | Gsn+ fib | male |
| *1110051M20Rik* | 0.407 | 3.09E-70 | up | age | Gsn+ fib | male |
| *Loxl2* | 0.406 | 2.49E-55 | up | age | Gsn+ fib | male |
| *Vps13b* | 0.406 | 1.81E-106 | up | age | Gsn+ fib | male |
| *Pmepa1* | 0.406 | 2.62E-75 | up | age | Gsn+ fib | male |
| *Fnbp4* | 0.406 | 5.29E-103 | up | age | Gsn+ fib | male |
| *Dip2c* | 0.405 | 9.09E-87 | up | age | Gsn+ fib | male |
| *Ahnak2* | 0.405 | 6.56E-76 | up | age | Gsn+ fib | male |
| *Chd2* | 0.405 | 7.88E-95 | up | age | Gsn+ fib | male |
| *Fam114a1* | 0.405 | 1.85E-94 | up | age | Gsn+ fib | male |
| *Rai1* | 0.405 | 1.22E-74 | up | age | Gsn+ fib | male |
| *Ppl* | 0.404 | 6.20E-31 | up | age | Gsn+ fib | male |
| *Ldlr* | 0.404 | 2.00E-64 | up | age | Gsn+ fib | male |
| *Sestd1* | 0.404 | 7.38E-77 | up | age | Gsn+ fib | male |
| *Dnm1* | 0.404 | 1.96E-76 | up | age | Gsn+ fib | male |
| *Syt17* | 0.402 | 2.78E-54 | up | age | Gsn+ fib | male |
| *Csf1* | 0.402 | 3.57E-64 | up | age | Gsn+ fib | male |
| *Luzp1* | 0.402 | 1.68E-88 | up | age | Gsn+ fib | male |
| *Map2k5* | 0.402 | 1.81E-80 | up | age | Gsn+ fib | male |
| *Smg7* | 0.402 | 2.20E-79 | up | age | Gsn+ fib | male |
| *Crtc3* | 0.401 | 3.87E-86 | up | age | Gsn+ fib | male |
| *Nova1* | 0.401 | 9.43E-53 | up | age | Gsn+ fib | male |
| *Npas3* | 0.401 | 2.30E-37 | up | age | Gsn+ fib | male |
| *Wdpcp* | 0.401 | 9.75E-57 | up | age | Gsn+ fib | male |
| *Dcun1d3* | 0.401 | 4.59E-59 | up | age | Gsn+ fib | male |
| *Xrn2* | 0.401 | 5.47E-83 | up | age | Gsn+ fib | male |
| *Tmlhe* | 0.401 | 1.03E-68 | up | age | Gsn+ fib | male |
| *Tram2* | 0.400 | 1.58E-67 | up | age | Gsn+ fib | male |
| *Strn3* | 0.400 | 2.34E-87 | up | age | Gsn+ fib | male |
| *Fermt2* | 0.399 | 4.33E-88 | up | age | Gsn+ fib | male |
| *Gls* | 0.399 | 2.30E-102 | up | age | Gsn+ fib | male |
| *Arhgap10* | 0.399 | 3.49E-82 | up | age | Gsn+ fib | male |
| *Ankrd11* | 0.398 | 1.71E-99 | up | age | Gsn+ fib | male |
| *Glcci1* | 0.398 | 3.31E-65 | up | age | Gsn+ fib | male |
| *Enpp1* | 0.398 | 5.36E-40 | up | age | Gsn+ fib | male |
| *Mmp16* | 0.398 | 5.39E-42 | up | age | Gsn+ fib | male |
| *Elp4* | 0.397 | 5.77E-50 | up | age | Gsn+ fib | male |
| *Tns3* | 0.397 | 4.14E-72 | up | age | Gsn+ fib | male |
| *Ebf3* | 0.397 | 7.98E-82 | up | age | Gsn+ fib | male |
| *Sfswap* | 0.397 | 1.60E-90 | up | age | Gsn+ fib | male |
| *Mndal* | 0.396 | 1.58E-67 | up | age | Gsn+ fib | male |
| *Stxbp6* | 0.396 | 2.00E-62 | up | age | Gsn+ fib | male |
| *Osmr* | 0.396 | 5.42E-82 | up | age | Gsn+ fib | male |
| *Snd1* | 0.396 | 5.98E-88 | up | age | Gsn+ fib | male |
| *Scfd2* | 0.396 | 1.39E-56 | up | age | Gsn+ fib | male |
| *Snrnp70* | 0.396 | 1.49E-105 | up | age | Gsn+ fib | male |
| *Abca9* | 0.395 | 9.14E-63 | up | age | Gsn+ fib | male |
| *9930021J03Rik* | 0.395 | 3.60E-80 | up | age | Gsn+ fib | male |
| *Ptprm* | 0.394 | 5.95E-90 | up | age | Gsn+ fib | male |
| *Peak1* | 0.393 | 5.69E-78 | up | age | Gsn+ fib | male |
| *Cacna2d1* | 0.393 | 4.14E-84 | up | age | Gsn+ fib | male |
| *Myo1e* | 0.393 | 1.06E-75 | up | age | Gsn+ fib | male |
| *Msrb3* | 0.393 | 1.18E-61 | up | age | Gsn+ fib | male |
| *Pnisr* | 0.392 | 1.15E-110 | up | age | Gsn+ fib | male |
| *Pdgfra* | 0.392 | 3.11E-75 | up | age | Gsn+ fib | male |
| *Crebbp* | 0.392 | 3.37E-94 | up | age | Gsn+ fib | male |
| *Brwd1* | 0.392 | 1.07E-91 | up | age | Gsn+ fib | male |
| *Tln2* | 0.392 | 2.87E-77 | up | age | Gsn+ fib | male |
| *Camkmt* | 0.391 | 1.16E-43 | up | age | Gsn+ fib | male |
| *Rbm5* | 0.391 | 1.72E-107 | up | age | Gsn+ fib | male |
| *Col1a1* | 0.391 | 1.22E-60 | up | age | Gsn+ fib | male |
| *Fam219a* | 0.391 | 4.69E-52 | up | age | Gsn+ fib | male |
| *Xylt1* | 0.390 | 1.51E-66 | up | age | Gsn+ fib | male |
| *Mpdz* | 0.390 | 2.26E-91 | up | age | Gsn+ fib | male |
| *Dlg1* | 0.390 | 3.94E-96 | up | age | Gsn+ fib | male |
| *Gse1* | 0.389 | 3.30E-67 | up | age | Gsn+ fib | male |
| *Ambra1* | 0.389 | 8.17E-110 | up | age | Gsn+ fib | male |
| *E130307A14Rik* | 0.388 | 5.88E-62 | up | age | Gsn+ fib | male |
| *Usp32* | 0.388 | 4.17E-75 | up | age | Gsn+ fib | male |
| *Metap1d* | 0.387 | 1.17E-69 | up | age | Gsn+ fib | male |
| *Capn2* | 0.387 | 2.31E-80 | up | age | Gsn+ fib | male |
| *Umad1* | 0.387 | 2.47E-72 | up | age | Gsn+ fib | male |
| *Col1a2* | 0.387 | 1.30E-52 | up | age | Gsn+ fib | male |
| *Btaf1* | 0.387 | 1.65E-77 | up | age | Gsn+ fib | male |
| *Aven* | 0.386 | 3.31E-42 | up | age | Gsn+ fib | male |
| *Cdk6* | 0.386 | 3.87E-67 | up | age | Gsn+ fib | male |
| *Nckap5* | 0.383 | 8.51E-57 | up | age | Gsn+ fib | male |
| *Dnm3os* | 0.383 | 2.54E-60 | up | age | Gsn+ fib | male |
| *Syn2* | 0.382 | 7.44E-36 | up | age | Gsn+ fib | male |
| *Vegfc* | 0.382 | 9.84E-61 | up | age | Gsn+ fib | male |
| *Jak1* | 0.382 | 2.35E-89 | up | age | Gsn+ fib | male |
| *Itga11* | 0.382 | 6.65E-59 | up | age | Gsn+ fib | male |
| *Crppa* | 0.382 | 1.70E-44 | up | age | Gsn+ fib | male |
| *Arhgef40* | 0.381 | 5.57E-77 | up | age | Gsn+ fib | male |
| *Ncoa2* | 0.381 | 3.29E-96 | up | age | Gsn+ fib | male |
| *Slc1a3* | 0.381 | 9.56E-56 | up | age | Gsn+ fib | male |
| *Prkca* | 0.381 | 5.91E-81 | up | age | Gsn+ fib | male |
| *Ankrd17* | 0.380 | 2.94E-83 | up | age | Gsn+ fib | male |
| *Upp2* | 0.380 | 3.20E-46 | up | age | Gsn+ fib | male |
| *Ltbp1* | 0.380 | 1.08E-62 | up | age | Gsn+ fib | male |
| *Srrm2* | 0.379 | 3.07E-96 | up | age | Gsn+ fib | male |
| *Cpne8* | 0.378 | 5.82E-79 | up | age | Gsn+ fib | male |
| *Tbc1d5* | 0.378 | 2.24E-97 | up | age | Gsn+ fib | male |
| *Tgfbr1* | 0.378 | 5.83E-73 | up | age | Gsn+ fib | male |
| *Rbm26* | 0.378 | 1.93E-100 | up | age | Gsn+ fib | male |
| *Rps6ka5* | 0.378 | 2.75E-62 | up | age | Gsn+ fib | male |
| *Flrt2* | 0.377 | 4.08E-33 | up | age | Gsn+ fib | male |
| *Nfkbiz* | 0.377 | 1.05E-54 | up | age | Gsn+ fib | male |
| *AW554918* | 0.377 | 3.18E-70 | up | age | Gsn+ fib | male |
| *Itgb8* | 0.377 | 4.55E-53 | up | age | Gsn+ fib | male |
| *Cdk12* | 0.377 | 1.53E-101 | up | age | Gsn+ fib | male |
| *Ccbe1* | 0.376 | 3.89E-34 | up | age | Gsn+ fib | male |
| *Cuedc1* | 0.376 | 1.16E-86 | up | age | Gsn+ fib | male |
| *Akap12* | 0.376 | 1.70E-69 | up | age | Gsn+ fib | male |
| *Scaper* | 0.375 | 1.31E-87 | up | age | Gsn+ fib | male |
| *Trerf1* | 0.375 | 3.02E-74 | up | age | Gsn+ fib | male |
| *Arfgap1* | 0.375 | 4.91E-78 | up | age | Gsn+ fib | male |
| *Pbx3* | 0.375 | 8.86E-44 | up | age | Gsn+ fib | male |
| *Gm26917* | 0.374 | 1.24E-101 | up | age | Gsn+ fib | male |
| *Nsmce2* | 0.374 | 1.28E-84 | up | age | Gsn+ fib | male |
| *Rcor1* | 0.374 | 2.44E-82 | up | age | Gsn+ fib | male |
| *Elmo1* | 0.374 | 3.01E-88 | up | age | Gsn+ fib | male |
| *Myh9* | 0.374 | 7.80E-72 | up | age | Gsn+ fib | male |
| *Maml3* | 0.373 | 1.06E-57 | up | age | Gsn+ fib | male |
| *Ptpra* | 0.373 | 9.58E-91 | up | age | Gsn+ fib | male |
| *Tspan9* | 0.373 | 6.09E-88 | up | age | Gsn+ fib | male |
| *Fam13b* | 0.373 | 2.73E-96 | up | age | Gsn+ fib | male |
| *Rnf216* | 0.373 | 3.96E-77 | up | age | Gsn+ fib | male |
| *Pibf1* | 0.373 | 6.27E-76 | up | age | Gsn+ fib | male |
| *Mxra7* | 0.372 | 1.88E-66 | up | age | Gsn+ fib | male |
| *Il6ra* | 0.372 | 4.77E-45 | up | age | Gsn+ fib | male |
| *Ptk2* | 0.371 | 4.44E-91 | up | age | Gsn+ fib | male |
| *Homer1* | 0.371 | 3.03E-36 | up | age | Gsn+ fib | male |
| *Btbd9* | 0.370 | 2.54E-80 | up | age | Gsn+ fib | male |
| *Clip2* | 0.370 | 4.49E-68 | up | age | Gsn+ fib | male |
| *Trak1* | 0.370 | 1.02E-91 | up | age | Gsn+ fib | male |
| *Kcmf1* | 0.369 | 1.26E-98 | up | age | Gsn+ fib | male |
| *Rgs17* | 0.369 | 2.82E-37 | up | age | Gsn+ fib | male |
| *Elf1* | 0.368 | 1.17E-78 | up | age | Gsn+ fib | male |
| *Dnm2* | 0.368 | 1.60E-83 | up | age | Gsn+ fib | male |
| *Stxbp5* | 0.368 | 2.20E-95 | up | age | Gsn+ fib | male |
| *Heg1* | 0.367 | 1.32E-58 | up | age | Gsn+ fib | male |
| *Myof* | 0.367 | 2.30E-71 | up | age | Gsn+ fib | male |
| *Map3k14* | 0.367 | 1.45E-56 | up | age | Gsn+ fib | male |
| *Dlg2* | 0.367 | 1.70E-52 | up | age | Gsn+ fib | male |
| *Scmh1* | 0.367 | 2.50E-83 | up | age | Gsn+ fib | male |
| *Ifi203* | 0.367 | 1.87E-70 | up | age | Gsn+ fib | male |
| *Myo9a* | 0.367 | 1.46E-90 | up | age | Gsn+ fib | male |
| *Ubn1* | 0.367 | 8.99E-88 | up | age | Gsn+ fib | male |
| *Chic2* | 0.366 | 5.97E-84 | up | age | Gsn+ fib | male |
| *Adcy7* | 0.366 | 2.73E-49 | up | age | Gsn+ fib | male |
| *Cdk11b* | 0.365 | 2.52E-75 | up | age | Gsn+ fib | male |
| *Mbnl2* | 0.365 | 2.31E-79 | up | age | Gsn+ fib | male |
| *Fam193a* | 0.365 | 3.15E-101 | up | age | Gsn+ fib | male |
| *Zfp407* | 0.365 | 6.14E-72 | up | age | Gsn+ fib | male |
| *Srek1* | 0.364 | 6.06E-92 | up | age | Gsn+ fib | male |
| *Fancc* | 0.363 | 4.75E-64 | up | age | Gsn+ fib | male |
| *Nckap1* | 0.362 | 1.53E-92 | up | age | Gsn+ fib | male |
| *Gata4* | 0.362 | 7.65E-93 | up | age | Gsn+ fib | male |
| *Pdgfrb* | 0.361 | 1.43E-59 | up | age | Gsn+ fib | male |
| *Lrrc8d* | 0.361 | 3.83E-64 | up | age | Gsn+ fib | male |
| *Gpr107* | 0.361 | 1.25E-69 | up | age | Gsn+ fib | male |
| *Hnrnpdl* | 0.361 | 8.45E-96 | up | age | Gsn+ fib | male |
| *Gnao1* | 0.361 | 2.62E-69 | up | age | Gsn+ fib | male |
| *Parp8* | 0.360 | 1.79E-75 | up | age | Gsn+ fib | male |
| *Tmbim1* | 0.360 | 3.09E-74 | up | age | Gsn+ fib | male |
| *Armh3* | 0.360 | 1.08E-54 | up | age | Gsn+ fib | male |
| *Pnn* | 0.360 | 1.49E-95 | up | age | Gsn+ fib | male |
| *Rsrp1* | 0.360 | 1.98E-97 | up | age | Gsn+ fib | male |
| *Fbxw11* | 0.359 | 3.62E-82 | up | age | Gsn+ fib | male |
| *Thada* | 0.358 | 2.56E-87 | up | age | Gsn+ fib | male |
| *Zbtb38* | 0.358 | 4.09E-78 | up | age | Gsn+ fib | male |
| *Arid2* | 0.358 | 1.32E-74 | up | age | Gsn+ fib | male |
| *Asap3* | 0.358 | 1.44E-46 | up | age | Gsn+ fib | male |
| *Rabgap1l* | 0.358 | 2.07E-86 | up | age | Gsn+ fib | male |
| *Arhgap21* | 0.358 | 9.76E-87 | up | age | Gsn+ fib | male |
| *Dync2h1* | 0.358 | 3.02E-66 | up | age | Gsn+ fib | male |
| *Desi2* | 0.358 | 1.09E-70 | up | age | Gsn+ fib | male |
| *Paxbp1* | 0.358 | 1.18E-87 | up | age | Gsn+ fib | male |
| *Spry2* | 0.357 | 8.66E-61 | up | age | Gsn+ fib | male |
| *Zc3h6* | 0.357 | 5.30E-57 | up | age | Gsn+ fib | male |
| *Prkce* | 0.357 | 5.01E-85 | up | age | Gsn+ fib | male |
| *Pde4d* | -0.356 | 0.00638801 | down | age | Gsn+ fib | male |
| *Zfp106* | -0.365 | 4.61E-13 | down | age | Gsn+ fib | male |
| *Sdha* | -0.366 | 4.55E-05 | down | age | Gsn+ fib | male |
| *Ivns1abp* | -0.369 | 5.56E-06 | down | age | Gsn+ fib | male |
| *Oip5os1* | -0.378 | 3.12E-06 | down | age | Gsn+ fib | male |
| *Ndufs1* | -0.398 | 0.0001816 | down | age | Gsn+ fib | male |
| *Etfa* | -0.427 | 7.50E-06 | down | age | Gsn+ fib | male |
| *Xirp2* | -0.440 | 9.77E-06 | down | age | Gsn+ fib | male |
| *Fabp4* | -0.449 | 0.00119867 | down | age | Gsn+ fib | male |
| *Tpt1* | -0.514 | 0.00052388 | down | age | Gsn+ fib | male |
| *Lmod2* | -0.526 | 0.00175059 | down | age | Gsn+ fib | male |
| *Myl7* | -0.534 | 1.24E-10 | down | age | Gsn+ fib | male |
| *Npr3* | -0.540 | 0.0291415 | down | age | Gsn+ fib | male |
| *Cdk8* | -0.576 | 4.81E-05 | down | age | Gsn+ fib | male |
| *Mybpc3* | -0.590 | 2.18E-05 | down | age | Gsn+ fib | male |
| *Actn2* | -0.605 | 0.00906794 | down | age | Gsn+ fib | male |
| *Ckm* | -0.610 | 0.01418476 | down | age | Gsn+ fib | male |
| *Gja1* | -0.611 | 0.01740594 | down | age | Gsn+ fib | male |
| *Tnnc1* | -0.643 | 0.00424717 | down | age | Gsn+ fib | male |
| *Eno3* | -0.649 | 5.94E-05 | down | age | Gsn+ fib | male |
| *Cryab* | -0.682 | 8.81E-07 | down | age | Gsn+ fib | male |
| *Slc25a4* | -0.692 | 1.02E-18 | down | age | Gsn+ fib | male |
| *Lmo7* | -0.693 | 0.03743155 | down | age | Gsn+ fib | male |
| *Uqcr11* | -0.693 | 0.00093157 | down | age | Gsn+ fib | male |
| *Myl3* | -0.697 | 1.17E-29 | down | age | Gsn+ fib | male |
| *Myom1* | -0.700 | 7.95E-05 | down | age | Gsn+ fib | male |
| *Tmtc2* | -0.711 | 3.14E-06 | down | age | Gsn+ fib | male |
| *Igfbp5* | -0.716 | 6.91E-05 | down | age | Gsn+ fib | male |
| *Cox7a1* | -0.725 | 0.00046817 | down | age | Gsn+ fib | male |
| *Txlnb* | -0.736 | 3.69E-07 | down | age | Gsn+ fib | male |
| *Lpl* | -0.747 | 2.83E-33 | down | age | Gsn+ fib | male |
| *Cox8b* | -0.756 | 0.0001761 | down | age | Gsn+ fib | male |
| *Ckmt2* | -0.772 | 3.10E-13 | down | age | Gsn+ fib | male |
| *Idh2* | -0.772 | 7.91E-06 | down | age | Gsn+ fib | male |
| *Hrc* | -0.788 | 4.21E-13 | down | age | Gsn+ fib | male |
| *Atp5b* | -0.792 | 1.04E-09 | down | age | Gsn+ fib | male |
| *Atp5g3* | -0.811 | 1.39E-12 | down | age | Gsn+ fib | male |
| *Sorbs2* | -0.816 | 1.60E-08 | down | age | Gsn+ fib | male |
| *Mb* | -0.823 | 5.02E-67 | down | age | Gsn+ fib | male |
| *Ndufa4* | -0.826 | 1.21E-15 | down | age | Gsn+ fib | male |
| *C7* | -0.828 | 1.88E-07 | down | age | Gsn+ fib | male |
| *Cox6a2* | -0.830 | 1.05E-26 | down | age | Gsn+ fib | male |
| *Acadm* | -0.837 | 3.92E-07 | down | age | Gsn+ fib | male |
| *Atp5a1* | -0.838 | 2.61E-09 | down | age | Gsn+ fib | male |
| *Dsp* | -0.855 | 5.74E-20 | down | age | Gsn+ fib | male |
| *Cox6c* | -0.856 | 1.26E-09 | down | age | Gsn+ fib | male |
| *Cmya5* | -0.863 | 1.09E-08 | down | age | Gsn+ fib | male |
| *Fabp3* | -0.884 | 1.71E-42 | down | age | Gsn+ fib | male |
| *Actc1* | -0.906 | 4.77E-79 | down | age | Gsn+ fib | male |
| *Jarid2* | -0.919 | 8.99E-27 | down | age | Gsn+ fib | male |
| *Myl2* | -0.961 | 8.72E-77 | down | age | Gsn+ fib | male |
| *Hbb-bs* | -0.964 | 5.47E-234 | down | age | Gsn+ fib | male |
| *Filip1l* | -0.972 | 2.21E-48 | down | age | Gsn+ fib | male |
| *Hba-a2* | -0.977 | 2.10E-67 | down | age | Gsn+ fib | male |
| *Cd36* | -0.994 | 8.14E-38 | down | age | Gsn+ fib | male |
| *Hbb-bt* | -1.000 | 2.79E-56 | down | age | Gsn+ fib | male |
| *Gm15564* | -1.038 | 4.22E-25 | down | age | Gsn+ fib | male |
| *Nppa* | -1.041 | 3.03E-72 | down | age | Gsn+ fib | male |
| *Tnni3* | -1.049 | 2.19E-84 | down | age | Gsn+ fib | male |
| *Hba-a1* | -1.057 | 6.73E-109 | down | age | Gsn+ fib | male |
| *Gphn* | -1.058 | 2.09E-249 | down | age | Gsn+ fib | male |
| *Tpm1* | -1.067 | 6.36E-172 | down | age | Gsn+ fib | male |
| *Pln* | -1.068 | 8.89E-82 | down | age | Gsn+ fib | male |
| *Atp2a2* | -1.085 | 3.27E-126 | down | age | Gsn+ fib | male |
| *Tnnt2* | -1.097 | 4.85E-127 | down | age | Gsn+ fib | male |
| *Ryr2* | -1.165 | 1.53E-49 | down | age | Gsn+ fib | male |
| *Il31ra* | -1.168 | 2.86E-52 | down | age | Gsn+ fib | male |
| *Ttn* | -1.179 | 3.31E-279 | down | age | Gsn+ fib | male |
| *Camk1d* | -1.209 | 0 | down | age | Gsn+ fib | male |
| *Myh6* | -1.263 | 2.38E-259 | down | age | Gsn+ fib | male |
| *Lars2* | -1.360 | 0 | down | age | Gsn+ fib | male |
| *Gm42418* | -1.541 | 0 | down | age | Gsn+ fib | male |
| *Cmss1* | -1.571 | 0 | down | age | Gsn+ fib | male |
| *AY036118* | -1.975 | 0 | down | age | Gsn+ fib | male |
| *Hba-a2* | 1.85 | 1.56E-85 | up | age | Gsn+ fib | female |
| *Hba-a1* | 1.82 | 1.70E-93 | up | age | Gsn+ fib | female |
| *Hbb-bs* | 1.52 | 5.57E-112 | up | age | Gsn+ fib | female |
| *Hbb-bt* | 1.05 | 3.29E-45 | up | age | Gsn+ fib | female |
| *C7* | 1.02 | 1.12E-35 | up | age | Gsn+ fib | female |
| *Dcn* | 0.65 | 3.33E-57 | up | age | Gsn+ fib | female |
| *Ftl1* | 0.65 | 3.85E-24 | up | age | Gsn+ fib | female |
| *Igfbp6* | 0.63 | 1.42E-18 | up | age | Gsn+ fib | female |
| *Cfh* | 0.61 | 1.09E-36 | up | age | Gsn+ fib | female |
| *H2-D1* | 0.60 | 1.29E-18 | up | age | Gsn+ fib | female |
| *Postn* | 0.58 | 3.42E-06 | up | age | Gsn+ fib | female |
| *Serping1* | 0.57 | 5.59E-26 | up | age | Gsn+ fib | female |
| *Rnase4* | 0.55 | 2.18E-12 | up | age | Gsn+ fib | female |
| *Mgp* | 0.54 | 2.49E-26 | up | age | Gsn+ fib | female |
| *Adamts5* | 0.53 | 1.48E-18 | up | age | Gsn+ fib | female |
| *Abca6* | 0.53 | 1.58E-14 | up | age | Gsn+ fib | female |
| *Hsd11b1* | 0.53 | 1.40E-20 | up | age | Gsn+ fib | female |
| *Cd9* | 0.51 | 4.38E-12 | up | age | Gsn+ fib | female |
| *B2m* | 0.51 | 1.09E-16 | up | age | Gsn+ fib | female |
| *C3* | 0.51 | 1.04E-12 | up | age | Gsn+ fib | female |
| *Cp* | 0.51 | 8.53E-18 | up | age | Gsn+ fib | female |
| *Ly6a* | 0.50 | 7.95E-20 | up | age | Gsn+ fib | female |
| *AC149090.1* | 0.50 | 1.62E-18 | up | age | Gsn+ fib | female |
| *Ctsl* | 0.50 | 4.02E-14 | up | age | Gsn+ fib | female |
| *Sod3* | 0.49 | 1.49E-10 | up | age | Gsn+ fib | female |
| *Pcolce2* | 0.49 | 2.47E-17 | up | age | Gsn+ fib | female |
| *H2-K1* | 0.48 | 3.24E-06 | up | age | Gsn+ fib | female |
| *Fabp4* | 0.48 | 7.58E-35 | up | age | Gsn+ fib | female |
| *Crip1* | 0.48 | 8.37E-10 | up | age | Gsn+ fib | female |
| *Ifitm3* | 0.48 | 1.05E-14 | up | age | Gsn+ fib | female |
| *Ubb* | 0.47 | 7.94E-16 | up | age | Gsn+ fib | female |
| *Lvrn* | 0.47 | 2.20E-11 | up | age | Gsn+ fib | female |
| *Cygb* | 0.46 | 1.62E-14 | up | age | Gsn+ fib | female |
| *Aspn* | 0.46 | 1.14E-08 | up | age | Gsn+ fib | female |
| *Apoe* | 0.46 | 4.15E-14 | up | age | Gsn+ fib | female |
| *Abca8a* | 0.46 | 4.86E-15 | up | age | Gsn+ fib | female |
| *Ltbp4* | 0.45 | 5.34E-11 | up | age | Gsn+ fib | female |
| *Rplp1* | 0.45 | 3.31E-17 | up | age | Gsn+ fib | female |
| *Ace* | 0.44 | 4.63E-07 | up | age | Gsn+ fib | female |
| *Fbln2* | 0.44 | 6.60E-10 | up | age | Gsn+ fib | female |
| *Mmp14* | 0.44 | 0.000112102 | up | age | Gsn+ fib | female |
| *Rps5* | 0.43 | 1.02E-09 | up | age | Gsn+ fib | female |
| *Islr* | 0.43 | 3.60E-07 | up | age | Gsn+ fib | female |
| *Rps8* | 0.43 | 5.71E-15 | up | age | Gsn+ fib | female |
| *Pi16* | 0.42 | 1.11E-08 | up | age | Gsn+ fib | female |
| *Sparc* | 0.42 | 9.85E-11 | up | age | Gsn+ fib | female |
| *Efemp1* | 0.42 | 1.21E-15 | up | age | Gsn+ fib | female |
| *Abca9* | 0.42 | 2.67E-11 | up | age | Gsn+ fib | female |
| *Rarres2* | 0.42 | 9.68E-07 | up | age | Gsn+ fib | female |
| *Clec3b* | 0.42 | 4.10E-11 | up | age | Gsn+ fib | female |
| *Timp2* | 0.42 | 3.38E-10 | up | age | Gsn+ fib | female |
| *Fmo2* | 0.41 | 1.31E-12 | up | age | Gsn+ fib | female |
| *Rpl29* | 0.41 | 2.12E-07 | up | age | Gsn+ fib | female |
| *Rpl18a* | 0.41 | 6.96E-15 | up | age | Gsn+ fib | female |
| *Col8a1* | 0.41 | 8.31E-14 | up | age | Gsn+ fib | female |
| *Rsrp1* | 0.41 | 3.55E-15 | up | age | Gsn+ fib | female |
| *Slco2b1* | 0.41 | 6.40E-11 | up | age | Gsn+ fib | female |
| *Cyp27a1* | 0.41 | 1.62E-12 | up | age | Gsn+ fib | female |
| *Serpinf1* | 0.41 | 1.35E-05 | up | age | Gsn+ fib | female |
| *Ccdc80* | 0.41 | 1.65E-10 | up | age | Gsn+ fib | female |
| *Ppib* | 0.40 | 0.037092283 | up | age | Gsn+ fib | female |
| *Nox4* | 0.40 | 8.63E-05 | up | age | Gsn+ fib | female |
| *Rps10* | 0.40 | 3.74E-08 | up | age | Gsn+ fib | female |
| *Txnip* | 0.40 | 1.51E-19 | up | age | Gsn+ fib | female |
| *Aebp1* | 0.40 | 3.61E-09 | up | age | Gsn+ fib | female |
| *Cst3* | 0.40 | 7.33E-24 | up | age | Gsn+ fib | female |
| *Ecm1* | 0.40 | 2.81E-05 | up | age | Gsn+ fib | female |
| *Enpp2* | 0.39 | 1.98E-07 | up | age | Gsn+ fib | female |
| *Pla1a* | 0.39 | 3.36E-10 | up | age | Gsn+ fib | female |
| *Rpl6* | 0.39 | 3.66E-07 | up | age | Gsn+ fib | female |
| *Rpl38* | 0.39 | 2.24E-07 | up | age | Gsn+ fib | female |
| *Lamp1* | 0.39 | 1.15E-08 | up | age | Gsn+ fib | female |
| *Rpl37* | 0.38 | 0.000154194 | up | age | Gsn+ fib | female |
| *Pcolce* | 0.38 | 7.37E-07 | up | age | Gsn+ fib | female |
| *Rps16* | 0.38 | 3.86E-10 | up | age | Gsn+ fib | female |
| *Prelp* | 0.38 | 3.17E-06 | up | age | Gsn+ fib | female |
| *Selenop* | 0.38 | 1.61E-17 | up | age | Gsn+ fib | female |
| *Ankrd1* | 0.38 | 9.89E-12 | up | age | Gsn+ fib | female |
| *Ahnak2* | 0.38 | 7.28E-18 | up | age | Gsn+ fib | female |
| *Rps20* | 0.38 | 8.90E-09 | up | age | Gsn+ fib | female |
| *Rps24* | 0.37 | 2.01E-08 | up | age | Gsn+ fib | female |
| *Ly6e* | 0.37 | 3.07E-09 | up | age | Gsn+ fib | female |
| *Sfrp1* | 0.37 | 1.32E-08 | up | age | Gsn+ fib | female |
| *Aldh1a1* | 0.37 | 1.55E-07 | up | age | Gsn+ fib | female |
| *Ifi27l2a* | 0.37 | 9.24E-07 | up | age | Gsn+ fib | female |
| *Sparcl1* | 0.36 | 6.70E-14 | up | age | Gsn+ fib | female |
| *Sqstm1* | 0.36 | 2.21E-09 | up | age | Gsn+ fib | female |
| *Rps3* | 0.36 | 1.19E-07 | up | age | Gsn+ fib | female |
| *Npr3* | 0.36 | 7.26E-11 | up | age | Gsn+ fib | female |
| *Hsp90b1* | 0.36 | 9.07E-05 | up | age | Gsn+ fib | female |
| *Dpep1* | 0.36 | 4.91E-07 | up | age | Gsn+ fib | female |
| *Nid1* | 0.36 | 3.81E-09 | up | age | Gsn+ fib | female |
| *Kcnk2* | 0.36 | 0.0001119 | up | age | Gsn+ fib | female |
| *Cd248* | 0.36 | 0.000121546 | up | age | Gsn+ fib | female |
| *Taco1* | -0.38 | 4.58E-07 | down | age | Gsn+ fib | female |
| *Ubash3b* | -0.42 | 6.65E-05 | down | age | Gsn+ fib | female |
| *Ryr2* | -0.47 | 0.011296515 | down | age | Gsn+ fib | female |
| *Gm15564* | -0.48 | 0.011522983 | down | age | Gsn+ fib | female |
| *Filip1l* | -0.48 | 1.91E-13 | down | age | Gsn+ fib | female |
| *Egr1* | -0.49 | 0.006934311 | down | age | Gsn+ fib | female |
| *Jarid2* | -0.55 | 2.40E-18 | down | age | Gsn+ fib | female |
| *Myl7* | -0.59 | 0.002051076 | down | age | Gsn+ fib | female |
| *Gm42418* | -0.59 | 6.10E-30 | down | age | Gsn+ fib | female |
| *Gphn* | -0.60 | 1.39E-25 | down | age | Gsn+ fib | female |
| *Lars2* | -0.61 | 1.31E-19 | down | age | Gsn+ fib | female |
| *AY036118* | -0.63 | 2.45E-16 | down | age | Gsn+ fib | female |
| *Nppa* | -0.64 | 0.00567402 | down | age | Gsn+ fib | female |
| *Camk1d* | -0.69 | 1.23E-29 | down | age | Gsn+ fib | female |
| *Cmss1* | -0.74 | 1.17E-22 | down | age | Gsn+ fib | female |
| *Ttn* | -0.77 | 1.33E-06 | down | age | Gsn+ fib | female |
| *Rgs6* | -0.89 | 1.97E-20 | down | age | Gsn+ fib | female |

**Table S9.** Top ten enriched Gene Ontology terms for Biological Process (GO BP) based on age up- and down-regulated DEGs in *Gsn^+^* fibroblasts; snRNA-seq, both sex combined, adj.p-value<0.05, |log2FC| >0.36

| **Term** | **Overlap** | **Adj.P.value** | **Genes** | **GeneRatio** | **comparison** | **cell_type** | **direction** |
| --- | --- | --- | --- | --- | --- | --- | --- |
| External Encapsulating Structure Organization (GO:0045229) | 10/111 | 5.73E-06 | ADAMTS5;SMOC2;POSTN;COL3A1;COL4A2;COL4A1;COL14A1;COL5A3;COL5A2;COL8A1 | 0.06 | Aging | Gsn+ Fibroblast | up, both |
| Extracellular Structure Organization (GO:0043062) | 10/111 | 5.73E-06 | ADAMTS5;SMOC2;POSTN;COL3A1;COL4A2;COL4A1;COL14A1;COL5A3;COL5A2;COL8A1 | 0.06 | Aging | Gsn+ Fibroblast | up, both |
| Extracellular Matrix Organization (GO:0030198) | 12/182 | 5.73E-06 | ADAMTS5;SMOC2;POSTN;COL3A1;COL4A2;COL4A1;COL14A1;LUM;COL5A3;COL5A2;  COL8A1;LOXL1 | 0.08 | Aging | Gsn+ Fibroblast | up, both |
| Regulation of Angiogenesis (GO:0045765) | 11/211 | 1.84E-04 | SMOC2;SFRP1;ECM1;SP100;SPARC;PTGIS;COL4A2;RHOJ;SULF1;CD34;DCN | 0.07 | Aging | Gsn+ Fibroblast | up, both |
| Collagen Fibril Organization (GO:0030199) | 6/45 | 2.37E-04 | COL3A1;COL14A1;LUM;COL5A3;COL5A2;  LOXL1 | 0.04 | Aging | Gsn+ Fibroblast | up, both |
| Eye Morphogenesis (GO:0048592) | 4/23 | 4.38E-03 | MFAP5;TENM3;COL5A2;FBN1 | 0.03 | Aging | Gsn+ Fibroblast | up, both |
| Regulation of Vascular Endothelial Growth Factor Signaling Pathway (GO:1900746) | 4/25 | 5.30E-03 | SMOC2;ITGA5;DCN;ROBO1 | 0.03 | Aging | Gsn+ Fibroblast | up, both |
| Regulation of Endothelial Cell Migration (GO:0010594) | 6/88 | 7.50E-03 | SMOC2;SP100;SPARC;RHOJ;PLPP3;DCN | 0.04 | Aging | Gsn+ Fibroblast | up, both |
| Positive Regulation of Vascular Endothelial Growth Factor Signaling Pathway (GO:1900748) | 3/11 | 7.73E-03 | SMOC2;ITGA5;ROBO1 | 0.02 | Aging | Gsn+ Fibroblast | up, both |
| Negative Regulation of Cell Migration (GO:0030336) | 8/184 | 9.03E-03 | SFRP1;SP100;COL3A1;MAGI2;SULF1;VCL;  DCN;ROBO1 | 0.05 | Aging | Gsn+ Fibroblast | up, both |
| Heart Contraction (GO:0060047) | 9/41 | 1.33E-14 | MYL4;RYR2;ACTC1;MYL7;TNNT2;MYL2;  TPM1;TNNI3;MYH6 | 0.24 | Aging | Gsn+ Fibroblast | down, both |
| Cardiac Muscle Contraction (GO:0060048) | 7/34 | 4.71E-11 | MYL4;RYR2;MYL2;TNNT2;TPM1;TNNI3;MYH6 | 0.19 | Aging | Gsn+ Fibroblast | down, both |
| Regulation of Heart Contraction (GO:0008016) | 8/70 | 7.90E-11 | RYR2;BMP10;PLN;TNNT2;TPM1;HRC;  ATP2A2;MYH6 | 0.22 | Aging | Gsn+ Fibroblast | down, both |
| Ventricular Cardiac Muscle Tissue Development (GO:0003229) | 6/24 | 3.84E-10 | BMP10;MYL2;TNNT2;TPM1;TNNI3;MYH6 | 0.16 | Aging | Gsn+ Fibroblast | down, both |
| Striated Muscle Contraction (GO:0006941) | 7/55 | 6.91E-10 | MYL4;RYR2;TNNT2;MYL2;TPM1;TNNI3;MYH6 | 0.19 | Aging | Gsn+ Fibroblast | down, both |
| Cardiac Muscle Tissue Morphogenesis (GO:0055008) | 6/30 | 1.12E-09 | BMP10;MYL2;TNNT2;TPM1;TNNI3;MYH6 | 0.16 | Aging | Gsn+ Fibroblast | down, both |
| Ventricular Cardiac Muscle Tissue Morphogenesis (GO:0055010) | 6/33 | 1.68E-09 | RYR2;MYL2;TNNT2;TPM1;TNNI3;MYH6 | 0.16 | Aging | Gsn+ Fibroblast | down, both |
| Positive Regulation of Heart Rate (GO:0010460) | 5/14 | 1.68E-09 | RYR2;TPM1;NPPA;HRC;ATP2A2 | 0.14 | Aging | Gsn+ Fibroblast | down, both |
| Regulation of Cardiac Muscle Contraction (GO:0055117) | 6/34 | 1.68E-09 | RYR2;BMP10;PLN;NPPA;ATP2A2;TNNI3 | 0.16 | Aging | Gsn+ Fibroblast | down, both |
| Myofibril Assembly (GO:0030239) | 6/51 | 1.88E-08 | MYOM1;BMP10;TNNT2;MYL2;TPM1;MYH6 | 0.16 | Aging | Gsn+ Fibroblast | down, both |

**Table S10.** Aging differentially expressed genes in endothelial cells; snRNA-seq; 6- vs 24-month-old C57BL/6J mice, n=3 per age/sex; adj.p-value<0.05, |log2FC| >0.36

| **Gene** | **avg_log2FC** | **p_val_adj** | **Up/Down DEG** | **group** | **cell** | **Both sexes, males, females** |
| --- | --- | --- | --- | --- | --- | --- |
| *Cfh* | 0.48 | 1.7E-63 | up | age | endothelial | both sexes combined |
| *Lsamp* | 0.40 | 2.1E-05 | up | age | endothelial | both sexes combined |
| *Mgp* | 0.39 | 4.5E-64 | up | age | endothelial | both sexes combined |
| *Kcnt2* | 0.38 | 2.0E-32 | up | age | endothelial | both sexes combined |
| *Ly6a* | 0.36 | 2.2E-56 | up | age | endothelial | both sexes combined |
| *Nppa* | -0.36 | 1.0E-38 | down | age | endothelial | both sexes combined |
| *Atp2a2* | -0.37 | 6.3E-61 | down | age | endothelial | both sexes combined |
| *Gphn* | -0.37 | 1.4E-72 | down | age | endothelial | both sexes combined |
| *Zbtb16* | -0.37 | 1.8E-69 | down | age | endothelial | both sexes combined |
| *Kcnd2* | -0.37 | 1.2E-11 | down | age | endothelial | both sexes combined |
| *Gm15564* | -0.40 | 1.8E-40 | down | age | endothelial | both sexes combined |
| *Il31ra* | -0.44 | 2.8E-69 | down | age | endothelial | both sexes combined |
| *Filip1l* | -0.45 | 6.8E-73 | down | age | endothelial | both sexes combined |
| *Ryr2* | -0.47 | 9.3E-76 | down | age | endothelial | both sexes combined |
| *Myh6* | -0.51 | 5.1E-145 | down | age | endothelial | both sexes combined |
| *Ttn* | -0.65 | 1.4E-190 | down | age | endothelial | both sexes combined |
| *Camk1d* | -0.69 | 4.9E-228 | down | age | endothelial | both sexes combined |
| *Lars2* | -0.73 | 2.1E-283 | down | age | endothelial | both sexes combined |
| *Gm42418* | -0.78 | 0.0E+00 | down | age | endothelial | both sexes combined |
| *Cmss1* | -0.84 | 5.7E-277 | down | age | endothelial | both sexes combined |
| *AY036118* | -0.97 | 0.0E+00 | down | age | endothelial | both sexes combined |
| *Iigp1* | 0.65 | 4.70E-23 | up | age | endothelial | male |
| *Nav3* | 0.51 | 1.09E-19 | up | age | endothelial | male |
| *Cdh13* | 0.48 | 1.07E-76 | up | age | endothelial | male |
| *Dlg2* | 0.48 | 1.62E-16 | up | age | endothelial | male |
| *Gm4951* | 0.46 | 5.44E-38 | up | age | endothelial | male |
| *Adam12* | 0.44 | 4.51E-30 | up | age | endothelial | male |
| *Btnl9* | 0.43 | 2.40E-27 | up | age | endothelial | male |
| *Robo2* | 0.43 | 5.69E-40 | up | age | endothelial | male |
| *Itgbl1* | 0.42 | 5.70E-05 | up | age | endothelial | male |
| *Ly6a* | 0.41 | 8.77E-44 | up | age | endothelial | male |
| *Epha7* | 0.40 | 2.22E-07 | up | age | endothelial | male |
| *Arhgap18* | 0.39 | 2.04E-35 | up | age | endothelial | male |
| *Kcnt2* | 0.38 | 1.48E-02 | up | age | endothelial | male |
| *Pdgfd* | 0.37 | 1.57E-42 | up | age | endothelial | male |
| *Ly6e* | 0.36 | 1.80E-45 | up | age | endothelial | male |
| *Gbp4* | 0.36 | 5.78E-18 | up | age | endothelial | male |
| *Gulp1* | 0.36 | 9.59E-51 | up | age | endothelial | male |
| *Dcn* | -0.36 | 1.00E-24 | down | age | endothelial | male |
| *Ckm* | -0.36 | 2.02E-22 | down | age | endothelial | male |
| *Ech1* | -0.36 | 4.42E-15 | down | age | endothelial | male |
| *Ogdh* | -0.36 | 4.35E-09 | down | age | endothelial | male |
| *Mdh1* | -0.36 | 1.86E-24 | down | age | endothelial | male |
| *Cox5b* | -0.37 | 9.71E-20 | down | age | endothelial | male |
| *Eno3* | -0.37 | 3.60E-21 | down | age | endothelial | male |
| *Atp5k* | -0.37 | 3.13E-23 | down | age | endothelial | male |
| *Uqcr11* | -0.38 | 1.80E-22 | down | age | endothelial | male |
| *Acadl* | -0.38 | 5.87E-14 | down | age | endothelial | male |
| *Atp5md* | -0.38 | 8.24E-14 | down | age | endothelial | male |
| *Myoz2* | -0.38 | 3.02E-23 | down | age | endothelial | male |
| *Actn2* | -0.38 | 2.15E-26 | down | age | endothelial | male |
| *Uqcrfs1* | -0.38 | 1.37E-12 | down | age | endothelial | male |
| *Ndufa6* | -0.38 | 4.93E-11 | down | age | endothelial | male |
| *Atp5f1* | -0.38 | 8.04E-10 | down | age | endothelial | male |
| *Hexb* | -0.38 | 3.86E-08 | down | age | endothelial | male |
| *Csrp3* | -0.39 | 7.35E-18 | down | age | endothelial | male |
| *Lama2* | -0.39 | 8.07E-30 | down | age | endothelial | male |
| *Atp5e* | -0.39 | 8.76E-17 | down | age | endothelial | male |
| *Aco2* | -0.39 | 4.63E-15 | down | age | endothelial | male |
| *Abca8a* | -0.39 | 9.46E-19 | down | age | endothelial | male |
| *Ogn* | -0.39 | 2.00E-23 | down | age | endothelial | male |
| *Nexn* | -0.40 | 2.95E-21 | down | age | endothelial | male |
| *D830005E20Rik* | -0.40 | 8.96E-25 | down | age | endothelial | male |
| *Oxct1* | -0.40 | 3.18E-22 | down | age | endothelial | male |
| *Cmya5* | -0.40 | 1.39E-31 | down | age | endothelial | male |
| *Txlnb* | -0.40 | 7.41E-26 | down | age | endothelial | male |
| *Rcan2* | -0.40 | 3.08E-21 | down | age | endothelial | male |
| *Hadhb* | -0.40 | 2.99E-23 | down | age | endothelial | male |
| *Hopx* | -0.41 | 4.61E-45 | down | age | endothelial | male |
| *Dsp* | -0.41 | 2.18E-44 | down | age | endothelial | male |
| *Pde4dip* | -0.42 | 5.86E-17 | down | age | endothelial | male |
| *Cox4i1* | -0.42 | 3.96E-26 | down | age | endothelial | male |
| *Atp5h* | -0.42 | 5.37E-21 | down | age | endothelial | male |
| *Cdh2* | -0.42 | 1.28E-23 | down | age | endothelial | male |
| *Atp5g1* | -0.43 | 1.21E-21 | down | age | endothelial | male |
| *Jarid2* | -0.43 | 2.25E-28 | down | age | endothelial | male |
| *Ldb3* | -0.43 | 2.18E-36 | down | age | endothelial | male |
| *Atp5l* | -0.43 | 1.80E-19 | down | age | endothelial | male |
| *Uqcrq* | -0.44 | 8.53E-19 | down | age | endothelial | male |
| *Zbtb16* | -0.44 | 1.67E-38 | down | age | endothelial | male |
| *Lmod2* | -0.44 | 7.27E-41 | down | age | endothelial | male |
| *Cox5a* | -0.44 | 4.29E-21 | down | age | endothelial | male |
| *Cox7c* | -0.45 | 8.34E-34 | down | age | endothelial | male |
| *Gphn* | -0.45 | 4.04E-54 | down | age | endothelial | male |
| *Cox7a1* | -0.45 | 1.04E-40 | down | age | endothelial | male |
| *Slc8a1* | -0.45 | 1.24E-27 | down | age | endothelial | male |
| *Idh2* | -0.46 | 4.73E-33 | down | age | endothelial | male |
| *Fhod3* | -0.47 | 9.84E-30 | down | age | endothelial | male |
| *Hrc* | -0.47 | 1.63E-47 | down | age | endothelial | male |
| *Cox8b* | -0.48 | 1.68E-51 | down | age | endothelial | male |
| *Ctnna3* | -0.48 | 1.03E-10 | down | age | endothelial | male |
| *Cryab* | -0.49 | 8.89E-35 | down | age | endothelial | male |
| *Cacna1c* | -0.49 | 5.00E-17 | down | age | endothelial | male |
| *Myom1* | -0.50 | 1.12E-36 | down | age | endothelial | male |
| *Atp5a1* | -0.50 | 8.45E-35 | down | age | endothelial | male |
| *Hspa8* | -0.50 | 1.50E-49 | down | age | endothelial | male |
| *Cacnb2* | -0.51 | 7.81E-31 | down | age | endothelial | male |
| *Trdn* | -0.52 | 7.10E-48 | down | age | endothelial | male |
| *Nppa* | -0.52 | 7.21E-63 | down | age | endothelial | male |
| *Slc25a4* | -0.52 | 6.96E-57 | down | age | endothelial | male |
| *Cdk8* | -0.52 | 2.75E-28 | down | age | endothelial | male |
| *Kcnd2* | -0.54 | 4.02E-29 | down | age | endothelial | male |
| *Acadm* | -0.54 | 3.83E-49 | down | age | endothelial | male |
| *Atp5b* | -0.55 | 1.62E-48 | down | age | endothelial | male |
| *Atp5g3* | -0.57 | 2.58E-57 | down | age | endothelial | male |
| *Rbm20* | -0.59 | 5.92E-26 | down | age | endothelial | male |
| *Ckmt2* | -0.59 | 1.28E-62 | down | age | endothelial | male |
| *Filip1l* | -0.60 | 2.16E-47 | down | age | endothelial | male |
| *Cox6c* | -0.60 | 3.68E-60 | down | age | endothelial | male |
| *Gm15564* | -0.61 | 9.16E-30 | down | age | endothelial | male |
| *Ndufa4* | -0.62 | 2.82E-64 | down | age | endothelial | male |
| *Gsn* | -0.63 | 3.30E-72 | down | age | endothelial | male |
| *Myl3* | -0.64 | 3.10E-97 | down | age | endothelial | male |
| *Lpl* | -0.66 | 1.62E-133 | down | age | endothelial | male |
| *Fhl2* | -0.67 | 4.89E-40 | down | age | endothelial | male |
| *Fabp3* | -0.68 | 8.22E-116 | down | age | endothelial | male |
| *Cox6a2* | -0.69 | 2.33E-105 | down | age | endothelial | male |
| *Tnni3* | -0.71 | 1.03E-130 | down | age | endothelial | male |
| *Mb* | -0.72 | 8.26E-183 | down | age | endothelial | male |
| *Myl2* | -0.74 | 1.14E-159 | down | age | endothelial | male |
| *Hbb-bt* | -0.76 | 2.94E-145 | down | age | endothelial | male |
| *Ryr2* | -0.79 | 2.96E-115 | down | age | endothelial | male |
| *Actc1* | -0.79 | 4.50E-187 | down | age | endothelial | male |
| *Il31ra* | -0.80 | 5.33E-94 | down | age | endothelial | male |
| *Hba-a1* | -0.81 | 5.74E-197 | down | age | endothelial | male |
| *Atp2a2* | -0.81 | 2.37E-201 | down | age | endothelial | male |
| *Tnnt2* | -0.81 | 4.53E-203 | down | age | endothelial | male |
| *Tpm1* | -0.82 | 1.11E-264 | down | age | endothelial | male |
| *Hba-a2* | -0.83 | 1.33E-189 | down | age | endothelial | male |
| *Hbb-bs* | -0.84 | 0.00E+00 | down | age | endothelial | male |
| *Pln* | -0.88 | 9.10E-221 | down | age | endothelial | male |
| *Ttn* | -0.90 | 0.00E+00 | down | age | endothelial | male |
| *Camk1d* | -0.91 | 3.91E-254 | down | age | endothelial | male |
| *Myh6* | -0.99 | 0.00E+00 | down | age | endothelial | male |
| *Lars2* | -1.05 | 0.00E+00 | down | age | endothelial | male |
| *Gm42418* | -1.24 | 0.00E+00 | down | age | endothelial | male |
| *Cmss1* | -1.26 | 0.00E+00 | down | age | endothelial | male |
| *AY036118* | -1.60 | 0.00E+00 | down | age | endothelial | male |
| *Hba-a1* | 1.83 | 1.33E-83 | up | age | endothelial | female |
| *Hba-a2* | 1.82 | 1.03E-78 | up | age | endothelial | female |
| *Hbb-bs* | 1.67 | 6.83E-108 | up | age | endothelial | female |
| *Hbb-bt* | 1.47 | 2.51E-51 | up | age | endothelial | female |
| *C3* | 1.24 | 1.43E-21 | up | age | endothelial | female |
| *Upk3b* | 1.02 | 2.20E-10 | up | age | endothelial | female |
| *Igfbp6* | 0.95 | 1.09E-09 | up | age | endothelial | female |
| *Flrt2* | 0.91 | 5.39E-06 | up | age | endothelial | female |
| *Dcn* | 0.91 | 1.25E-29 | up | age | endothelial | female |
| *Aebp1* | 0.91 | 3.24E-11 | up | age | endothelial | female |
| *Rarres2* | 0.88 | 1.02E-10 | up | age | endothelial | female |
| *Abi1* | 0.88 | 1.54E-10 | up | age | endothelial | female |
| *Cfh* | 0.85 | 8.32E-23 | up | age | endothelial | female |
| *Ly6e* | 0.84 | 4.27E-14 | up | age | endothelial | female |
| *Igfbp5* | 0.84 | 1.50E-10 | up | age | endothelial | female |
| *Col3a1* | 0.83 | 1.00E-08 | up | age | endothelial | female |
| *Bicc1* | 0.81 | 2.64E-05 | up | age | endothelial | female |
| *Npr3* | 0.80 | 1.28E-14 | up | age | endothelial | female |
| *S100a6* | 0.78 | 7.78E-10 | up | age | endothelial | female |
| *Mgp* | 0.75 | 4.41E-12 | up | age | endothelial | female |
| *Crip1* | 0.74 | 2.03E-09 | up | age | endothelial | female |
| *Trf* | 0.73 | 1.64E-11 | up | age | endothelial | female |
| *Plxna4* | 0.73 | 1.82E-04 | up | age | endothelial | female |
| *Col1a2* | 0.72 | 1.64E-07 | up | age | endothelial | female |
| *Sulf1* | 0.71 | 2.41E-08 | up | age | endothelial | female |
| *Efemp1* | 0.70 | 4.83E-05 | up | age | endothelial | female |
| *Pkhd1l1* | 0.69 | 6.31E-06 | up | age | endothelial | female |
| *Serping1* | 0.69 | 1.14E-08 | up | age | endothelial | female |
| *Pabpc1* | 0.68 | 1.62E-02 | up | age | endothelial | female |
| *Tm4sf1* | 0.68 | 1.42E-10 | up | age | endothelial | female |
| *Muc16* | 0.68 | 9.58E-03 | up | age | endothelial | female |
| *Aqp1* | 0.66 | 6.13E-06 | up | age | endothelial | female |
| *H2-D1* | 0.66 | 2.49E-06 | up | age | endothelial | female |
| *Fabp5* | 0.65 | 2.18E-07 | up | age | endothelial | female |
| *Gpm6a* | 0.64 | 4.52E-11 | up | age | endothelial | female |
| *Sparc* | 0.63 | 3.09E-06 | up | age | endothelial | female |
| *Timp2* | 0.63 | 9.72E-07 | up | age | endothelial | female |
| *App* | 0.63 | 4.33E-06 | up | age | endothelial | female |
| *B2m* | 0.63 | 4.44E-06 | up | age | endothelial | female |
| *Crim1* | 0.62 | 4.72E-06 | up | age | endothelial | female |
| *Malat1* | 0.61 | 6.24E-47 | up | age | endothelial | female |
| *Fmo2* | 0.61 | 3.40E-03 | up | age | endothelial | female |
| *Pdia3* | 0.60 | 8.48E-08 | up | age | endothelial | female |
| *Cd2ap* | 0.60 | 1.57E-02 | up | age | endothelial | female |
| *Apoe* | 0.60 | 4.26E-13 | up | age | endothelial | female |
| *Gpx1* | 0.59 | 7.85E-07 | up | age | endothelial | female |
| *Vim* | 0.59 | 1.18E-07 | up | age | endothelial | female |
| *Actg1* | 0.58 | 3.43E-05 | up | age | endothelial | female |
| *Pecam1* | 0.58 | 4.06E-06 | up | age | endothelial | female |
| *Selenop* | 0.57 | 8.68E-05 | up | age | endothelial | female |
| *Cavin1* | 0.56 | 1.32E-04 | up | age | endothelial | female |
| *Eng* | 0.56 | 4.88E-05 | up | age | endothelial | female |
| *Ltbp4* | 0.55 | 5.39E-04 | up | age | endothelial | female |
| *Fbln2* | 0.54 | 1.95E-05 | up | age | endothelial | female |
| *Tgm2* | 0.54 | 5.75E-04 | up | age | endothelial | female |
| *Fbln1* | 0.54 | 3.03E-04 | up | age | endothelial | female |
| *Actb* | 0.53 | 3.89E-08 | up | age | endothelial | female |
| *Marcks* | 0.53 | 7.57E-03 | up | age | endothelial | female |
| *Prkg1* | 0.53 | 5.16E-03 | up | age | endothelial | female |
| *Rnase4* | 0.53 | 2.07E-04 | up | age | endothelial | female |
| *Tmsb10* | 0.52 | 1.80E-02 | up | age | endothelial | female |
| *Pnisr* | 0.51 | 2.08E-04 | up | age | endothelial | female |
| *Cgnl1* | 0.51 | 2.97E-02 | up | age | endothelial | female |
| *Ifitm3* | 0.51 | 3.64E-02 | up | age | endothelial | female |
| *Arhgap29* | 0.50 | 4.34E-03 | up | age | endothelial | female |
| *Ftl1* | 0.50 | 1.91E-03 | up | age | endothelial | female |
| *Heg1* | 0.50 | 8.85E-04 | up | age | endothelial | female |
| *Gnai2* | 0.49 | 2.96E-04 | up | age | endothelial | female |
| *Foxp1* | 0.49 | 1.92E-04 | up | age | endothelial | female |
| *Lyz2* | 0.48 | 3.12E-04 | up | age | endothelial | female |
| *Ifitm2* | 0.48 | 1.89E-06 | up | age | endothelial | female |
| *Cd47* | 0.48 | 9.70E-03 | up | age | endothelial | female |
| *Rbms1* | 0.48 | 2.03E-04 | up | age | endothelial | female |
| *Man2a1* | 0.48 | 3.91E-03 | up | age | endothelial | female |
| *Fbn1* | 0.47 | 1.05E-02 | up | age | endothelial | female |
| *Igfbp7* | 0.47 | 1.66E-04 | up | age | endothelial | female |
| *Igfbp4* | 0.47 | 1.97E-02 | up | age | endothelial | female |
| *Rsrp1* | 0.47 | 6.20E-04 | up | age | endothelial | female |
| *Kdr* | 0.47 | 2.12E-03 | up | age | endothelial | female |
| *H2-K1* | 0.46 | 2.65E-05 | up | age | endothelial | female |
| *Dync1i2* | 0.46 | 6.28E-04 | up | age | endothelial | female |
| *Sntb2* | 0.46 | 8.53E-03 | up | age | endothelial | female |
| *Rapgef5* | 0.46 | 5.23E-04 | up | age | endothelial | female |
| *Mgst1* | 0.45 | 2.42E-07 | up | age | endothelial | female |
| *Aplp2* | 0.45 | 7.71E-04 | up | age | endothelial | female |
| *Zfp36l2* | 0.45 | 9.03E-04 | up | age | endothelial | female |
| *Kcnt2* | 0.45 | 2.14E-02 | up | age | endothelial | female |
| *Cst3* | 0.44 | 4.06E-06 | up | age | endothelial | female |
| *Il6st* | 0.44 | 9.74E-03 | up | age | endothelial | female |
| *Cd9* | 0.43 | 3.99E-02 | up | age | endothelial | female |
| *Eef1a1* | 0.43 | 2.38E-02 | up | age | endothelial | female |
| *Airn* | 0.43 | 5.50E-06 | up | age | endothelial | female |
| *Srsf11* | 0.43 | 7.54E-04 | up | age | endothelial | female |
| *Ctsl* | 0.41 | 3.11E-03 | up | age | endothelial | female |
| *Elmo1* | 0.41 | 1.39E-02 | up | age | endothelial | female |
| *Rps28* | 0.40 | 3.01E-02 | up | age | endothelial | female |
| *H3f3a* | 0.40 | 3.16E-04 | up | age | endothelial | female |
| *Itm2b* | 0.40 | 2.33E-06 | up | age | endothelial | female |
| *Fabp4* | 0.40 | 7.00E-06 | up | age | endothelial | female |
| *Abca8a* | 0.39 | 4.85E-03 | up | age | endothelial | female |
| *Col4a2* | 0.39 | 8.46E-03 | up | age | endothelial | female |
| *Smoc2* | 0.39 | 1.16E-02 | up | age | endothelial | female |
| *Dab2* | 0.37 | 1.23E-02 | up | age | endothelial | female |
| *Ubb* | 0.37 | 2.00E-04 | up | age | endothelial | female |
| *Uba52* | 0.36 | 3.86E-02 | up | age | endothelial | female |
| *Ryr2* | -0.39 | 7.48E-15 | down | age | endothelial | female |
| *Pde4dip* | -0.40 | 1.28E-04 | down | age | endothelial | female |
| *Dst* | -0.44 | 3.32E-08 | down | age | endothelial | female |
| *Xirp2* | -0.45 | 1.53E-10 | down | age | endothelial | female |
| *Golga4* | -0.46 | 4.79E-04 | down | age | endothelial | female |
| *Filip1l* | -0.47 | 7.96E-10 | down | age | endothelial | female |
| *Cmya5* | -0.49 | 1.24E-07 | down | age | endothelial | female |
| *Kif1c* | -0.52 | 1.74E-03 | down | age | endothelial | female |
| *AY036118* | -0.55 | 1.25E-21 | down | age | endothelial | female |
| *Cdk8* | -0.56 | 1.60E-06 | down | age | endothelial | female |
| *Gm42418* | -0.59 | 7.79E-69 | down | age | endothelial | female |
| *Obscn* | -0.59 | 4.83E-10 | down | age | endothelial | female |
| *Gm15564* | -0.62 | 2.84E-10 | down | age | endothelial | female |
| *Myo18b* | -0.63 | 7.51E-07 | down | age | endothelial | female |
| *Cmss1* | -0.65 | 6.77E-34 | down | age | endothelial | female |
| *Pam* | -0.66 | 2.98E-03 | down | age | endothelial | female |
| *Sln* | -0.69 | 5.12E-04 | down | age | endothelial | female |
| *Lars2* | -0.72 | 6.96E-45 | down | age | endothelial | female |
| *Gphn* | -0.72 | 8.44E-39 | down | age | endothelial | female |
| *Kcnj3* | -0.72 | 8.08E-06 | down | age | endothelial | female |
| *Camk1d* | -0.73 | 1.52E-61 | down | age | endothelial | female |
| *Nppa* | -0.76 | 1.94E-05 | down | age | endothelial | female |
| *Ttn* | -0.78 | 4.98E-42 | down | age | endothelial | female |
| *Myl4* | -0.99 | 1.26E-04 | down | age | endothelial | female |
| *Myl7* | -1.08 | 1.39E-06 | down | age | endothelial | female |

**Table S11.** Top ten enriched Gene Ontology terms for Biological Process (GO BP) based on age up- and down-regulated DEGs in endothelial cells; snRNA-seq, both sex combined, adj.p-value<0.05, |log2FC| >0.36

| **Term** | **Overlap** | **Adj.P-value** | **Genes** | **comparison** | **cell_type** | **direction** |
| --- | --- | --- | --- | --- | --- | --- |
| Regulation of Complement Activation, Alternative Pathway (GO:0030451) | 1/7 | 0.009801366 | CFH | aging | endothelial | up,both |
| Regulation of Cell Killing (GO:0031341) | 1/9 | 0.009801366 | CFH | aging | endothelial | up,both |
| Complement Activation, Alternative Pathway (GO:0006957) | 1/9 | 0.009801366 | CFH | aging | endothelial | up,both |
| Axon Ensheathment in Central Nervous System (GO:0032291) | 1/10 | 0.009801366 | CFH | aging | endothelial | up,both |
| Regulation of Complement-Dependent Cytotoxicity (GO:1903659) | 1/11 | 0.009801366 | CFH | aging | endothelial | up,both |
| Potassium Ion Export Across Plasma Membrane (GO:0097623) | 1/13 | 0.009801366 | KCNT2 | aging | endothelial | up,both |
| Central Nervous System Myelination (GO:0022010) | 1/14 | 0.009801366 | CFH | aging | endothelial | up,both |
| Regulation of Immune Effector Process (GO:0002697) | 1/16 | 0.009801366 | CFH | aging | endothelial | up,both |
| Regulation of Humoral Immune Response (GO:0002920) | 1/16 | 0.009801366 | CFH | aging | endothelial | up,both |
| Oligodendrocyte Development (GO:0014003) | 1/17 | 0.009801366 | CFH | aging | endothelial | up,both |
| Response to Muscle Stretch (GO:0035994) | 3/15 | 2.28087E-05 | RYR2;NPPA;TTN | aging | endothelial | down, both |
| Positive Regulation of Heart Rate (GO:0010460) | 3/16 | 2.28087E-05 | RYR2;NPPA;ATP2A2 | aging | endothelial | down, both |
| Regulation of Heart Contraction (GO:0008016) | 4/73 | 2.28087E-05 | RYR2;KCND2;ATP2A2;MYH6 | aging | endothelial | down, both |
| Positive Regulation of Heart Contraction (GO:0045823) | 3/23 | 4.38301E-05 | RYR2;NPPA;ATP2A2 | aging | endothelial | down, both |
| Cardiac Muscle Contraction (GO:0060048) | 3/35 | 0.00012883 | RYR2;MYH6;TTN | aging | endothelial | down, both |
| Muscle Contraction (GO:0006936) | 4/145 | 0.000140751 | RYR2;KCND2;MYH6;TTN | aging | endothelial | down, both |
| Heart Contraction (GO:0060047) | 3/42 | 0.000140751 | RYR2;MYH6;TTN | aging | endothelial | down, both |
| Regulation of Cardiac Muscle Contraction (GO:0055117) | 3/42 | 0.000140751 | RYR2;NPPA;ATP2A2 | aging | endothelial | down, both |
| Striated Muscle Hypertrophy (GO:0014897) | 2/6 | 0.000237553 | RYR2;TTN | aging | endothelial | down, both |
| Cardiac Muscle Hypertrophy (GO:0003300) | 2/8 | 0.000398719 | RYR2;TTN | aging | endothelial | down, both |

**Table S12.** Top ten enriched Gene Ontology terms for Biological Process (GO BP) based on age up- and down-regulated DEGs in *Cdkn1a*^+^ vs *Cdkn1a*^⎯^ *Gsn*^+^ fibroblasts; snRNA-seq, both age & sex combined, adj.p-value<0.05, |log2FC| >0.36

| **Term** | **Overlap** | **Adj.P.value** | **Genes** | **GeneRatio** | **comparison** | **cell_type** | **direction** |
| --- | --- | --- | --- | --- | --- | --- | --- |
| Positive Regulation of miRNA Transcription (GO:1902895) | 4/47 | 1.54E-02 | EGR1;POU2F1;SMAD3;EGFR | 0.05 | *Cdkn1a* | Gsn+ Fibroblast | up, both |
| Establishment of Epithelial Cell Polarity (GO:0090162) | 3/18 | 1.54E-02 | CYTH3;PARD3;MYO9A | 0.04 | *Cdkn1a* | Gsn+ Fibroblast | up, both |
| Positive Regulation of miRNA Metabolic Process (GO:2000630) | 4/53 | 1.54E-02 | EGR1;POU2F1;SMAD3;EGFR | 0.05 | *Cdkn1a* | Gsn+ Fibroblast | up, both |
| Regulation of miRNA Transcription (GO:1902893) | 4/66 | 2.54E-02 | EGR1;POU2F1;SMAD3;EGFR | 0.05 | *Cdkn1a* | Gsn+ Fibroblast | up, both |
| Regulation of Fat Cell Differentiation (GO:0045598) | 4/75 | 2.54E-02 | FTO;SMAD3;MEDAG;CMKLR1 | 0.05 | *Cdkn1a* | Gsn+ Fibroblast | up, both |
| Regulation of Phosphatidylinositol 3-Kinase/Protein Kinase B Signal Transduction (GO:0051896) | 6/219 | 2.54E-02 | IL1R1;MAGI2;GAS6;RTN4;EGFR;IGF1R | 0.07 | *Cdkn1a* | Gsn+ Fibroblast | up, both |
| Endodermal Cell Differentiation (GO:0035987) | 3/32 | 2.54E-02 | COL4A2;MMP2;ITGA5 | 0.04 | *Cdkn1a* | Gsn+ Fibroblast | up, both |
| Establishment of Centrosome Localization (GO:0051660) | 2/7 | 2.54E-02 | PARD3;PARD3B | 0.02 | *Cdkn1a* | Gsn+ Fibroblast | up, both |
| Cellular Response to Growth Factor Stimulus (GO:0071363) | 5/150 | 2.54E-02 | WWOX;SMAD3;MAGI2;EGFR;SOX5 | 0.06 | *Cdkn1a* | Gsn+ Fibroblast | up, both |
| Response to Transforming Growth Factor Beta (GO:0071559) | 3/35 | 2.54E-02 | WWOX;SMAD3;SOX5 | 0.04 | *Cdkn1a* | Gsn+ Fibroblast | up, both |
| Ventricular Cardiac Muscle Tissue Development (GO:0003229) | 5/24 | 6.46E-10 | TNNT2;MYL2;TPM1;TNNI3;MYH6 | 0.33 | *Cdkn1a* | Gsn+ Fibroblast | down, both |
| Cardiac Muscle Tissue Morphogenesis (GO:0055008) | 5/30 | 1.05E-09 | TNNT2;MYL2;TPM1;TNNI3;MYH6 | 0.33 | *Cdkn1a* | Gsn+ Fibroblast | down, both |
| Ventricular Cardiac Muscle Tissue Morphogenesis (GO:0055010) | 5/33 | 1.05E-09 | TNNT2;MYL2;TPM1;TNNI3;MYH6 | 0.33 | *Cdkn1a* | Gsn+ Fibroblast | down, both |
| Cardiac Muscle Contraction (GO:0060048) | 5/34 | 1.05E-09 | TNNT2;MYL2;TPM1;TNNI3;MYH6 | 0.33 | *Cdkn1a* | Gsn+ Fibroblast | down, both |
| Heart Contraction (GO:0060047) | 5/41 | 2.14E-09 | TNNT2;MYL2;TPM1;TNNI3;MYH6 | 0.33 | *Cdkn1a* | Gsn+ Fibroblast | down, both |
| Cardiac Ventricle Morphogenesis (GO:0003208) | 5/42 | 2.14E-09 | TNNT2;MYL2;TPM1;TNNI3;MYH6 | 0.33 | *Cdkn1a* | Gsn+ Fibroblast | down, both |
| Muscle Filament Sliding (GO:0030049) | 4/13 | 2.83E-09 | TNNT2;TPM1;TNNI3;MYH6 | 0.27 | *Cdkn1a* | Gsn+ Fibroblast | down, both |
| Actin-Myosin Filament Sliding (GO:0033275) | 4/15 | 4.73E-09 | TNNT2;TPM1;TNNI3;MYH6 | 0.27 | *Cdkn1a* | Gsn+ Fibroblast | down, both |
| Striated Muscle Contraction (GO:0006941) | 5/55 | 5.80E-09 | TNNT2;MYL2;TPM1;TNNI3;MYH6 | 0.33 | *Cdkn1a* | Gsn+ Fibroblast | down, both |
| Myofibril Assembly (GO:0030239) | 4/51 | 6.82E-07 | TNNT2;MYL2;TPM1;MYH6 | 0.27 | *Cdkn1a* | Gsn+ Fibroblast | down, both |
